# Design, Synthesis, and *In Silico* Studies of New Norfloxacin Analogues with Broad Spectrum Antibacterial Activity *via* Topoisomerase II Inhibition

**DOI:** 10.3390/ph18040545

**Published:** 2025-04-08

**Authors:** Ahmed M. El-Saghier, Laila Abosella, Abdelfattah Hassan, Esmail O. Elakesh, Stefan Bräse, Gamal El-Din A. Abuo-Rahma, Hossameldin A. Aziz

**Affiliations:** 1 Chemistry Department, Faculty of Science, Sohag University, Sohag 82524, Egypt; lailaabosella@gmail.com; 2 Medicinal Chemistry Department, Faculty of Pharmacy—Al-Jmail, Sabratha University, Sabratha P.O. Box 250, Libya; 3 Medicinal Chemistry Department, Faculty of Pharmacy, South Valley University, Qena 83523, Egypt; abdelfattah_hassan@svu.edu.eg; 4 Medicinal Chemistry Department, Faculty of Pharmacy, National South Valley University, Qena 83523, Egypt; 5 Chemistry Department, Faculty of Science, University of Zawia, Al Zawiya 16418, Libya; esmail_elakesh@zu.edu.ly; 6 Institute for Biological and Chemical System, Karlsruhe Institute of Technology, 76131 Karlsruhe, Germany; 7 Medicinal Chemistry Department, Faculty of Pharmacy, Minia University, Minia 61519, Egypt; gamal.aborahma@mu.edu.eg; 8 Pharmaceutical Chemistry Department, Faculty of Pharmacy, Deraya University, New Minia-61768, Egypt; 9 Pharmaceutical Chemistry Department, Faculty of Pharmacy, New Vallely University, New Valley 72511, Egypt; hossamaziz85@pha.nvu.edu.eg

**Keywords:** norfloxacin, antibacterial, topoisomerase II, *MRSA*

## Abstract

**Background**: Novel norfloxacin derivatives were synthesized, characterized, and screened for their antibacterial activity against Gram-positive strain *S. aureus ATCC 6538* and Gram-negative strains; *E. coli ATCC 25923*, *K. pneumoniae ATCC 10031*, and *P. aeruginosa ATCC 27853* using the agar cup diffusion method. **Results**: The results revealed that compounds **6**–**17** exhibited more potent activity towards *S. aureus ATCC 6538* with MIC values of 0.21–3.61 µM than norfloxacin with a MIC of 7.83 µM. The most potent compound, **6**, showed 37-fold more potency than norfloxacin. More importantly, compound **7** exhibited more potent activity against *MRSA* than norfloxacin, with MIC values of 0.80 and 1.96 µM, respectively. Meanwhile, compounds **15** and **16** have potent activity towards the Gram-negative strains with MIC values of 0.20–0.79 µM compared with norfloxacin with a MIC of 0.24 µM. Moreover, the potent compounds showed higher activity towards topoisomerase II enzymes, especially against topoisomerase IV, which confirms the docking study with the *S. aureus* gyrase enzyme active binding site (PDB ID: 2XCT). In addition, cytotoxicity assays of the most potent compounds showed that compounds **6**, **7**, **15**, and **16** have negligible risks of toxic effects when evaluated against the normal cell line WI 38. **Conclusions**: The docking study of the most potent compounds **6**, **7**, **15**, and **16** on the gyrase enzyme active site (PDB: 2XCT) aligns their antibacterial activity and topoisomerase inhibition. The physicochemical and pharmacokinetic characteristics of the target derivatives were forecasted via SwissADME. Hence, these compounds are considered promising antibacterial candidates that require further optimization.

## 1. Introduction

Fluoroquinolones are an essential group of antibiotics extensively employed to manage various bacterial diseases, such as skin, bone, soft tissue, and sexually transmitted infections, alongside community-acquired pneumonia [1,2,3,4,5]. Additionally, they demonstrate a range of non-classical biological properties such as anticancer, antitubercular, antiviral, anti-HIV, antimalarial, anti-inflammatory, and anti-Alzheimer activities [6,7,8,9,10,11,12,13]. Fluoroquinolones function by inhibiting two crucial bacterial enzymes, DNA gyrase and topoisomerase IV, that play a fundamental role in DNA replication and bacterial growth. Thus, fluoroquinolones are regarded as bactericidal agents [14], and due to the therapeutic success of fluoroquinolones, drug discovery and development introduced many new derivatives of fluoroquinolones with better properties [7,8]. However, as with most antibacterial treatments, the unchecked and widespread use of these medications led to the growth of bacterial resistance [7,15]. Hence, developing novel antibacterial agents or modifying existing ones is crucial to combat bacterial resistance while maintaining wide antibacterial efficacy and their safety profile [16].

Norfloxacin, a second-generation fluoroquinolone antibiotic, is extensively utilized to address a variety of bacterial ailments. It exhibits a wide spectrum of effectiveness against Gram-negative and Gram-positive bacteria while demonstrating a favorable safety profile [17,18]. Norfloxacin, as well as other fluoroquinolones, contain a carboxyl group and a core piperazinyl group. The presence of the piperazinyl group makes the molecule an excellent core for introducing different modifications to the structure aiming to produce new derivatives, which can combat the developed resistance and trigger the potency and efficacy of the parent molecule without affecting the safety profile [19,20,21,22].

The World Health Organization (WHO) classifies the most resistant bacteria into three groups based on the need for the development of new antibiotics. Among these pathogens, certain strains exhibit resistance to fluoroquinolones. Bacteria develop resistance to fluoroquinolones by modifications in target enzymes, changes in drug penetration (in both Gram-positive and Gram-negative bacteria), and plasmid acquisition. Over time, resistance to fluoroquinolones emerged concurrently with researchers’ attempts to improve the compounds within this class [23].

**Figure 1:**
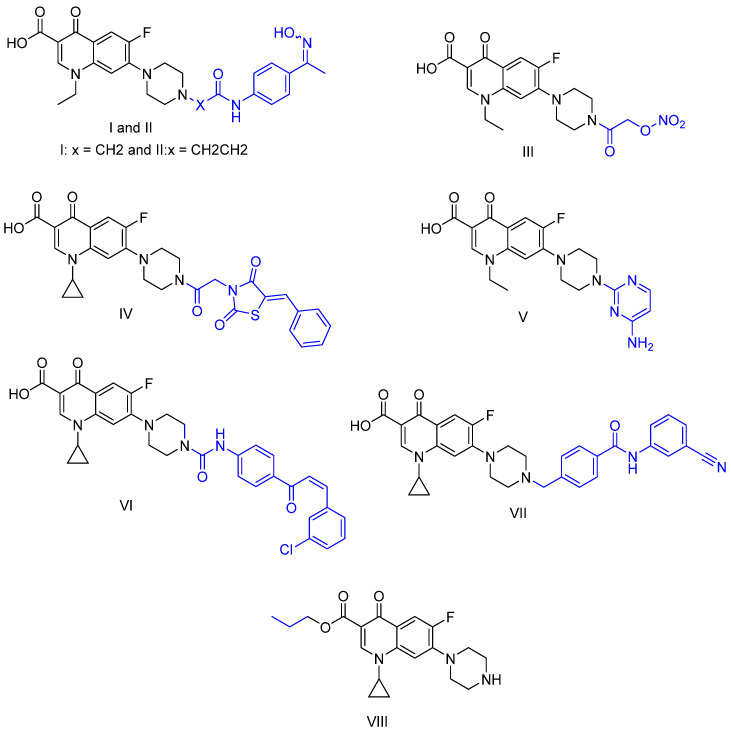
Structure of different fluoroquinolone hybrids (**I**–**VIII**) with antibacterial activity.

It was found that *N*-4-piperazinyl substitution of fluoroquinolones improves their physiochemical characteristics, increases their lipophilicity, increases their activity towards Gram-positive germs, and primarily maintains their activity towards Gram-negative pathogens [22,24] in addition to combating the developed resistance by improving the physiochemical properties or by enhancing the activity toward topoisomerases enzymes [22]. A range of *N*-piperazinyl substituents were synthesized and assessed for their antimicrobial efficacy in this particular setting [7,8,22,25,26,27]. The new derivatives demonstrated enhanced efficiency in combating infections caused by Gram-positive and Gram-negative bacteria, similar to compounds I–VII, which are shown in Figure 1 [7,8,22,25,26,27]. The norfloxacin/oxime hybrids I and II, which provide nitric oxide, have potent activity against *K. pneumonia* with MIC values of 0.08 µM and 0.034 µM, respectively [8]. On the other hand, the nitrate ester derivative of norfloxacin III demonstrates antitubercular activity with a MIC value of 7.4 µM against *M. tuberculosis* [7]. The ciprofloxacin/thiazolidinedione derivative IV exhibits significant antibacterial activity towards *S. aureus ATCC 6538*, with a MIC value of 0.02 µM [22]. Also, the aminopyrimidine derivative of norfloxacin V exhibited significant activity against *E. faecalis*, with a low MIC of 0.25 µg/mL [25]. In contrast, the amide of ciprofloxacin VI demonstrated superior antibacterial efficacy against *S. aureus* and *E. coli*, with MIC values of 0.06 and 0.18 µg/mL, respectively [27]. Compound VII demonstrated superior efficacy against *MRSA* and *S. aureus*, with a MIC value of 0.0002 µg/mL [26]. Compound VIII had strong antibacterial activity against penicillin-resistant *E. coli* with a MIC value of 1.0 µg/mL [28]. Furthermore, including substitution at position 3 of the fluoroquinolone molecule may boost its antimicrobial activity, particularly towards Gram-negative pathogens, as observed in compound VIII [28].

Based on the aspects mentioned above and to an extension of our work in the simple synthesis of analogous norfloxacin with an expected biological activity, this research aimed to synthesize a novel *N*-4-piperazinyl derivative of norfloxacin. The design of the target compounds depends upon being diverse in chemical properties; they involve aliphatic, saturated, or unsaturated aromatic and heterocyclic to see the impact on biological activity. Although the main scope for our work was the synthesis of an N-4-piperazinyl derivative with more potent activity against Gram-positive strain *S. aureus*, we synthesized two norfloxacin derivatives **15** and its acylated derivative **16** with a substitution on the carboxylic at C-3 to compare their activity against Gram-negative strains with other N-4-derivatives to emphasize that the C-3-carboxylic derivative of norfloxacin has more potent activity against Gram-negative strains than the N-4-piperazinyl derivative that was originally present in this study. Moreover, we believed that the electrophilic nature of compound 16 enhanced the antibacterial activity, which was clear in the MIC values of compound 16 [22,23]. Various spectroscopic techniques such as IR, NMR, and spectrometric tool elemental analyses verified the newly synthesized compounds. As a result, the target compounds were tested to determine their antibacterial properties were assessed towards *S. aureus ATCC 6538* as a Gram-positive strain as well as the Gram-negative organisms *P. aeruginosa ATCC 27853*, *E. coli ATCC 25922*, and *K. pneumoniae ATCC 10031*, using the conventional agar cup diffusion method. The antibacterial compounds with the highest potency against *S. aureus ATCC 6538* were tested for their effectiveness against *MRSA*. Moreover, the most potent antibacterial agents were investigated for their capacity to hinder the activity of topoisomerase II, specifically the gyrase and topoisomerase IV enzymes. Therefore, compounds having the capacity to inhibit gyrase and topoisomerase II enzymes were computationally positioned in the DNA gyrase enzyme active site to examine potential interactions with the enzyme’s active site, similar to those induced by the original medication norfloxacin. Finally, the compounds with the most potent antibacterial activity against the tested Gram-positive or Gram-negative strains were evaluated against the normal cell line WI 38 to evaluate their selectivity toward bacterial strains and their safety profile.

## 2. Results and Discussion

### 2.1. Chemistry

**Scheme 1:**
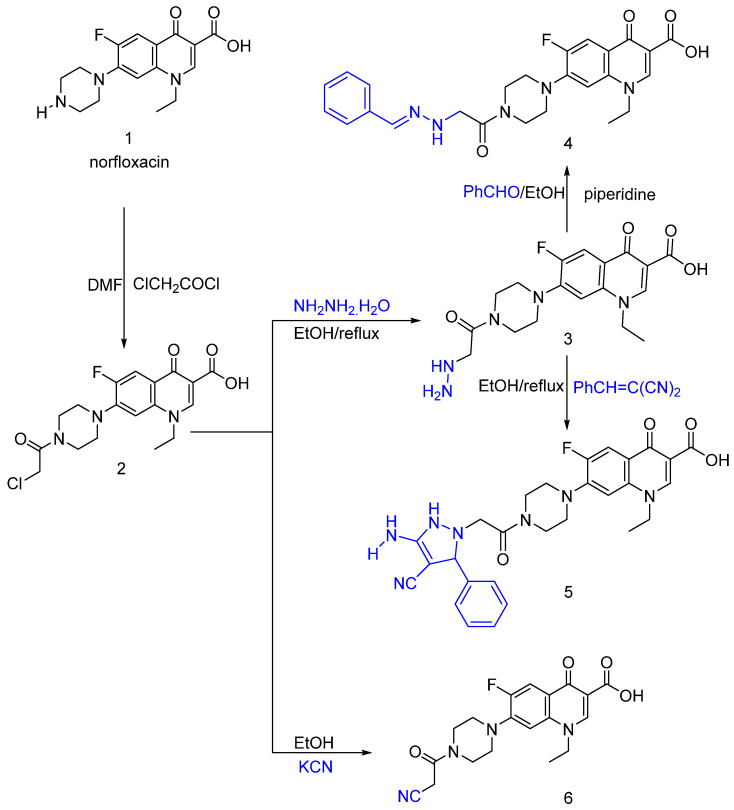
Synthesis of norfloxacin derivatives **2**–**6**.

**Scheme 2:**
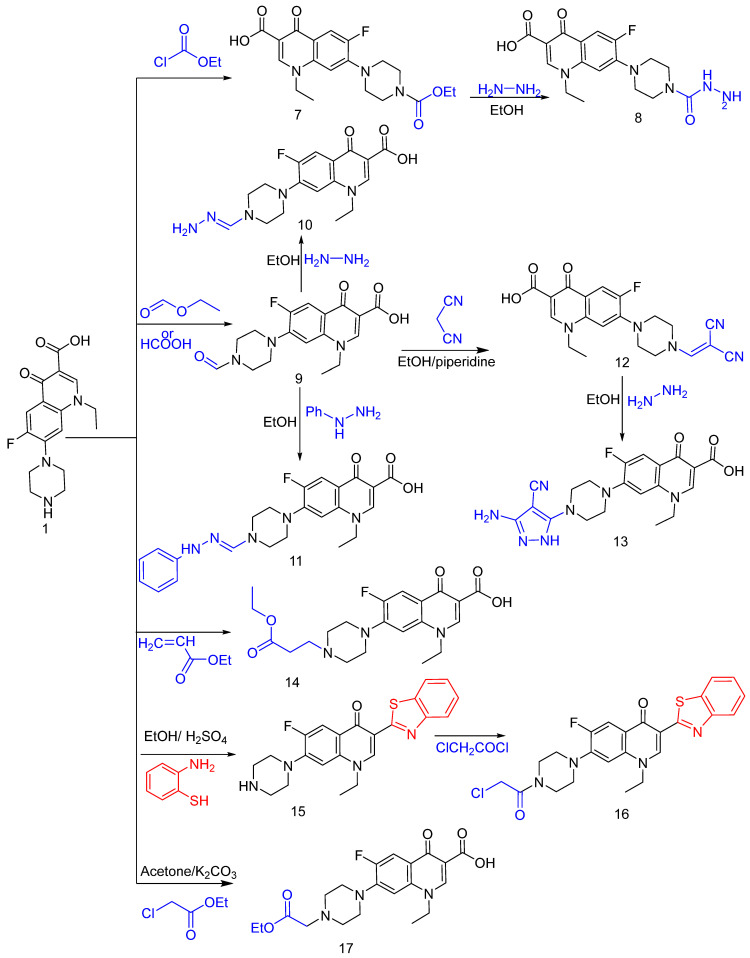
Synthesis of norfloxacin derivative **7**–**17**.

In continuation of our work for the synthesis of new biologically active heterocyclic compounds and analogues to norfloxacin [29,30], we used the key intermediate N-chloroacetyl norfloxacin as a precursor for the synthesis of the target norfloxacin derivatives as illustrated in (Scheme 1 and Scheme 2). Importantly, the key intermediate *N*-Chloroacetyl norfloxacin **2** was prepared as reported [31]. The hydrazine derivative **3** was produced by allowing compound **2** to react with hydrazine hydrate. Compound **3**’s infrared spectra showed extra bands at 3462, 3340, and 3190 cm^–1^, which were related to the NH and NH_2_ groups. In addition to the characteristic features of norfloxacin [32], the ^1^H-NMR showed new signals for NH and NH_2_ at δ 4.13 ppm and 3.80 ppm, respectively. Compound **3** was reacted with benzaldehyde and benzylidene malononitrile to afford compounds **4** and **5**, respectively. The IR spectra showed the disappearance of the NH_2_ band in compound **4** and the presence of a new band at 2210 cm^−1^ for the cyano group in compound **5**. The ^1^H-NMR showed the absence of the amino group and downfield shift of (NH-N=CH-C_6_H_5_) to 6.72 ppm in compound **4** and the presence of new signals for aromatic hydrogens at 7.00–8.00 ppm in the spectra of both compounds **4** and **5**. Also, the HNMR spectra of compounds **4** and **5** showed singlet signals of NCH_2_CO at 5.41 and 4.13 ppm, respectively. The proposed mechanism for the formation of compound **5** is via a nucleophilic attack of the NH_2_ of the hydrazine moiety of compound **3** at the ethylenic bond of the benzylidene malononitrile followed by cyclization to afford the amino pyrazole moiety. A substitution reaction of compound **2** with potassium cyanide afforded cyano derivative **6** (Scheme 1) [33]. The ^1^H-NMR spectrum of compound **6** showed an upfield shift of the methylene of COCH_2_CN to δ 4.13 ppm.

Additionally, the *N-methoxycarbonyl* derivative **7** was produced by allowing norfloxacin to react with ethyl chloroformate via a nucleophilic substitution reaction at the piperazinyl NH group of the norfloxacin. The infrared spectrum of compound **7** showed a new band at 1730 cm^−1^ related to the carbonyl ester and no band at 3170 cm^−1^ linked to the norfloxacin NH group. The ^1^H-NMR showed the absence of the signal to piperazinyl NH proton and the presence of new signals at δ 4.20 ppm and 1.40 ppm assigned to the CH_2_ and CH_3_ groups of the ester moiety, respectively. The hydrazide **8** was produced by allowing compound **7** to react with hydrazine hydrate. ^1^HNMR of compound **8** showed the appearance of two signals at δ 4.08 and 8.50 ppm related to NH_2_ and NH, respectively. *N*-formylation of norfloxacin afforded compound **9** was formed via a reaction of norfloxacin with formic acid or with ethyl formate. The IR spectrum showed a new band at 1710 cm^−1^ assigned to the formyl carbonyl group, and ^1^HNMR showed a new singlet signal at 8.98 ppm for aldehydic proton. Also, the hydrazone derivatives **10** and **11** were produced by reaction of compound **9** with hydrazine hydrate and phenyl hydrazine, respectively. ^1^HNMR of compounds **10** and **11** showed singlets of NCH=N at δ 8.17 and 8.13 ppm, respectively. Moreover, condensation of **9** with malononitrile afforded acrylonitrile derivative **12**. The IR spectrum **12** showed new bands at 2200 and 2222 cm^−1^ assigned to the two cyano groups. Also, ^1^HNMR of compound **12** showed singlets of δ NCH=(CN)_2_ at 8.13 ppm. Hydrazinolysis of compound **12** with hydrazine hydrate afforded the amino pyrazole derivative **13** confirmed by ^1^HNMR, which showed two singlets of NH and NH_2_ at δ 11.15 and 8.13 ppm. Furthermore, the reaction of norfloxacin with various reagents, such as ethyl acrylate and ethyl chloroacetate, afforded compounds **14** and **17**, respectively. ^1^HNMR of compound **14** showed the presence of an additional quartet triplet ethyl pattern at 1.61 and 3.66 ppm of ester moiety, while compound **17** appeared at 4.11 and 1.42 ppm. Moreover, the reaction of norfloxacin with o-amino thiophenol resulted in the introduction of C-3 derivative compound **15**, which showed four additional aromatic protons of the benzothiazole ring at δ 7.28–6.60 ppm in the ^1^HNMR spectrum. Finally, the acylation of compound **15** with chloroacetyl chloride formed the *N*-chloroacetyl chloride derivative **16,** which showed a singlet signal at δ 4.44 ppm for COCH_2_Cl in the ^1^HNMR spectrum (Scheme 2). Moreover, the elemental analysis and mass spectrometry for the screened compounds confirm their structures.

### 2.2. Biological Screening

#### 2.2.1. Antimicrobial Evaluation

The synthesized compounds **2**–**17** and the parent norfloxacin 1 were assessed for their in vitro antimicrobial activity against the Gram-positive strain *S. aureus ATCC 6538*, and the Gram-negative strains *P. aeruginosa ATCC 27853*, *E. coli ATCC 25922*, and *K. pneumoniae ATCC 10031* using the conventional agar cup diffusion method [22]. Table 1 compares the findings to norfloxacin and lists the minimum inhibitory concentration (MIC) results.

Table 1 demonstrates that the antibacterial screening results indicated that most of the novel compounds exhibited greater potency against the Gram-positive strain *S. aureus ATCC 6538*. The newly synthesized compounds **6**–**17** exhibit higher potency, as indicated by their minimum inhibitory concentration (MIC) values, ranging from 0.21 to 3.61 µM. In comparison, the MIC value of the parent molecule norfloxacin is 7.83 µM. Compound **6**, which is the *N*-4-piperazinyl cyano derivative of norfloxacin, demonstrated the maximum potency against *S. aureus ATCC 6538* with a MIC value of 0.21 µM which is 37 times more potent than the parent drug, norfloxacin. The novel synthesized compounds significantly affect the Gram-negative bacterium *P. aeruginosa ATCC 27853*. Compound **15**, a benzothiazole derivative of norfloxacin, exhibits the highest activity among the synthesized compounds against *P. aeruginosa ATCC 27853*. It has a MIC value of 0.20 µM, which is comparable to the parent norfloxacin (0.24 µM). In addition, other produced compounds exhibited significant efficacy against *P. aeruginosa ATCC 27853*, as indicated by their MIC values ranging from 0.32 to 4.58 µM. The final compounds, except compound **5**, demonstrated high activity against *K. pneumoniae ATCC 10031*, as evidenced by MIC values that ranged from 0.37 to 3.31 µM. Unfortunately, none of the newly synthesized compounds exhibit greater efficacy than the parent compound norfloxacin against *E. coli ATCC 25922*. However, compounds **15** and **16**, with (MIC) values of 0.40 and 0.32 µΜ, respectively, demonstrate similar potency to the parent compound norfloxacin, which has a MIC value of 0.24 μM. Notably, adding a substituent to the *C*-3-carboxyl group of the quinolone structure in compounds **15** and **16** resulted in increased potency against Gram-negative organisms, particularly *E. coli*. This finding aligns with previously reported data [28].

More importantly, compound **6** is the most potent compound against *S. aureus ATCC 6538*, and compound **7**, which has high potency against *S. aureus ATCC 6538* and significant activity against Gram-negative strains, as well as compounds **15** and **16**, which exhibited the best activity against all the tested strains, were all screened for their activity against *MRSA AUMC 261* compared with the parent compound norfloxacin. The results revealed that compound **7** exhibited more potent activity, with a MIC value of 0.80 µM, compared with the parent compound norfloxacin, which has a MIC value of 1.60 µM, as tabulated in Table 2. Also, the benzthiazolyl derivative showed moderate activity against **MRSA AUMC 261**, with a MIC value of 6.33 µM.

#### 2.2.2. Structure–Activity Relationship (SAR)

Interestingly, incorporating *N*-4-piperazinyl moieties as 2-cyanoacetyl in compound **6**, ethoxy carbonyl in compounds **7**, **14**, and **17**, corresponding hydrazide of compound **7** (compound **8**), *N*-formyl derivative **9** and its corresponding hydrazone compounds **10** and **11**, ethylidene malononitrile derivative **12** and its corresponding amino pyrazole derivative **13** enhanced the antimicrobial activity towards Gram-positive bacteria *S. aureus ATCC6538* more than towards the Gram-negative strains. The results are consistent with previously published data, demonstrating that the introduction of substitution at the *N*-4 position of piperazine moiety of fluoroquinolones mostly induces antibacterial effects against Gram-positive pathogens. Notably, adding 2-cyanoacetyl to compound **6** significantly enhances its effectiveness against *S. aureus ATCC 6538*, surpassing that of the original compound norfloxacin. Furthermore, adding an ethoxy carbonyl group to the piperazine ring in compound **7**, either directly or by using compound **14** or compound **17**, has a consistent impact on the antibacterial activity. This alteration causes a shift in activity towards the Gram-positive strain *S. aureus ATCC 6538* rather than the Gram-negative strains. Furthermore, converting ethoxy carbonyl moiety to hydrazide showed no significant change in antibacterial activity. Similarly, converting formyl derivative **10** to hydrazanomethyl derivative **11** and phenylhydrazone derivative **12** does not produce any notable change in their antibacterial activity. The introduction of benzthiazolyl moiety in compounds **15** and **16** clearly enhances activity toward the Gram-positive strain and maintains activity toward Gram-negative strains.

It is very clear from the results that the reference start norfloxacin exhibited more potent activity against Gram-negative strains with a MIC value of 0.20 µM than the Gram-positive strain with a MIC value of 7.43 µM. The results of the study showed that the biological activity of the twelve compounds **6**–**17** enhanced towards the Gram-positive strain than the reference norfloxacin because of improved lipophilicity, decreased zwitter ion character, and improved the physiochemical character in addition to the inhibition of topoisomerases enzymes resulting in the exaggerated activity.

#### 2.2.3. DNA Cleavage Assay

DNA topoisomerase II enzymes play a crucial role in the process of DNA replication and the proliferation of bacterial cells. Topoisomerase II enzymes transiently generate DNA double-strand breaks while inducing negative supercoiling of the DNA [6,7]. Fluoroquinolones act by binding DNA before breaking double-stranded DNA or binding DNA breaks, forming a triple complex (known as cleaved complex) with the topoisomerase enzymes, resulting in the stabilization of DNA breaks and the inhibition of DNA replication, hence bacterial cell death [34,35,36]. The target compounds can effectively be assessed for their ability to block DNA topoisomerases by their capacity to generate cleaved complexes from supercoiled pBR322 [7,8]. The capacity of the target compounds to induce cleaved complexes from supercoiled pBR322 was evaluated using agarose gel electrophoresis, with norfloxacin employed as the benchmark drug. The experiment conducted gel electrophoresis with and without ethidium bromide, a chemical that inserts itself between DNA strands. The purpose was to examine the impact of DNA fragmentation and the inhibition of DNA supercoil relaxing. Ethidium bromide induces positive supercoiling in closed circular DNA, enabling differentiation between relaxed DNA, nicked, and linear species. The highly potent compounds **6**, **7**, **15**, **16**, and norfloxacin were selected to evaluate their ability to poison gyrase and topoisomerase IV enzymes. Compounds **6** and **7** were chosen from a group of *N*-4-piperazinyl norfloxacin derivatives for topoisomerase II enzyme inhibition assay as compound **6** exhibited the most effectiveness against *S. aureus* among all the compounds that were synthesized. In contrast, compound **7** demonstrated the highest activity against *MRSA AUMC 261* among the compounds that were tested. Also, compounds **15** and **16** were selected for the topoisomerase II enzyme inhibition assay despite having similar activity to compounds **13**, **14**, and **17** against *S. aureus*. This choice was based on two significant factors. First, compounds **15** and **16** are derivatives of *C*-3-norfloxacin. Second, they have demonstrated strong effectiveness against Gram-positive *S. aureus* and the three tested strains of Gram-negative bacteria, as shown in Table 1. The results showed that **7** and **16** have comparable potency to the parent compound norfloxacin against DNA gyrase with IC_50_ (µM) values of 4.07, 3.57, and 2.28, respectively. More importantly, the tested compounds **6**, **7**, and **16** have a higher potency than norfloxacin against topoisomerase IV enzyme, while compound **15** has comparable potency, as shown in Table 3. The results of the topoisomerase IV inhibition assay of the tested compounds indicate their antibacterial activity against the tested strains.

#### 2.2.4. Cytotoxicity Assay

The most potent compounds as antibacterial agents **6**, **7**, **15**, and **16** in comparison to parent norfloxacin and the cytotoxic doxorubicin were tested for activity against the normal cell line WI 38 to confirm their selective activity towards bacterial strains. The results showed that the newly synthesized active compounds as antibacterial have a comparable margin of safety to the parent norfloxacin and higher selectivity than the cytotoxic doxorubicin, as shown in Table 4.

### 2.3. In Silico Studies

#### 2.3.1. Docking Study

Norfloxacin and its most effective novel analogs, **6**, **7**, **15**, and **16**, were docked into the gyrase enzyme active site (PDB: 2XCT) to investigate potential interactions resulting from the drug’s recent structural redesign [22,28]. The most effective derivative’s docked ligand poses and co-crystallized ligand’s root-mean-square deviation (RMSD) about crystal orientation were less than 1.5, demonstrating the docking method’s success in accurately predicting ligand orientation. Also, the target compounds superimposed over the co-crystalized ligand in the active site of *S. aureus* DNA gyrase (PDB: 2XCT) which confirms the validation of the docking study. The binding free energy (G) values for the docked compounds to the enzyme active site ranged from −7.84 to −10.94, with the parent norfloxacin having a value of −8.33, as shown in Table 5. Negative binding scores guarantee that norfloxacin derivatives will bind spontaneously to the active site of the DNA gyrase enzyme.

**Figure 2:**
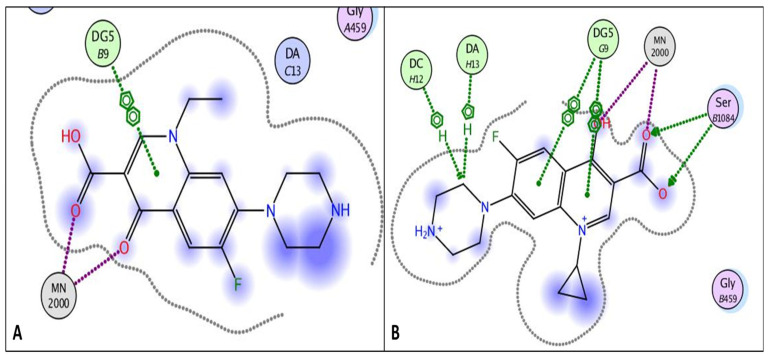
(**A**) the parent compound norfloxacin and (**B**) the co-crystallized ligand ciprofloxacin in 2D models bound to the active site of the DNA gyrase enzyme (PDB: 2XCT).

**Figure 3:**
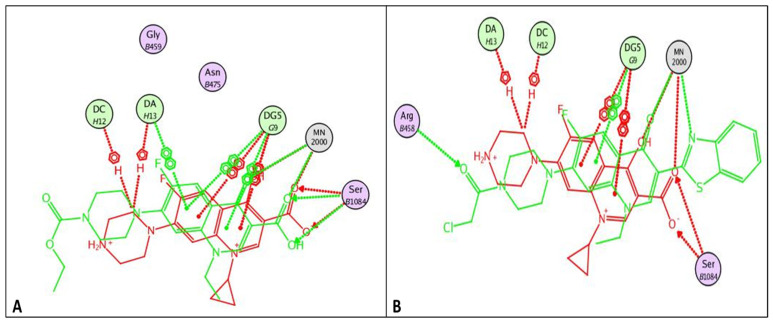
(**A**) The superimposition of the target compound **7** (green color) and co-crystallized ligand (red color, ciprofloxacin) bound to the active site of the DNA gyrase enzyme (PDB: 2XCT). (**B**) The superimposition of the target compound **16** (green color) and co-crystallized ligand (red color, ciprofloxacin) bound to the active site of the DNA gyrase enzyme (PDB: 2XCT).

**Figure 4:**
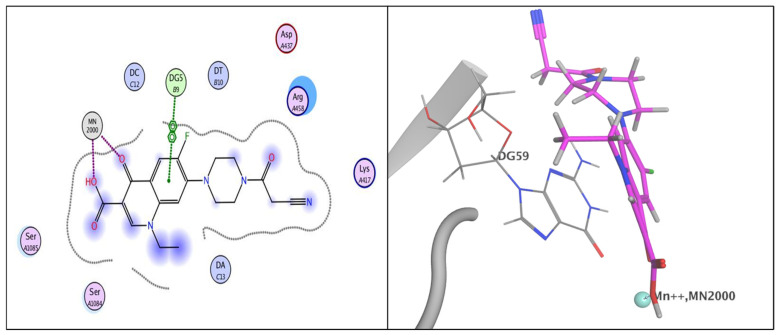
Compound **6** in 2D and 3D models bound to the active site of the DNA gyrase enzyme (PDB: 2XCT).

**Figure 5:**
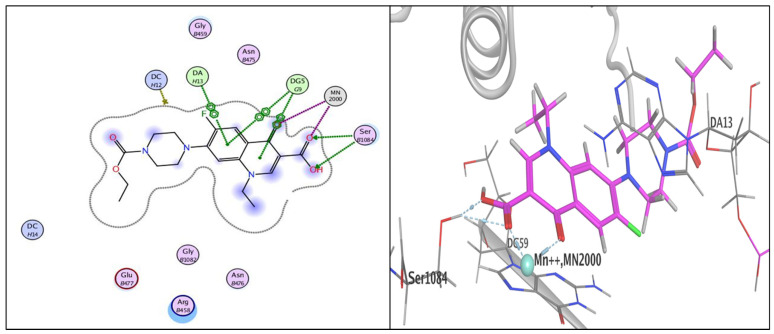
Compound **7** in 2D and 3D models bound to the active site of the DNA gyrase enzyme (PDB: 2XCT).

**Figure 6:**
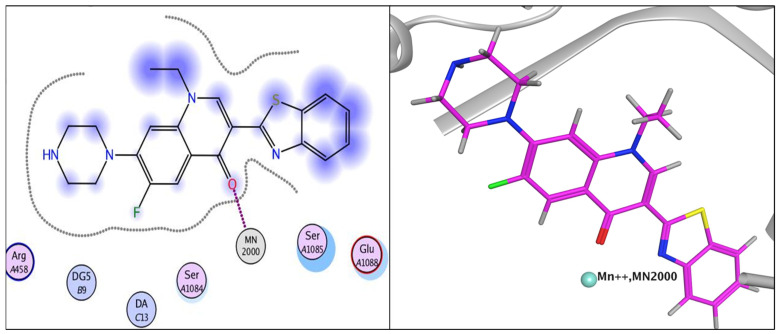
2D and 3D models of compound **15** bound to the DNA gyrase enzyme active site (PDB: 2XCT).

**Figure 7:**
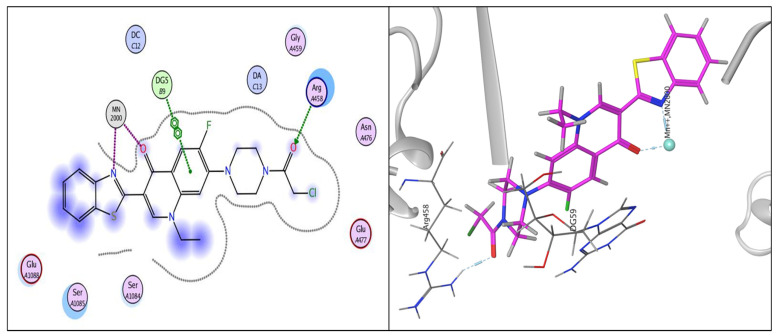
Compound **16** in 2D and 3D models bound to the active site of the DNA gyrase enzyme active site (PDB: 2XCT).

The docking analysis indicated that compounds **6**, **7**, **15**, and **16** have the potential to bind to the active site pocket of *S. aureus* DNA gyrase (PDB: 2XCT) in a manner comparable to norfloxacin. All *N*-4-piperazinyl derivatives conduct a metal interaction through the Mn atom existing in the active site via *C*-3-carboxylic or/and *C*-4-carbonyl moieties of norfloxacin and all docked compounds (Table 5 and Figure 2, Figure 3, Figure 4, Figure 5, Figure 6 and Figure 7). The introduced *N*-piperazinyl moiety resulted in extra interactions in addition to Mn metal interactions, such as hydrophobic and Vander Walls interactions, which may participate in the higher stability of the complexes that formed with the enzyme. The elevated energy scores observed for compounds **6**, **7**, and **16** can be ascribed to increased hydrogen bonding and Pi-Pi interactions. More importantly, the newly synthesized compound **16** showed extra hydrogen bonding with amino acid residue ARG 458 via the introduced *N*-4-piperazinyl moiety, Figure 7.

The introduction of substituent to *C*-3-carboxylic in compound **15** showed chelation with Mn via *C*-4-carbonyl moiety. While compound **16** with substitutions on both *N*-4 and *C*-3 chelate Mn via *C*-4-carbonyl and benzthiazolyl moieties in addition to Pi-Pi interactions with the hydrophobic group (Table 5 and Figure 6 and Figure 7). It is worth mentioning that compounds **6** and **7** the most active compounds against *S. aureus ATCC 6538* and *MRSA AUMC 261*, respectively, showed optimal binding to the active site of the gyrase enzyme (PDB: 2XCT). Figure 4 and Figure 5 explain their observed potent activity toward *S. aureus* (MIC = 0.21 μM) and *MRSA AUMC 261* (MIC = 0.80 μM), respectively, reflecting the strong correlation between docking data and biological data observed.

#### 2.3.2. Physicochemical and Pharmacokinetic Prediction

**Figure 8:**
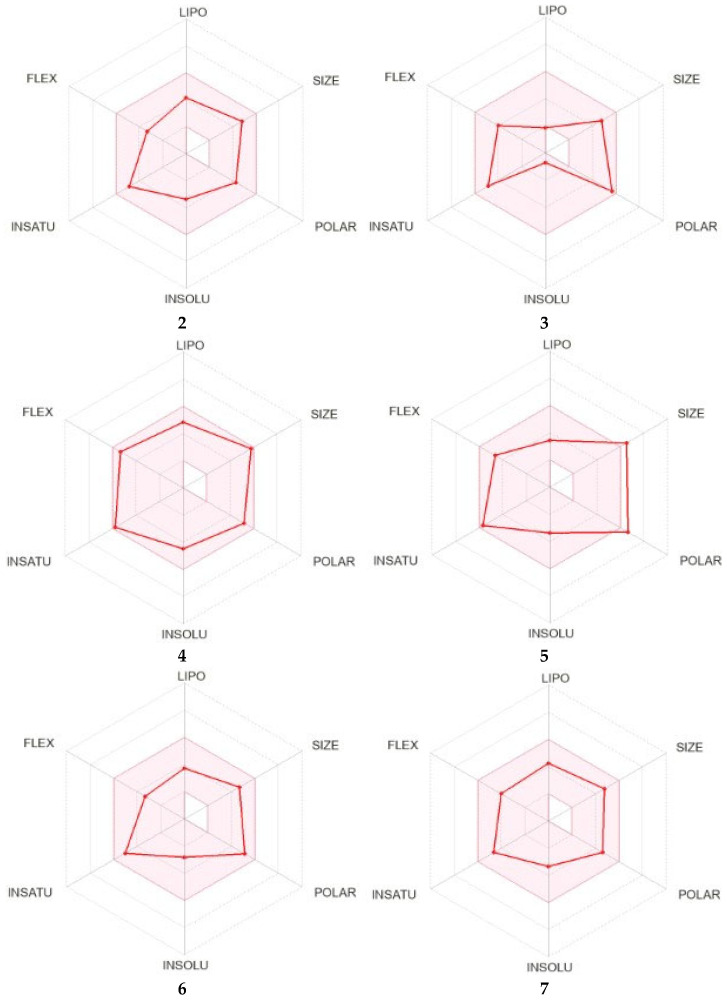

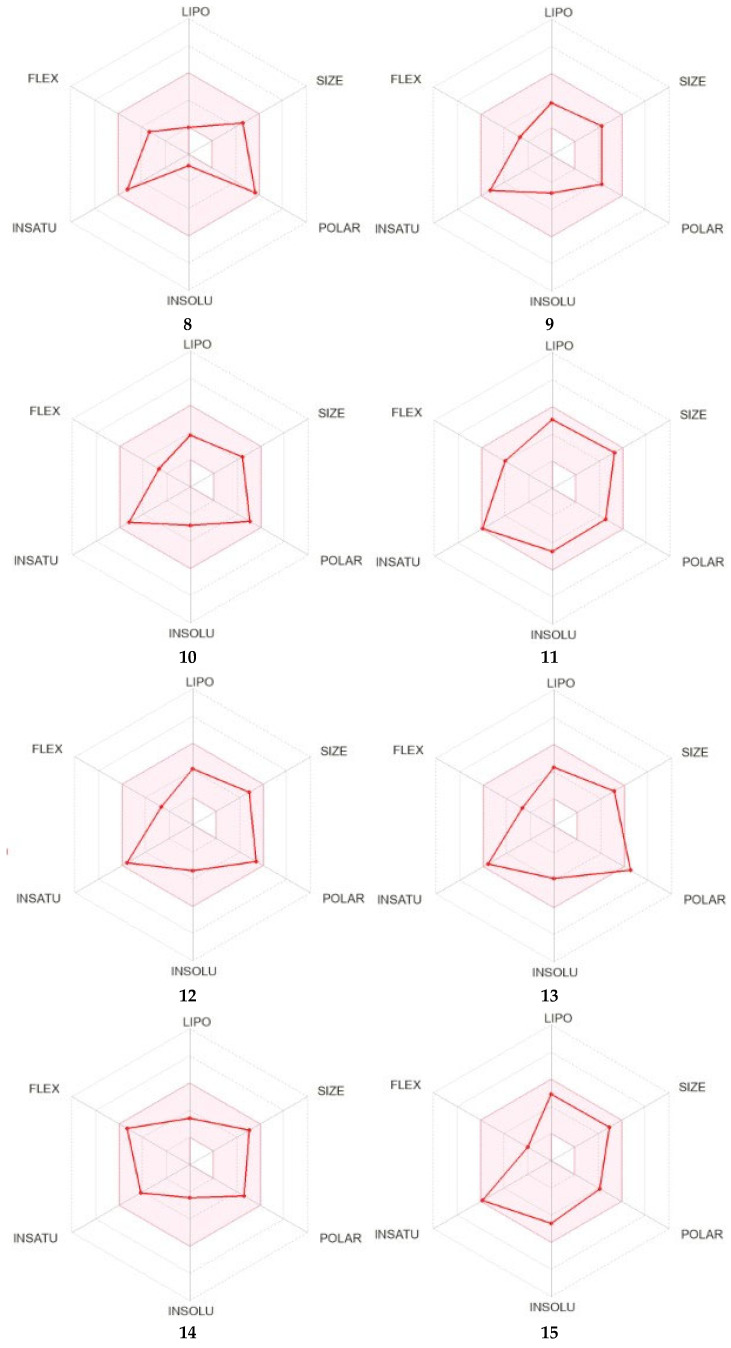

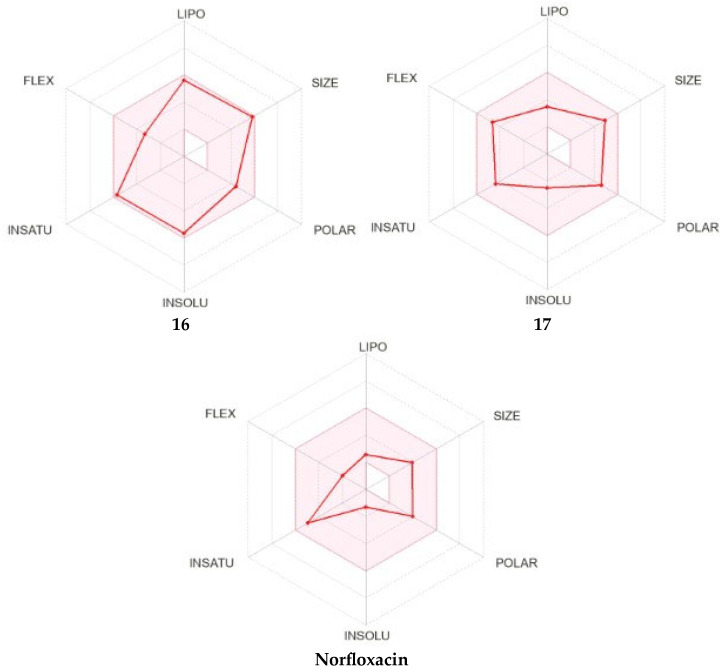
Rader model for target compounds **2**–**17** and norfloxacin.

**Figure 9:**
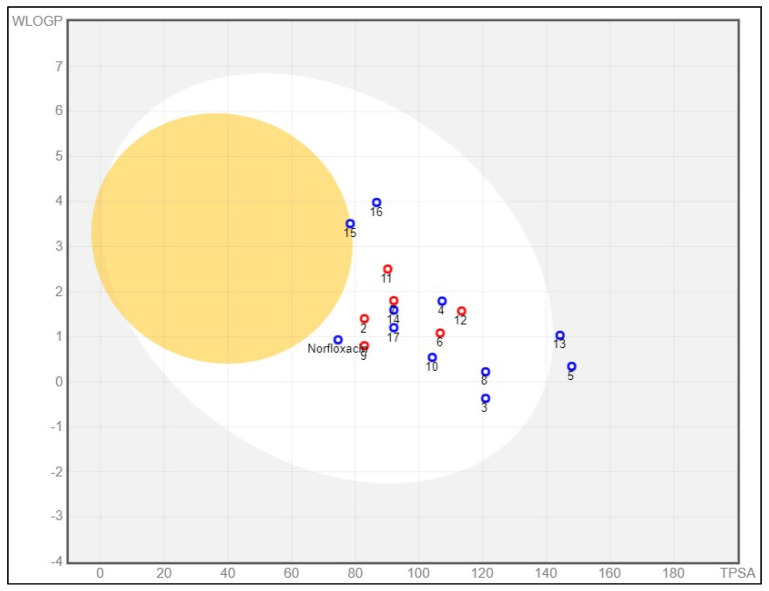
The BOILED-Egg model of target compound **2**−**17** and norfloxacin.

A potential drug candidate must exhibit a reasonable pharmacokinetic profile to reach the clinic. Consequently, the physicochemical and pharmacokinetic characteristics of the target derivatives were forecasted via Swiss ADME, as illustrated in Tables S6–S10 and Figure 8 and Figure 9. Except for compounds **5** and **13**, all target compounds were predicted to have high gastrointestinal absorption. Apart from compound **15,** all target compounds were predicted not to cause centrally adverse effects as they predicted not to pass the blood–brain barrier. Compounds **2**, **6**, **7**, **9**, **11,** and **12** were predicted to resist P-gp efflux. The expected impact of the target drugs on CYP450 enzymes, namely CYP1A2, CYP2C19, CYP2C9, CYP2D6, and CYP3A4, suggests that there is a low likelihood of drug–drug interactions occurring. Target compounds **2**, **3**, **6**, **8**, **9**, **10**, **14**, and **17** were expected not to be inhibitors for all mentioned CYP enzymes. Target compounds **5**, **11**, **12**, and **13** were predicted to be inhibitors only for CYP2C9 without any effect on other CYP enzymes [37,38].

All target compounds **2**–**17** have molecular weights below 500 g/mol, except for compound **5**, which has a weight above 545.56 g/mol. Also, compound **5** has more than ten nitrogen and oxygen atoms, representing the two Lipinski violations for this derivative with its high molecular weight, Table S10. Otherwise, all target compounds adhere to the Lipinski rule without any violations. All target compounds have no violations in the Ghose filter except compound except compounds **5** and **16**, which have two violations (MW > 480, MR > 130). All compounds have no violations in the Veber and Egan filter except compounds **5** and **13**, which have one violation (TPSA > 140 and >131.6, respectively). All compounds have no violations in the Muegge filter except compounds **3** and **8** (XLOGP3 < −2). About the Abbott bioavailability score, it was observed that all target derivatives **2**–**17** exhibit favorable oral absorption.

The bioavailability radar included six physicochemical properties: lipophilicity, size, polarity, solubility, flexibility, and saturation. The lipophilicity of all target compounds **2**–**17** is acceptable (XLOGP3 from −0.7 to +5.0). Except for compound 5, the size of all target compounds **2**–**17** ranges from 150 g/mol to 500 g/mol. Apart from compounds **5** and **13**, all target compounds **2**–**17** polarity is within a reasonable range (TPSA from 20 Å^2^ to 130 Å^2^). All target compounds **2**–**17** showed acceptable solubility (Log S (ESOL) within a range of −6 to 0). All target compounds **2**–**17** have reasonable instauration degree (Fraction Csp3 range from 0.25 to 1.0). Finally, all target compounds **2**–**17** exhibited adequate flexibility (rotatable bonds less than 9).

The BOILED-Egg approach is a reliable model that precisely predicts the permeation of substances in both the gastrointestinal tract and the brain. It achieves this by calculating specific compounds’ lipophilicity (measured in terms of WLOGP) and polarity (measured in terms of TPSA). Apart from compounds **5** and **13**, the target compounds **2**–**17** exhibited significant gastrointestinal absorption. In addition, it has been confirmed that all target compounds except compound **15** do not cross the blood–brain barrier, indicating their favorable safety profile for the central nervous system.

## 3. Experimental

### 3.1. Chemistry

All Chemicals were purchased from commercial sources and used without any further purification. Sigma-Aldrich provided the chemicals and solvents used in this study. Reactions were monitored by TLC, using Merk 9385 pre-coated aluminum plate silica gel (Kiesel gel 60) 5 cm × 20 cm plates using methylene chloride/methanol (19:1 *v*/*v*) as eluent, and spots were detected by exposure to UV lamp at ƛ = 254 nm. Melting points were determined on an electrothermal melting point apparatus (Stuart Scientific Co., Berlin, Germany) and were uncorrected. IR spectra were recorded as KBr disks on a Shimadzu 408 instrument spectrophotometer at the Faculty of Science, Sohag University. NMR spectra were taken on a Bruker AM NMR (400 MHz) spectrometer at the Faculty of Science, Sohag University. NMR data are recorded in parts per million (ppm) in reference to tetetramethylsilane (TMS) as an internal standard. Elemental microanalysis was carried out at Al-Azhar University, Egypt, using Shimadzu’s GC/MS-QP5050A instrument at the Regional Centre for Mycology and Biotechnology.

The synthesis and characterization of the synthesized target compounds **2**–**17** are described in detail in the Supplementary Data Section.

Compound **2** is prepared as reported by acylation of norfloxacin and confirmed by its melting point [39,40] and ^1^HNMR.

*Synthesis of compound **2***

A solution of norfloxacin **1** (4 mmol, 1.276 g) in DMF (10 mL) was mixed with chloroacetyl chloride (4.4 mmol, 0.497 g) in a dropwise manner while stirring for approximately 1 h. The whole mixture was agitated for an extra 2 h. Afterward, the liquid was put into ice and neutralized using dilute HCl. The product in solid form was obtained through filtration, followed by washing with water, drying, and purification through recrystallization using methanol.

*7-(4-(2-Chloroacetyl) piperazin-1-yl)-1-ethyl-6-fluoro-4-oxo-1,4-dihydroquinolin-3-carboxylic acid **2***

White powder (5.88 g, 92.8% yield); mp: 258–259 °C (reported mp:257–258) [39,40]; FT IR (KBr) ν max cm^−1^: 3420 (OH, stretching), 3056 (CH-aromatic) 2985–2836 (CH_2_, CH_3_, stretching), 1693 (C=O of carboxylic stretching), 1646 (C=O of amide, stretching) and 1628 (C=C, stretching); ^1^H-NMR (δ ppm): 15.19 (s, 1H, COOH), 8.93 (s, 1H, quinolone H-2), 7.93 (d, *J_H-F_* = 13 Hz, 1H, quinolone-H-5), 7.22 (d, *J_H-F_* = 7.6 Hz, quinolone H-8); 4.61 (q, *J_H-H_* = 7.2 Hz, 2H, -CH_2_-CH_3_), 4.30 (s, 4.20, CH_2_-Cl), 3.76 (broad, 2H, piperazine-H), 3.68 (broad, piperazine 2H), 3.41 (broad, piperazine 4H), 1.43 (t, *J_H-H_* = 7.2 Hz, 3H, -CH_2_-CH_3_).

*Synthesis of compound **3***

A solution of compound **2** (1 mmol, 0.395 g) in (10 mL) ethanol was mixed with hydrazine hydrate (2 mmol, 0.1 g) in a dropwise technique while stirring. Then, the reaction mixture underwent reflux for approximately 7 h. After that, the reaction mixture was cooled, and the resulting solid product was obtained using filtration. The recovered solid was then washed with dioxan, dried, and purified using recrystallization with acetone.

*7-(4-(Aminoglycyl)piperazin-1-yl)-1-ethyl-6-fluoro-4-oxo-1,4-dihydroquinolin-3-carboxylic acid **3**.*

Pale yellow powder (0.208 g, 53% yield); mp 280–282 °C; FT IR (KBr) ν max cm^−1^; 3421 (OH, stretching), 3220, 3120 and 3190, (NH, NH_2_), 3057 (CH-aromatic), 2986–2835 (CH_2_, CH_3_, stretching), 1693 (C=O of carboxylic, stretching), 1645 (C=O of amide, stretching) and 1629 (C=C stretching); ^1^H-NMR (δ ppm): 15.23 (s, 1H, carboxylic-H), 8.96 (s, 1H, quinolone H-2), 7.93 (d, *J_H-F_* = 13.6 Hz, 1H, quinolone-H-5), 7.22 (d, *J_H-F_* = 7.6 Hz, 1H, quinolone-H-8), 4.61 (q, *J_H-H_* = 7.2 Hz, 2H, -CH_2_-CH_3_), 4.13 (s, IH, NH), 3.80 (s, 2H, NH_2_), 2.80–2.75 (broad, 4H, piperazinyl-H), 2.75–2.68 (broad, 4H, piperazinyl-H), 2.93 (s, 2H, NHCOCH_2_), 1.43 (t, *J_H-H_* = 7.2 Hz, 3H, -CH_2_CH_3_); ^13^CNMR (DMSO-*d_6_*) δ ppm: 176.6, 166.6, 162.8, 149.6, 132.6, 119.9, 111.8 (23 Hz), 111.61, 107.0, 106.5, 49.6, 36.3, 34.6, 31.3, 14.8; Anal. Calcd for C_17_H_20_FN_5_O_4_: C, 54.11; H, 5.34; N, 18.56. Found: C, 54.31; H, 5.28; N, 18.53.

*Synthesis of compound **4***

The compound **3** (1 mmol, 0.391 g) will undergo condensation with benzaldehyde (1 mmol, 0.106 g) in the presence of piperidine in ethanol (10 mL). The reaction mixture underwent reflux for approximately 3 h. The reaction was chilled, and the resulting solid product was obtained using filtration and subsequently rinsed with ethanol.

*7-(4-((Benzylidene-amino)glycyl)piperazin-1-yl)-1-ethyl-6-fluoro-4-oxo-1,4-dihydroquinolin-3-carboxylic acid **4***

Yellow powder (0.336 g, 70% yield); mp 285–287 °C; FT IR (KBr) ν max cm^−1^: 3421 (OH, stretching), 3100 (NH), 3057 (CH-aromatic) 2986–2835 (CH_2_, CH_3_, stretching), 1693 (C=O carboxylic stretching), 1646 (C=O amide stretching) and 1628 (C=C stretching); ^1^H-NMR (δ ppm): 15.20 (s, 1H, carboxylic-H), 8.93 (s, 1H, quinolone-H-2), 7.93 (d, *J_H-F_* = 13.6 Hz, 1H, quinolone-H-5), 7.77 (s, 1H, N=CH-C_6_H_5_), 7.22 (d, *J_H-F_* = 7.6 Hz, 1H, quinolone-H-8), 7.43–7.23 (m, 5H, Ar-H), 6.72 (s, 1H, NH), 5.41 (s, 2H, CH_2_CO) 4.61 (q, *J_H-H_* = 7.2 Hz, 2H, -CH_2_-CH_3_), 3.76 (broad, 2H, piperazine-H), 3.68 (broad, quinolone H-2), 3.41 (broad, 4H, piperazine-H) 1.43 (t, *J_H-H_* = 7.2 Hz, 3H, -CH_2_CH_3_); ^13^CNMR (DMSO-*d_6_*) δ ppm: 176.6, 166.6, 162.5, 159.5, 154.5, 152.3, 149.2, 145,9, 144.7, 137.2, 111.6, 107.6, 106.5,73.7, 72.2, 50.0, 44.4, 36.6, 34.5, 31.3, 14.8; Anal. Calcd for C_25_H_26_FN_5_O_4_: C, 62.62; H, 5.47; N, 14.61. Found: C, 62.49; H, 5.44; N, 14.55.

*Synthesis of compound **5***

A solution of compound **3** (0.391 g, 1 mmol) in ethanol (10 mL) was mixed with benzylidene malononitrile (1 mmol) and stirred in the presence of a small amount of piperidine. The combination underwent reflux for 3 h. Then, the reaction mixture was cooled, and the resulting product was obtained using filtration, washed with ethanol, and dried.

*7-(4-(2-(3-amino-4-cyano-5-phenyl-2,5-dihydro-1H-pyrazol-1-yl)acetyl)piperazin-1-yl)-1-ethyl-6-fluoro-4-oxo-1,4-dihydroquinolin-3-carboxylic acid **5***

Pale yellow powder (0.420 g, 77% yield); mp: 270–272 °C; FT IR (KBr) ν max cm^−1^: 3421 (OH, st), 3340, 3230, 3120(NH_2_, NH), 3057 (CH-aromatic) 2986–2835 (CH_2_, CH_3_, stretching), 2209 (CN), 1693 (C=O carboxylic stretching), 1646 (C=O amide stretching) and 1628 (C=C stretching); ^1^H-NMR (δ ppm): 15.20 (s, 1H, COOH), 10.45 (br. IH, NH), 9.60–9.63 (m, 1H, Ar-H), 8.96 (m, 2H, quinolone H-2 + Ar-H); 7.93 (d, *J_H-F_* = 13.6 Hz, 1H, quinolone-H-5), 7.65–7.68 (m, 2H, Ar-H), 7.22–7.26 (m, 2H, Ar-H), 7.21 (d, *J_H-F_* = 7.6 Hz, 1H, quinolone-H-8), 6.66 (s, 2H, NH_2_), 4.61 (q, *J_H-H_* = 7.287 Hz, 2H, -CH_2_-CH_3_), 4.58 (s, 1H, CH-C_6_H_5_), 4.13 (s, 2H, CH_2_CO), 3.60–3.68 (broad, 4H, piperazine-H), 3.37–3.41 (broad, 4H, piperazine-H); 1.43 (t, *J_H-H_* = 7 Hz, 3H, -CH_2_CH_3_), ^13^CNMR (DMSO-*d_6_*), δ ppm: 176.6, 166.6, 162.5, 159.5, 154.5, 152.3, 149.2, 145.9, 144.7, 137.2, 119.9, 111.6, 107.6, 106.5, 73.7, 72.2, 56.5, 50.0, 44.4, 36.6, 34.5, 31.3, 29.4, 14.8; Anal. Calcd for C_19_H_19_FN_4_O_4_: C, 59.06; H, 4.96; N, 14.50. Found: C, 58.90; H, 5.02; N, 14.38.

*Synthesis of compound **6***

A mixture of compound **2** (1 mmol, 0.396 g) in 10 mL ethanol and KCN (1 mmol, 0.065 g) was mixed with stirring. The mixture underwent reflux for 4 h. Then, the reaction mixture was cooled, and the resultant solid product was obtained via filtration, followed by washing, crystallized from ethanol, and subsequently dried.

*7-(4-(2-Cyanoacetyl) piperazin-1-yl)-1-ethyl-4-oxo-1,4-dihydroquinolin-3-carboxylic acid **6***

White powder (0.331 g, 90% yield); mp: 246–247 °C, reported mp: 245–250 °C [38,39]; FT IR (KBr) ν max cm^−1^: 3421 (OH, stretching), 3057 (CH-aromatic) 2986–2833 (CH_2_, CH_3_, stretching), 1690 (C=O carboxylic stretching), 2220 (CN), 1646 (C=O amide stretching) and 1628 (C=C, st); ^1^H-NMR (δ ppm): 15.20 (s, 1H, COOH), 8.90 (s, 1H, quinolone H-2), 7.83 (d, *J_H-F_* = 13.6 Hz, 1H, quinolone-H-5), 7.16 (d, *J_H-F_* = 7.6 Hz, 1H, quinolone-H-8), 4.57 (q, *J_H-H_* = 7.2 Hz, 2H, -CH_2_-CH_3_), 4.07 (s, 2H, CNCH_2_CO), 3.70 (broad, 2H, piperazinyl-H), 3.60 (broad, piperazine 2H), 3.24–3.30 (broad, 4H, piperazinyl-H), 1.42 (t, *J_H-H_* = 7.2 Hz, 3H, -CH_3_); ^13^CNMR (DMSO-*d_6_*), δ ppm: 177.2, 166.5, 149.3, 145.7, 137.6, 119.7, 116.8, 111.2, 107.6, 106.4, 104.1, 49.6, 49.4, 45.5, 42.3, 25.7, 15.1, 12.3; Anal. Calcd for C_19_H_19_FN_4_O_4_: C, 59.06; H, 4.96; N, 14.50. Found: C, 58.96; H, 5.02; N, 14.48; MS (APCl) calcd for C_19_H_19_FN_4_O_4_ [M+H]^+^: 387.15, found: 387.20.

*Synthesis of compound **7***

A solution of compound **1** (1 mmol, 0.319 g) in acetone (10 mL) and ethyl chloroformate (1 mmol, 0.108 g) in the presence of 0.25 gm of sodium acetate was mixed with stirring. Then, the reaction mixture underwent reflux for approximately 5 h. The whole mixture was poured into ice. The product in solid form was obtained by filtration, followed by washing with acetone and subsequent drying.

*7-(4-(Ethoxy carbonyl) piperazin-1-yl)-1-ethyl-6-fluoro-4-oxo-1,4-dihydroquinolin-3-carboxylic acid **7***

White powder (0.235 g, 60% yield); mp: 272–273 °C; FT IR (KBr) ν max cm^−1^: 3421 (OH, stretching), 3387, 3057 (CH-Ar), 2986–2835 (CH_2_, CH_3_, stretching), 1693 (C=O carboxylic stretching), and 1628 (C=C, stretching); ^1^H-NMR (δ ppm): 15.20 (s, 1H, carboxylic-H), 8.93 (s, 1H, quinolone H-2), 7.93 (d, *J_H-F_* = 13.6 Hz, 1H, quinolone-H-5), 7.22 (d, *J_H-F_* = 7.6 Hz, 1H, quinolone-H-8), 4.58 (q, *J_H-H_* = 7.2 Hz, 2H, -NCH_2_-CH_3_), 4.09 (q, *J_H-H_* = 7 Hz, 2H, -OCH_2_-CH_3_), 3.59–3.66 (broad, 4H, piperazine-H), 3.43–3.49 (broad, 4H, piperazine-H), 1.42 (t, *J_H-H_* = 7.2 Hz, 2H, -NCH_2_CH_3_), 1.22 (t, *J_H-H_* = 7 Hz, 2H, -OCH_2_CH_3_), ^13^CNMR (DMSO-d_6_), δ ppm: 176.9, 167.2, 155.1 (244 Hz), 154.7, 152.2, 149.2, 138.4, 120.6, 111.8, 108.3, 106.6, 61.4, 56.7, 50.1, 43.5, 19.1, 15.0; Anal. Calcd for C_19_H_22_FN_5_O_5_: C, 58.31; H, 5.67; N, 10.74. Found: C, 58.09; H, 5.54; N, 10.59; MS (APCl) calcd for C_19_H_22_FN_3_O_5_: 391.15, found [M+Na]^+^: 414.70.

*Synthesis of compound 8*

A mixture of compound **7** (1 mmol, 0.391 g) in methanol (10 mL) and 1 mL of hydrazine underwent reflux for 4 h with stirring. The reaction mixture was cooled and the resultant solid product was obtained using filtration, followed by washing with ethanol and subsequent drying.

*1-Ethyl-6-fluoro-7-(4-(hydrazinecarbonyl)piperazin-1-yl)-4-oxo-1,4-dihydroquinolin-3-carboxylic acid **8***

Pale yellow powder (0.245 g, 73% yield); mp 280–282 °C; FT IR (KBr) ν max cm^−1^: 3445 (OH, stretching), 3425, 3320 and 3150 (NH, NH_2_), (CH-Ar) 2986–2835 (CH_2_, CH_3_, stretching), 1740 (C=O carboxylic stretching), 1628 and (C=O amide stretching); ^1^H-NMR (δ ppm): 15.26 (s, 1H, carboxylic-H), 8.93 (s, 1H, quinolone H-2), 8.50 (s, 1H, NH), 7.95 (d, *J_H-F_* = 13.6 Hz, 1H, quinolone-H-5), 7.21 (d, *J_H-F_* = 7.6 Hz, 1H, quinolone-H-8), 4.59 (q, *J_H-H_* = 7.2 Hz, 2H, -CH_2_-CH_3_), 4.08 (br, 2H, NH_2_), 3.68–3.57 (br, 4H, piperazine-H), 3.36–343 (broad, 4H, piperazine-H), 1.22 (t, *J_H-H_* = 7.2 Hz, 3H, -CH_3_); ^13^CNMR (DMSO-*d_6_*), δ ppm: 176.2, 160.8, 155.5, 148.8, 146.2, 137,8, 119.9, 112.2, 107.4, 107.0, 76.1, 61.3, 49.3, 43.9, 14.9; Anal. Calcd for C_17_H_20_FN_5_O_4_: C, 54.11; H, 5.22; N, 12.10. Found: C, 53.89; H, 5.34; N, 12.19.

*Synthesis of compound **9***

A solution of compound **1** (1 mmol, 0.319 g) was mixed with 0.25 mL of formic acid. Then, the whole mixture was refluxed for 2 h to melt and solidify. After cooling the reaction mixture, the solid output was triturated in cold water, filtered, washed, and dried.

*1-Ethyl-6-fluoro-7-(4-formylpiperazin-1-yl)-4-oxo-1,4-dihydroquinolin-3-carboxylic acid **9***

White powder (0.194 g, 56% yield); mp: 289–291 °C, reported mp: 292–293 °C [41]. ^1^H-NMR (δ ppm): 15.20 (s, 1H, carboxylic-H), 8.98 (br. IH, CHO), 8.14 (s, 1H, quinolone H-2), 7.92 (d, *J_H-F_* = 13.6 Hz, 1H, quinolone-H-5), 7.21 (d, *J_H-F_* = 7.6 Hz, 1H, quinolone-H-8), 4.59 (q, *J_H-H_* = 7.2 Hz, 2H, -CH_2_-CH_3_), 3.63–3.56 (broad, 4H, piperazine-H), 3.40–3.48 (broad, 4H, piperazine-H), 1.43 (t, *J_H-H_* = 7.2 Hz, 2H, -CH_2_-CH_3_).

*Synthesis of compound **10***

A solution of compound **9** (0.347 g, 1 mmol) in ethanol (10 mL) and 1 mL of hydrazine was mixed with stirring. The reaction mixture was refluxed for about 5 h. The reaction mixture was cooled, and the solid product was collected by filtration, washed with ethanol, and dried.

*1-Ethyl-6-fluoro-7-(4-(hydrazonomethyl)piperazin-1-yl)-4-oxo-1,4-dihydroquinolin-3-carboxylic acid **10***

White powder (0.208 g, **58**% yield); mp 294–296 °C; FT IR (KBr) ν max cm^−1^: 3421 (OH, stretching), 3057 (CH-Ar) 2986–2833 (CH_2_, CH_3_, stretching), 1693 (C=O carboxylic stretching), 1646 (C=O amide stretching) and 1628 (C=C, stretching); ^1^H-NMR (δ ppm): 15.20 (s, 1H, carboxylic-H), 9.62 (s, 1H, quinolone H-2), 8.91 (s, 1H, N-CH=N), 8.17 (d, *J_H-F_* = 13 Hz, 1H, quinolone-H-5), 7.92 (d, *J_H-F_* = 7.5 Hz, 1H, quinolone-H-8), 7.24 (br, 2H, NH_2_), 4.59 (q, *J_H-H_* = 7 Hz, 2H, -CH_2_-CH_3_), 3.58–3.64 (broad, 4H, piperazine), 3.44–351 (broad, 4H, piperazine-H),1.43 (t, *J_H-H_* = 7 Hz, 2H, -CH_2_-CH_3_); ^13^CNMR (DMSO-*d_6_*), δ ppm: 168.7, 167.2, 149.0, 144.1, 119.1, 90.2,78.5, 77.6, 57.2, 53.2, 50.1, 45.4, 22.8, 19.1, 14.6; Anal. Calcd for C_17_H_20_FN_5_O_3_: C, 56.50; H, 5.58; N, 19.38. Found: C, 56.39; H, 5.42; N, 19.27.

*Synthesis of compound **11***

A solution of compound **9** (1 mmol, 0.347 g) in ethanol (10 mL) was mixed with 0.12 mL of phenyl hydrazine under stirring. The whole reaction mixture was refluxed for 5 h. The reaction was cooled, and the solid product was collected by filtration, then washed with ethanol and dried.

*1-Ethyl-6-fluoro-4-oxo-7-(4-((2-phenylhydrazono)methyl)piperazin-1-yl)-1,4-dihydro-quinolin-3-carboxylic acid **11***

White Powder (0.263 g, 60% yield); mp 270–272 °C; FT IR (KBr) ν max cm^−1^: 3418 (OH, st), 3220 (NH), 3057 (CH-Ar) 2986–2835 (CH_2_, CH_3_, stretching), 1693 (C=O carboxylic stretching), 1646 (C=O amide stretching) and 1628 (C=C stretching); ^1^H-NMR (δ ppm): 15.21 (s, 1H, COOH), 8.95–8.97 (m, 2H, quinolone H-2+Ar-H), 8.55–8.57 (m, 1H, Ar-H), 8.12–8.14 (m, 2H, N=CH-NH +Ar-H), 7.92–7.94 (m, 2H, quinolone-H-5+Ar-H), 7.21 (m, 2H, quinolone-H-8+Ar-H), 4.59 (q, *J_H-H_* = 7 Hz, 2H, -NCH_2_-CH_3_), 3.76 (broad, 2H, piperazine-H), 3.68 (broad, 2H, piperazinyl-H), 3.41 (broad, 4H, piperazine-H), 1.41 (t, *J_H-H_* = 7 Hz, 3H, -NCH_2_CH_3_); ^13^CNMR (DMSO-*d_6_*) δ ppm: 177.2, 161.6, 155.5 (d, *J* = 248 Hz), 155.2, 152.3, 149.8, 145.7, 137.2, 119.7, 112.5, 111.3, 107.6, 106.9, 56.5, 50.7, 49.6, 44.7, 18.7, 15.3; Anal. Calcd for C_23_H_24_FN_5_O_3_: C, 63.15; H, 5.53; N, 16.01. Found: C, 62.90; H, 5.49; N, 15.98.

*Synthesis of compound **12***

To a solution of compound **9** (1 mmol, 0.347 g) in ethanol (10 mL), 0.07 gm of malononitrile was added with stirring in the presence of one drop of piperidine. The mixture was then subjected to the reaction. The reaction mixture was allowed to reflux for five hours. The reaction was cooled, and the solid product was collected by filtration, washed with ethanol, and dried.

*7-(4-(2,2-Dicyanovinyl)piperazin-1-yl)-1-ethyl-6-fluoro-4-oxo-1,4-dihydroquinolin-3-carboxylic acid **12***

White powder (0.304 g, 77% yield); mp 290–292 °C; FT IR (KBr) ν max cm^−1^: 3420 (OH, st), 3057 (CH-Ar) 2986–2835 (CH_2_, CH_3_, st), 2217, 2198 (2CN), 1693 (C=O carboxylic stretching), 1646 (C=O amide, stretching) and 1628 (C=C, stretching); ^1^H-NMR (δ ppm) 15.10 (s, 1H, carboxylic-H), 8.94 (s, 1H, quinolone H-2), 8.13 (s, 1H, NCH=C), 7.92 (d, *J_H-F_* = 13.6 Hz, 1H, quinolone-H-5), 7.23 (d, *J_H-F_* = 7.6 Hz, 1H, quinolone-H-8), 4.57 (q, *J_H-H_* = 7.2 Hz, 2H, -CH_2_-CH_3_), 3.52–3.62 (broad, 4H, piperazine-H), 3.32–3.40 (broad, 4H, piperazine-H), 1.43 (t, *J_H-H_* = 7.2 Hz, 3H, -CH_2_CH_3_); ^13^CNMR (DMSO-*d_6_*) δ ppm: 176.9, 166.3, 161.8, 154. 4, 152.7, 149.3, 146.0, 138.3, 134.1, 120.5, 112.0, 107.8, 107.5, 65.9, 50.8, 49.9, 45.2, 14.9; Anal. Calcd for C_20_H_18_FN_5_O_3_: C, 60.75; H, 4.59; N, 17.71. Found: C, 60.49; H, 4.43; N, 17.58.

*Synthesis of compound **13***

A solution of compound **12** (1 mmol, 0.395 g) in ethanol (10 mL) and 0.11 mL of hydrazine was prepared. One drop of piperidine was added to the solution, and the entire combination was heated under reflux for 4 h. Subsequently, the reaction mixture was subjected to cooling, and the resultant solid product was obtained using filtration, followed by washing with ethanol and subsequent drying.

*7-(4-(3-Amino-4-cyano-1H-pyrazol-5-yl) piperazin-1-yl)-1-ethyl-6-fluoro-4-oxo-1,4-dihydroquin-oline-3-carboxylic acid **13***

White powder (0.280 g, 66% yield); mp: 270–272 °C; FT IR (KBr) ν max cm^−1^: 3421 (OH, stretching), 3414, 3323, 3087 (NH, NH_2_), 3057 (CH-Ar); 2986–2835 (CH_2_, CH_3_, stretching), 2209 (CN), 1705 (C=O carboxylic stretching), 1675 (C=O amide, stretching) and 1627 (C=C, stretching); ^1^H-NMR (δ ppm): 15.20 (s, 1H, carboxylic-H), 11.15 (br. IH, NH), 8.93 (s, 1H, quinolone H-2), 8.13 (d, *J_H-F_* = 13 Hz, 1H, quinolone-H-5), 7.93 (d, *J_H-F_* = 7.5 Hz, 1H, quinolone-H-8), 7.22 (s, 2H, NH_2_), 4.58 (q, *J_H-H_* = 7 Hz, 2H, -CH_2_-CH_3_), 3.60–3.70 (broad, 4H, piperazine-H), 3.35–3.42 (broad, 4H, piperazine-H), 1.43 (t, *J_H-H_* = 7 Hz, 3H, -CH_2_CH_3_); ^13^CNMR (DMSO-*d_6_*) δ ppm: 176.7, 166.4, 161.7, 154.6, 152.3, 149.2, 145.3, 137,4, 120.3, 111.6, 107.9, 106.8, 57.2, 50.8, 50.0, 45.3, 18.9, 14.6; Anal. Calcd for C_20_H_20_FN_7_O_3_: C, 56.47; H, 4.74; N, 23.05. Found: C, 56.41; H, 4.69; N, 23.17.

*Synthesis of compound **14***

A solution of compound **1** (1 mmol, 0.319 g) in ethanol (10 mL) and 0.2 mL of ethyl acrylate was prepared. The mixture underwent reflux for 4 h. The reaction was cooled, and the resulting solid product was pulverized with water. The solid product was then obtained through filtration, rinsed with ethanol, and dried.

*7-(4-(3-Ethoxy-3-oxopropyl)piperazin-1-yl)-1-ethyl-6-fluoro-4-oxo-1,4-dihydro-quinolin-3-carboxylic acid **14***

White powder (0.323 g, 77% yield); mp 200–202 °C; FT IR (KBr) ν max cm^−1^: 3425 (OH, st), 3057 (CH-Ar), 2986–2835 (CH_2_, CH_3_, st), 1740 (C=O ester), 1628 (C=O carboxylic), 1646 (C=O amide, st) and 1628 (C=C, st); ^1^H-NMR (δ ppm): 15.35 (s, 1H, carboxylic-H), 8.98 (s, 1H, quinolone H-2), 7.85 (d, *J_H-F_* = 13.6 Hz, 1H, quinolone-H-5), 7.15 (d, *J_H-F_* = 7.6 Hz, 1H, quinolone-H-8), 4.57 (q, *J_H-H_* = 7 Hz, 2H, -CH_2_ ester), 3.61 (q, *J_H-H_* = 7 Hz, 2H, COOCH_2_CH_3_), 3.35–3.40 (broad, 4H, piperazine-H), 3.25–3.30 (broad, 4H, piperazine-H), 2.75 (t, *J_H-H_* = 7.2 Hz, 3H, -NCH_2_CH_2_), 2.59 (t, *J_H-H_* = 7.2 Hz, 3H, -NCH_2_CH_2_), 1.66 (t, *J_H-H_* = 7 Hz, 3H, -COOCH_2_CH_3_), 1.42 (t, *J_H-H_* = 7.2 Hz, 3H, -NCH_2_CH_3_); ^13^CNMR (DMSO-*d_6_*) δ ppm: 186.5, 182.6, 173.5, 167.6, 164.0, 152.0, 148.9, 119.1, 112.1, 106.5, 89.8, 74.2, 53.2, 52.6, 52.0, 50.2, 49.9, 19.8, 14.3; Anal. Calcd for C_21_H_26_FN_4_O_5_: C, 60.13; H, 6.25; N, 13.22. Found: C, 60.38; H, 6.17; N, 13.29.

*Synthesis of compound **15***

To a solution of compound **1** (1 mmol, 0.319 gm) in ethanol (5 mL) and 0.2 mL of 2-aminothiophenol in the presence of 0.2 mL of conc. H_2_SO_4_, the reaction mixture, was refluxed for about 3 h. The reaction was cooled, and the solid product underwent trituration with water, followed by filtering to collect the final solid product. The collected solid was then washed with water and subsequently dried.

*3-(Benzo[d]thiazol-2-yl)-1-ethyl-6-fluoro-7-(piperazin-1-yl)quinolin-4(1H)-one **15***

White powder (0.284 g, 70% yield); mp 293–294 °C, reported 290–295 °C [42]; ^1^H-NMR (δ ppm): 15.35 (s, 1H, carboxylic-H), 8.98 (s, 1H, quinolone H-2), 8.91 (br. IH, NH), 7.93 (d, *J_H-F_* = 13 Hz, 1H, H-5 of quinolone), 7.22 (d, *J_H-F_* = 7.6 Hz, 1H, quinolone-H-8), 7.28–6.59 (m, 4H, Ar-H), 4.61 (q, *J_H-H_* = 7 Hz, 2H, -CH_2_-CH_3_), 3.80–3.70 (broad, 4H, piperazinyl-H), 3.52 3.40 (broad, 4H, piperazinyl-H), 1.42 (t, *J_H-H_* = 7 Hz, 3H, -NCH_2_CH_3_); MS (APCl) calcd for C_22_H_21_FN_4_OS: 408.14, found [M+Na]^+^: 431.00.

*Synthesis of compound **16***

To a solution of compound **15** (1 mmol, 0.408 g) in DMF (10 mL) and 0.9 mL of triethyl amine. The reaction mixture was stirred for 30 min., and 0.5 mL of chloroacetyl chloride drop by drop. The reaction mixture was stirred at room temperature for 3 h. The reaction was poured into ice-cold water, and the solid product was collected by filtration, washed with water, and dried.

*3-(Benzo[d]thiazol-2-yl)-7-(4-(2-chloroacetyl)piperazin-1-yl)-1-ethyl-6-fluoroquinolin-4(1H)-one **16***

White powder (0.420 g, 70% yield); mp 280–202 °C; FT IR (KBr) ν max cm^−1^: 3057 (CH-Ar), 2986–2835 (CH_2_, CH_3_, stretching), 1719 (C=O carboxylic stretching), 1633 (C=O amide, stretching) and 1628 (C=C, stretching); ^1^H-NMR (δ ppm): 15.25 (s, 1H, carboxylic-H), 9.19 (s, 1H, Ar-H), 8.85 (s, 1H, quinolone H-2), 8.32–8.34 (m, 2H, Ar-H), 7.95 (d, *J_H-F_* = 13.6 Hz, 1H, quinolone-H-5), 7.20 (d, *J_H-F_* = 7.6 Hz, 1H, quinolone-H-8), 7.13 (m, 1H, Ar-H), 4.55 (q, *J_H-H_* = 7.2 Hz, 2H, -CH_2_-CH_3_), 4.44 (s, 2H, COCH_2_Cl), 3.69–3.54 (broad, 4H, piperazinyl-H), 3.40–3.31 (broad, 4H, piperazinyl-H), 1.42 (t, *J_H-H_* = 7.2 Hz, 3H, -NCH_2_CH_3_); ^13^CNMR (DMSO-*d_6_*) δ ppm: 177.4, 166.0, 154.4, 152.3, 149.0, 147.2, 146.9, 144.7, 137,2, 120.4, 119.4, 111.9, 107.6, 106.9, 61.8, 49.3, 47.4, 43.2, 15.6, 14.9; Anal. Calcd for C_24_H_22_ClFN_4_O_5_: C, 59.44; H, 4.57; N, 11.55. Found: C, 59.29; H, 4.51; N, 11.49; MS (APCl) calcd for C_24_H_22_ClFN_4_O_2_S: 484.11, found [M+H]^+^: 484.90.

*Synthesis of compound **17***

To a solution of compound 1 (1 mmol, 0.319 g) in acetone (20 mL), 0.7 mL of ethyl chloroacetate was added in the presence of 2 gm of anhydrous K2CO3. The reaction mixture was refluxed for 4 h. The reaction mixture was cooled, and the solid product was poured in ice-cold water. Then, the solid product was collected by filtration, washed with water, and dried.

*7-(4-(2-Ethoxy-2-oxoethyl) piperazin-1-yl)-1-ethyl-6-fluoro-4-oxo-1,4-dihydroquinolin-3-carboxylic acid **17***

Gray powder (0.264 g, 65% yield); mp. 233–235 °C, reported 229–231 °C [43]; ^1^H-NMR (δ ppm) 15.35 (s, 1H, carboxylic-H), 8.95 (s, 1H, quinolone H-2), 7.91 (d, *J_H-F_* = 13 Hz, 1H, quinolone-H-5), 7.18 (d, *J_H-F_* = 7.6 Hz, 1H, quinolone-H-8), 4.59 (q, *J_H-H_* = 7.2 Hz, 2H, -NCH_2_-CH_3_), 4.11 (q, *J_H-H_* = 7 Hz, 2H, -OCH_2_-CH_3_), 3.61–3.31 (broad, 8H, piperazine-H), 2.76 (s, 2H, COCH_2_), 1.42 (t, *J_H-H_* = 7 Hz,3H, OCH_2_CH_3_), 1.22 (t, *J_H-H_* = 7.2 Hz, 3H, -NCH_2_CH_3_).

### 3.2. Biological Evaluations

#### 3.2.1. Screening of Antibacterial Activity

##### Microbial Strains and Culture Conditions

The antibacterial activity of the samples was evaluated at the Assiut University Mycological Centre (AUMC), Faculty of Science, using the standard agar cup diffusion method [22]. The strains used in this investigation were *Pseudomonas aeruginosa ATCC 27853*, *K. pneumoniae ATCC 10031*, *E. coli ATCC 25922* (all Gram-negative), and *S. aureus ATCC 6538* (Gram-positive). These strains were provided by the microbiological resource center at the Faculty of Agriculture, Ain Shams University, Cairo, Egypt. All isolates were maintained at −70 °C in Trypticase Soya Broth (10% glycerol, Becton and Dickinson). Prior to injection, all isolates were subcultured for 24 h at 37 °C on Trypticase Soya Agar (TSA, Becton and Dickinson) and TSB.

##### Determination of the Minimum Inhibitory Concentration (MIC)

The examined bacteria were then plated using Mueller Hinton agar media (Oxoid, 20 mL) and 1 108 CFU/mL (0.5 McFarland turbidity) on sterile Petri dishes. After being carefully flipped to make sure that the microorganisms were dispersed evenly, the plates were set aside to solidify on a level surface. After the material had solidified, four equally spaced circular wells with a diameter of 10 mm were painstakingly bored using a sterile cork bore. All chemicals under investigation underwent two-fold successive dilutions in order. Each dilution was added to each well three times in 100 μL increments using a micropipette. All plates underwent 24 h incubation period at 37 °C. Following measurement, the inhibition zones average was calculated. To find the MICs, Finally, the minimum inhibitory concentration (MIC) was defined as the lowest concentration that inhibits the growth of each strain, all tests were carried out in triplicates [19].

#### 3.2.2. Topoisomerase II Inhibition Assays

##### *S. aureus* Gyrase Supercoiling Assay

The *S. aureus* DNA gyrase assay for compounds **6** and 7, with a high potency against Gram-positive strain, and compounds **15** and **16,** with a high potency against Gram-negative strains in addition to the parent norfloxacin, was conducted by the suggested techniques collected from the literature [44]. In the three separate replicate runs, the novel tested compounds were dissolved in DMSO, serially diluted at doses of 100, 10, 1, and 0.1 M, and then tested in reaction mixtures. In a total solution containing 40 mM HEPES, 10 mM magnesium acetate, 500 mM potassium glutamate, 2 mM ATP, 0.05 mg/mL albumin, and Relaxed pBR322. At 37 °C, the DNA gyrase from *S. aureus* was incubated for 30 to 60 min. *Staph. aureus* gyrase DNA gyrase supercoiling reactions were stopped by adding 30 L of STEB and 30 L of chloroform/isoamyl alcohol (*v*:*v*, 24:1), centrifuged for one minute, and then loaded 20 L of this on a 1% agarose gel and run at 75 V for roughly 2 h. The gel was stained by ethidium bromide in water (0.5 mg/L). The fluorescent images were recorded using a UV transilluminator imaging device at a wavelength of 300 nm. The fluorescence intensity of the supercoiled plasmid reaction result was quantitated using the Imag Quant programmed (Molecular Dynamics). By applying nonlinear regression analysis in Graph Pad Prism, the results as IC_50_ values (concentration of the tested substance that results in 50% inhibition of enzyme activity) were calculated [22].

##### *S. aureus* Topoisomerase IV Decatenation Assay

The Topo IV decatenation of the most potent newly synthesized derivatives as antibacterial agents (compounds **6**, **7**, **15**, and **16**) in addition to the parent norfloxacin was evaluated, and IC_50_ values were established as a previously reported technique [44]. The following substances were utilized in this test: Topo IV Assay Buffer (provided as 5X), 50 mM Tris-HCl (pH 7.5), 5 mM magnesium chloride, 350 mM potassium glutamate, 5 mM DTT, and 1.5 m MATP. Store at or below -200C. 50 mM Tris-HCl (pH 7.5), 1 mM EDTA, 1 mM DTT, and 40% (*v*/*v*) glycerol (provided as 1X) make up the dilution buffer. The *S. aureus* Topo IV enzyme kDNA (100 ng/L) served as the substrate. STEB: *S. aureus* Topo IV, 40 percent sucrose (*w*/*v*), kDNA, 10 mM EDTA briefly, 100 mM Tris-HCl Bromophenol Blue, 0.5 mg/mL, pH 8, and various concentrations of the investigated substances were combined. For 30 min, the mixtures were incubated at 37 °C. STEB was used to halt the responses. Agarose gel electrophoresis was used to analyze the effects of the process. The gel was then colored with ethidium bromide and photographed under UV light [19].

#### 3.2.3. Cytotoxicity Assay

The cytotoxicity of the most potent compounds screened against the normal cell line WI 38 by MTT assay method using The American Type Culture Collection provided the cell line cells [45], which were grown in DMEM (Invitrogen/Life Technologies, Carlsbad, CA, USA) with 10% FBS (Hyclone, Logan, UT, USA), 10 ug/mL of insulin (Sigma, Cream Ridge, NJ, USA), and 1% penicillin–streptomycin. The remaining substances and tools were from either Sigma or Invitrogen. Before performing the MTT experiment, cells were seeded on a 96-well plate at a density of 1.2–1.8 10,000 cells/well in a volume of 100 μL complete growth media + 100 μL of the test substance each well. Multiwell plates are an excellent choice for using the MTT method, which is used to track in vitro cytotoxicity. Using cells in the log phase of growth and keeping the ultimate cell density around 106 cells/cm^2^ will produce the best results. Every test should include a blank with a full medium but no cells. Cultures should be moved from the incubator into a sterile workspace, such as a laminar flow hood. Each MTT [M-5655] vial should be reconstituted with 3 mL of medium or balanced salt solution devoid of phenol red and serum. 10% of the volume of the culture medium should be added to the reconstituted MTT solution. Incubate cultures again for 2–4 h, depending on the type of cell and the maximal cell density. (A 2-h incubation period is usually sufficient, although it can be extended for cells with low densities or low metabolic activity.) When comparing incubation periods, they should be comparable. After the incubation period, remove the cultures from the incubator and apply MTT Solubilization Solution [M-8910] in a quantity equal to the volume of the original culture media to dissolve the formazan crystals that formed. In a gyratory shaker, gentle blending will improve solubility. Trituration, or pipetting up and down, may occasionally be necessary to completely dissolve the MTT formazan crystals, especially in thick cultures at a wavelength of 450 nm, and measure the absorbance spectrophotometrically. The background absorbance of multiwell plates at 690 nm were subtracted from 450 nm data. A suitable plate reader can be used to read tests conducted on multiwell plates, or the contents of individual wells can be transferred to cuvettes of the right size for spectrophotometric analysis [10].

#### 3.2.4. In Silico Studies

##### Docking Studies

The structure of bacterial DNA gyrase (PDB code: 2XCT) was obtained from the Protein Data Bank. Molecular docking was conducted with MOE^®^ [46]. More potent compounds **6**, **7**, **15**, **16**, and the parent norfloxacin were docked into the active site of the gyrase enzyme (PDB: 2XCT) to propose a potential mechanism of action and ascertain various binding modes between the compounds and the enzyme’s active site. Docking studies were conducted with Molecular Operating Environment (MOE^®^) version 2014. The X-ray crystallographic structure of the ligand-enzyme complex was obtained from the Protein Data Bank (www.rcsb.org), specifically for topoisomerase II enzyme (PDB: 2XCT). The energy was minimized until achieving an RMSD (root mean square deviation) gradient of 0.01 kcal/mol and an RMS (root mean square) gap of 0.1 Å using the MMFF-94X (Merck Molecular Force Field 94x) force field, while the partial charges were automatically calculated *via* the builder interface of the MOE software, version 2014. The enzyme was prepared for docking investigations by eliminating the ligand, assessing atomic relationships, and automatically incorporating hydrogen atoms. The receptor’s potential was established, and the modeled chemicals were docked into the three-dimensional structure of the catalytic site of the acquired poses. The poses demonstrating the most efficacious ligand–enzyme interactions were selected and preserved for energy computations [22].

##### Physicochemical and Pharmacokinetic Prediction

The physicochemical parameters and pharmacokinetics of compounds **2**–**17** were predicted using Swiss ADME, a freely available service provided by the Swiss Institute of Bioinformatics [37]. The BOILED Egg graph depicts the relationship between TPSA and WLOGP, where the white area indicates the greatest likelihood of gastrointestinal absorption, while the yolk area reflects the highest possibility of BBB permeability. Lipophilicity is quantified using the consensus log Po/w, which is determined by SwissADME. This value is the average of five log P values predicted by various freely available models, namely XLOGP3, MLOGP, SILICOS-IT, iLOGP, and their own model WLOGP, which is also used in the BOILED Egg plot. The bioavailability radar encompasses six specific physicochemical properties: lipophilicity (with a range of −0.7 to +5.0 for XLOGP3), size (with a range of 150 g/mol to 500 g/mol for MV), polarity (with a range of 20 Å2 to 130 Å2 for TPSA), insolubility (with a range of 0 to 6 for Log S (ESOL)), unsaturation (with a range of 0.25 to 1.0 for Fraction Csp3), and flexibility (with a range of 0 to more than 9 for the number of rotatable bonds). The core pink hexagon symbolizes the ideal range for all six criteria. The Lipinski filter is employed to evaluate the drug-like properties of synthesized compounds. It considers the following criteria: molecular weight (MW) should be less than or equal to 500, the MLOGP value (a measure of lipophilicity) should be less than or equal to 4.15, the number of nitrogen or oxygen atoms should be less than or equal to 10, and the number of NH or OH groups should be less than or equal to 5.

## 4. Conclusions

Novel norfloxacin derivatives were synthesized as antibacterial agents. Results showed potent activities of compound **6–17** towards *S. aureus ATCC 6538,* especially compound **6,** the *N*-4-piperazinyl cyano derivative of norfloxacin, exhibited 37-fold more potency than norfloxacin. More importantly, compound **7,** the N-4-ethoxy carbonyl derivative, exhibited about double the potency of norfloxacin against *MRSA AUMC 261*. Meanwhile, carboxyl derivatives of norfloxacin **15** and **16** have potent activity towards the Gram-negative strains with MIC values of 0.20–0.79 µM comparable to norfloxacin with MIC of 0.24 µM. Importantly, the potent compounds showed higher activity towards topoisomerase II enzymes, especially against topoisomerase IV, which confirms the docking study with the *S. aureus* gyrase enzyme active binding site (PDB ID: 2XCT). Furthermore, the most potent compounds indicated minimal harmful effects when assessed against the normal cell line WI 38. Consequently, these compounds are regarded as promising antibacterial candidates, necessitating further modification. Moreover, one of the norfloxacin derivatives has twice the potency of norfloxacin against the resistant strain (MRSA AUMC 261), which is a crucial target in pharmaceutical research, and the study also provides newly synthesized derivatives with more potent activity against *S aureus ATCC 6538* compared with the parent norfloxacin. Hence, these compounds are considered promising antibacterial candidates that require further optimization.

## Figures and Tables

**Table 1:** Target compounds **2**–**17** and norfloxacin’s minimal inhibitory concentration (µM) towards *S. aureus ATCC 6538*, *E. coli ATCC 25922*, *K. pneumoniae ATCC 10031*, and *P. aeruginosa ATCC 27853*.

Compounds	Bacterial Strain			
	Gram +ve Strains	Gram −ve Strains		
	*S. aureus* *ATCC 6538*	*E. coli* *ATCC 25922*	*K. pneumoniae* *ATCC 10031*	*P. aeruginosa* *ATCC 27853*
**2**	>10	3.16 ± 0.10	1.58 ± 0.06	1.58 ± 0.05
**3**	>10	6.62 ± 0.22	3.31 ± 0.11	3.31 ± 0.12
**4**	>10	5.21 ± 0.19	1.30 ± 0.04	1.30 ± 0.06
**5**	>10	4.58 ± 0.17	>10	4.58 ± 0.21
**6**	**0.21** ± 0.01	1.69 ± 0.10	1.69 ± 0.08	0.85 ± 0.03
**7**	**1.60** ± 0.06	1.60 ± 0.04	1.60 ± 0.08	0.80 ± 0.02
**8**	**1.66** ± 0.07	3.31 ± 0.10	1.66 ± 0.07	0.83 ± 0.04
**9**	**3.61** ± 0.15	3.61 ± 0.13	0.90 ± 0.03	0.90 ± 0.04
**10**	**3.46** ± 0.11	3.46 ± 0.09	0.86 ± 0.04	0.86 ± 0.02
**11**	**2.86** ± 0.09	2.86 ± 0.10	1.43 ± 0.06	2.86 ± 0.11
**12**	**1.58** ± 0.05	3.16 ± 0.12	3.16 ± 0.10	1.58 ± 0.06
**13**	**1.47** ± 0.05	5.88 ± 0.21	2.94 ± 0.09	1.47 ± 0.06
**14**	**1.49** ± 0.07	2.98 ± 0.13	2.98 ± 0.11	0.75 ± 0.02
**15**	**1.58** ± 0.06	0.40 ± 0.19	0.79 ± 0.02	0.20 ± 0.01
**16**	**1.28** ± 0.04	0.32 ± 0.14	0.64 ± 0.02	0.32 ± 0.01
**17**	**1.54** ± 0.04	>10	0.77 ± 0.02	1.54 ± 0.06
**norfloxacin 1**	7.83 ± 0.30	0.24 ± 0.01	0.24 ± 0.01	0.24 ± 0.01

Bold results mean more potent than the parent compound norfloxacin.

**Table 2:** MICs of compounds **6**, **7,** and **norfloxacin** as parent compounds against *MRSA AUMC 261* in (µM).

Compound	MIC Against *Staphylococcus MRSA AUMC 261* (µM)
**6**	>20
**7**	0.80 ± 0.04
**15**	6.33 ± 0.22
**16**	>20
**Norfloxacin**	1.60 ± 0.08

Bold results mean more potent than the parent compound norfloxacin.

**Table 3:** Gyrase and topoisomerase IV enzyme inhibition of compounds **6**, **7**, **15**, **16**, and norfloxacin (IC_50_ in µM ± SD).

Compound	Topoisomerase IV IC_50_ (µM ± SD)	DNA Gyrase IC_50_ (µM) ±SD
**6**	3.00 ± 0.12	7.10 ± 0.29
**7**	2.40 ± 0.07	4.07 ± 0.16
**15**	9.99 ± 0.19	17.17 ± 0.69
**16**	1.91 ± 0.04	3.57 ± 0.14
**Norfloxacin**	11.92 ± 0.42	2.28 ± 0.09

Bold results refer to a higher potency than the parent norfloxacin.

**Table 4:** The cytotoxicity of the newly synthesized compounds **6**, **7**, **15**, **16**, **norfloxacin**, and **doxorubicin** against the normal cell line W1 38.

Compound	Cytotoxicity Against Normal Cell Line WI 38 IC_50_ uM	SD ±
**Norfloxacin**	54.57	1.94
**6**	34.06	1.21
**7**	47.92	1.71
**15**	39.16	1.39
**16**	51.24	1.82
**Doxorubicin**	14.11	0.50

**Table 5:** Docking of compounds **6**, **7**, **15**, **16**, and **norfloxacin** into the active site of *S. aureus* DNA gyrase (PDB: 2XCT), their energy scores (kcal/mol), and binding interactions amongst the compounds.

Compound	Energy Score (kcal/mol)	Amino Acid Residue	Interaction Type	Length
**norfloxacin**	−8.33	DG 59	Pi-Pi	3.92
		MN 2000	Metal interaction	1.69
		MN 2000	Metal interaction	2.31
**6**	−10.32	MN 2000	Metal interaction	1.63
		DG 9	Pi-Pi	3.68
		MN 2000	Metal interaction	2.01
**7**	−9.80	DG 9	Pi-Pi	3.84
		SER 1048	Hydrogen bond	3.13
		SER 1048	Hydrogen bond	2.79
		MN 2000	Metal interaction	2.52
		MN 2000	Metal interaction	1.75
		DG 9	Pi-Pi	3.97
		DA 13	Pi-Pi	3.85
**15**	−7.84	MN 2000	Metal interaction	1.61
**16**	−10.94	ARG 458	Hydrogen bond	2.85
		DG 9	Pi-Pi	3.88
		MN 2000	Metal interaction	2.11
		MN 2000	Metal interaction	1.72

## Data Availability

The original contributions presented in this study are included in the article; further inquiries can be directed to the corresponding author.

## Supplementary Materials

The following supporting information can be downloaded at: https://www.mdpi.com/article/10.3390/ph18040545/s1, Figures S1–S46: NMR and Mass data, Figures S47–S49: Screening results of the target compounds and references against topo IV, Gyrase, and normal cell line WI 38, respectively; Tables S1–S4: Antibacterial screening of the target compounds in comparison to the reference norfloxacin and mechanistic study of the potent compounds, Table S5: Cytotoxicity of the potent antibacterial compounds against normal cell line WI 38, Table S6–S10: Physiochemical parameters of the target compounds in comparison to the reference norfloxacin.
