# Supplementary material for: Design, Synthesis, and In Silico Studies of New Norfloxacin Analogues with Broad Spectrum Antibacterial Activity via Topoisomerase II Inhibition
Source: Pharmaceuticals (Basel). 2025 Apr 8;18(4):545. doi: 10.3390/ph18040545 (PMC12030355; doi:10.3390/ph18040545)
Supplement: Supplementary file 1 [file pharmaceuticals-18-00545-s001.zip › pharmaceuticals-3543440-supplementary.pdf]

Supporting information

Design, Synthesis, and *In Silico* Studies of New Norfloxacin Analogues with Broad Spectrum Antibacterial Activity *via* Topoisomerase II Inhibition

Ahmed M. El-Saghier [1,*], Laila Abosella [1,2], Abdelfattah Hassan [3,4,] Esmail O. Elakesh [5], Stefan Bräse [6,*], Gamal El-Din A. Abuo-Rahma [7,8] and Hossameldin A. Aziz [9]

1 Chemistry Department, Faculty of Science, Sohag University, Sohag 82524, Egypt; lailaabosella@gmail.com
2 Medicinal Chemistry Department, Faculty of Pharmacy—Al-Jmail, Sabratha University, Sabratha 250, Libya
3 Medicinal Chemistry Department, Faculty of Pharmacy, South Valley University, Qena 83523, Egypt; abdelfattah_hassan@svu.edu.eg. https://orcid.org/0000-0001-8539-662X
4 Medicinal Chemistry Department, Faculty of Pharmacy, National South Valley University, Qena 83523, Egypt; abdelfattah_hassan@svu.edu.eg.
5 Chemistry Department, Faculty of Science, Al Zawiya University, Al Zawiya 16418, Libya; esmail_elakesh@zu.edu.ly
6 Institute for Biological and Chemical System, Karlsruhe Institute of Technology, 76131 Karlsruhe, Germany
7 Medicinal Chemistry Department, Faculty of Pharmacy, Minia University, Minia 61519, Egypt; gamal.aborahma@mu.edu.eg
8 Pharmaceutical Chemistry Department, Faculty of Pharmacy, Deraya University, New Minia-61768, Minia, Egypt
9 Pharmaceutical Chemistry Department, Faculty of Pharmacy, New Vallely University, New Valley 72511, Egypt; hossamaziz85@pha.nvu.edu.eg. https://orcid.org/0009-0005-3165-3025
* Correspondence : el.saghier@science.sohag.edu.eg (A.M.E.-S.); stefan.braese@kit.edu (S.B.)

1-Chemistry

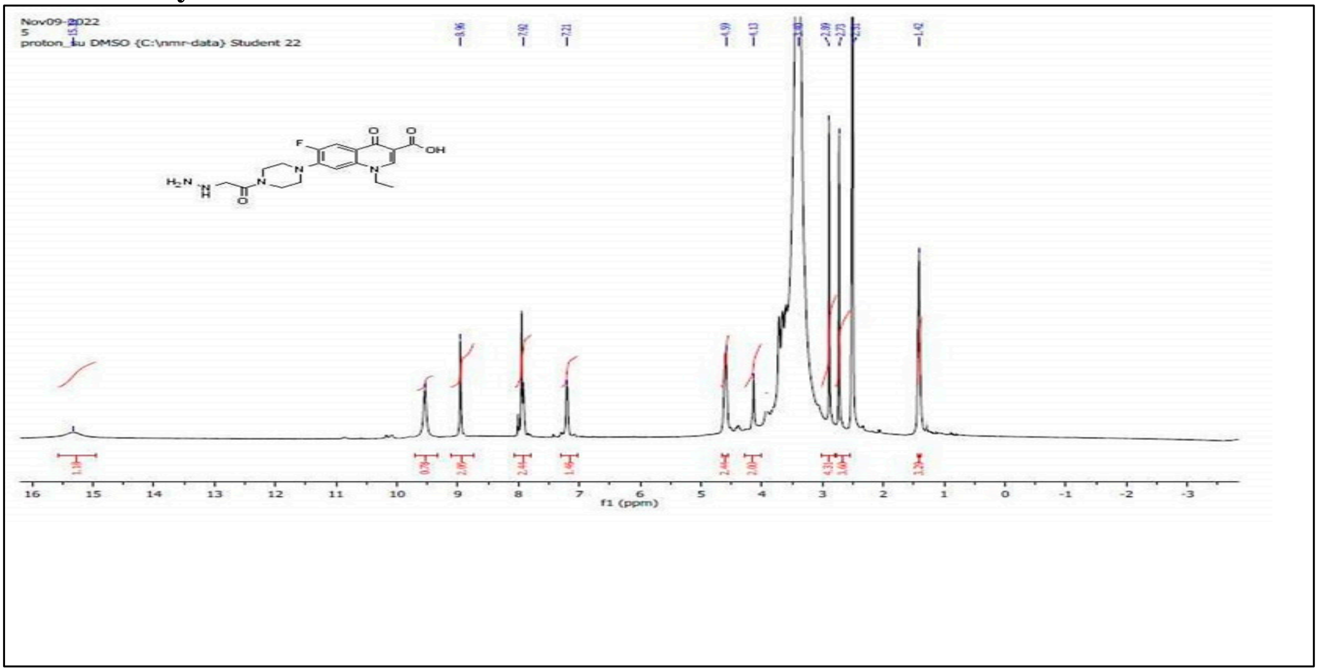

Figure S1. ^{1}HNMR spectrum of compound **3** (400 MHz, DMSO-d_6)

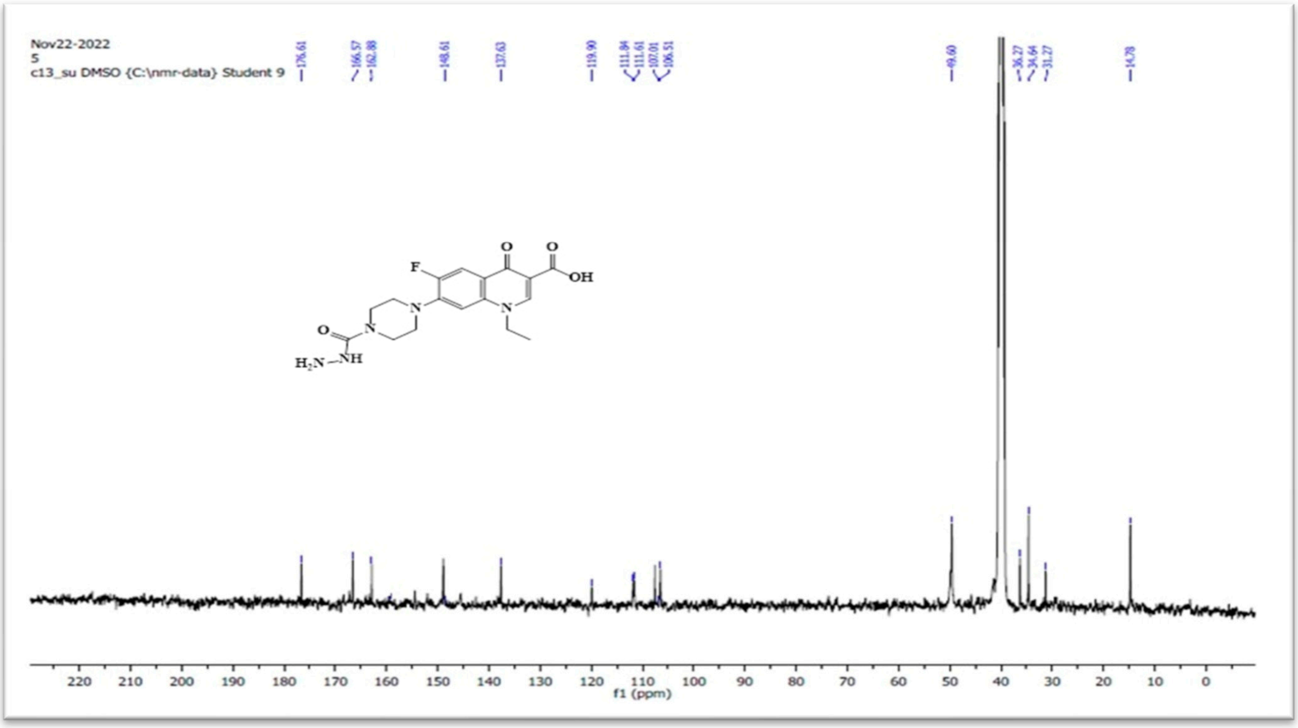

Figure S2. ^{13}CNMR spectrum of compound **3** (100 MHz, DMSO-d_6)

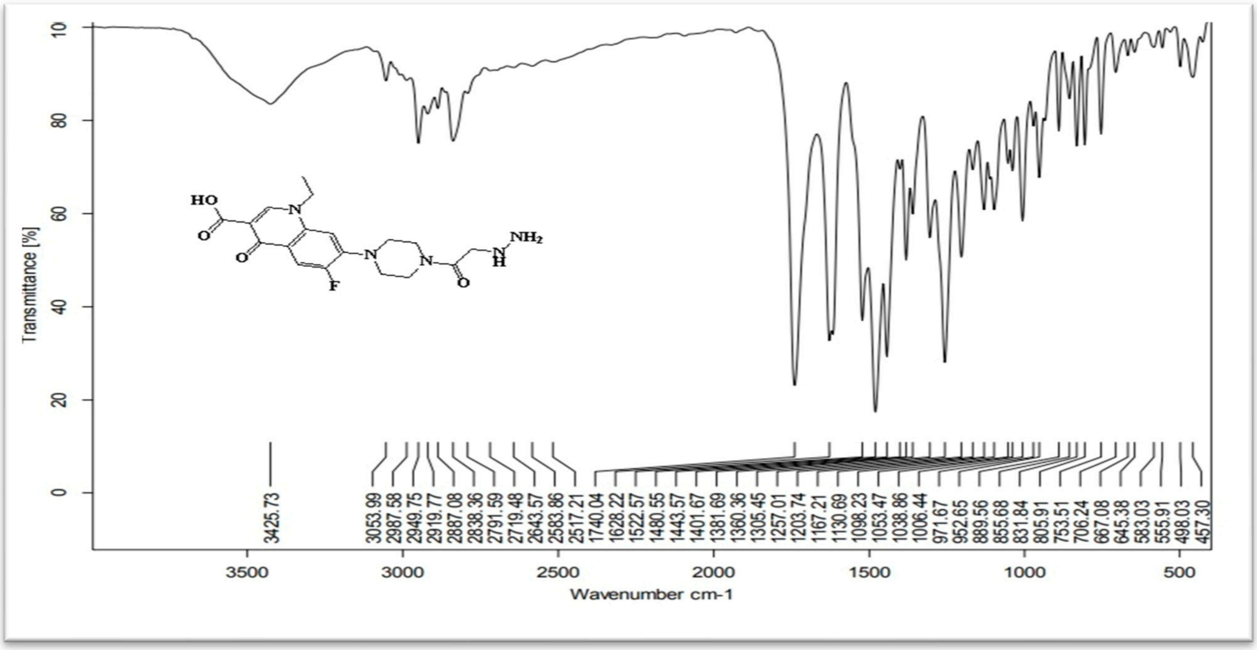

Figure S3. IR spectrum of compound **3**

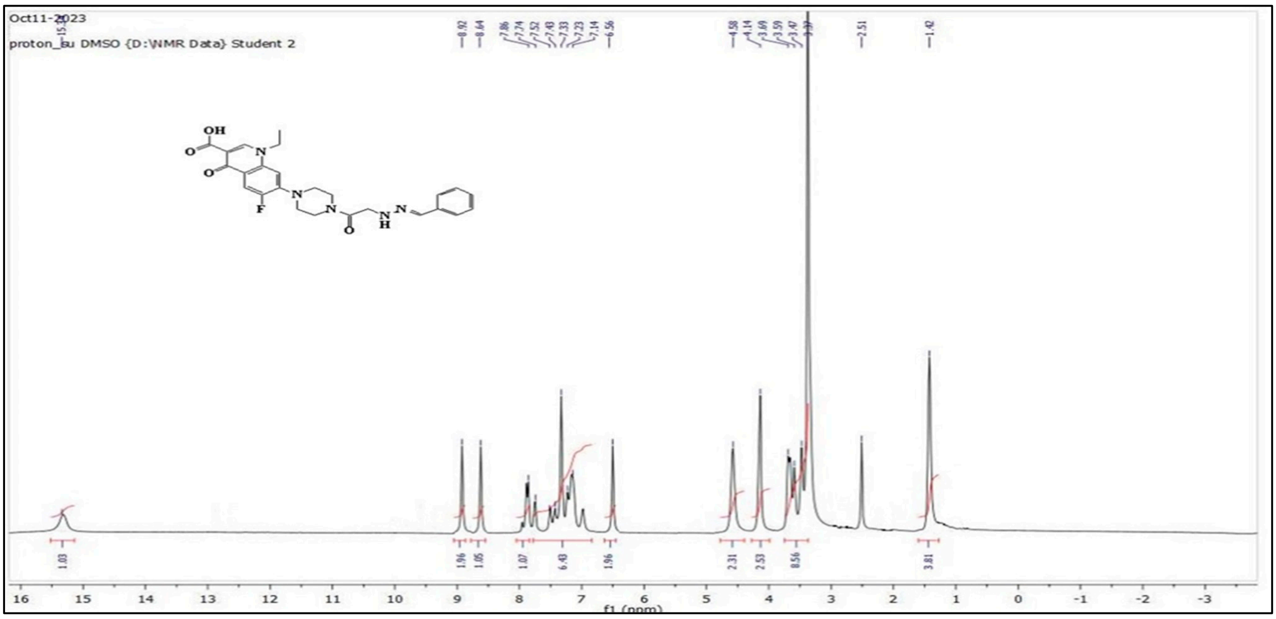

Figure S4. ^{1}HNMR spectrum of compound **4** (400 MHz, DMSO-d_6)

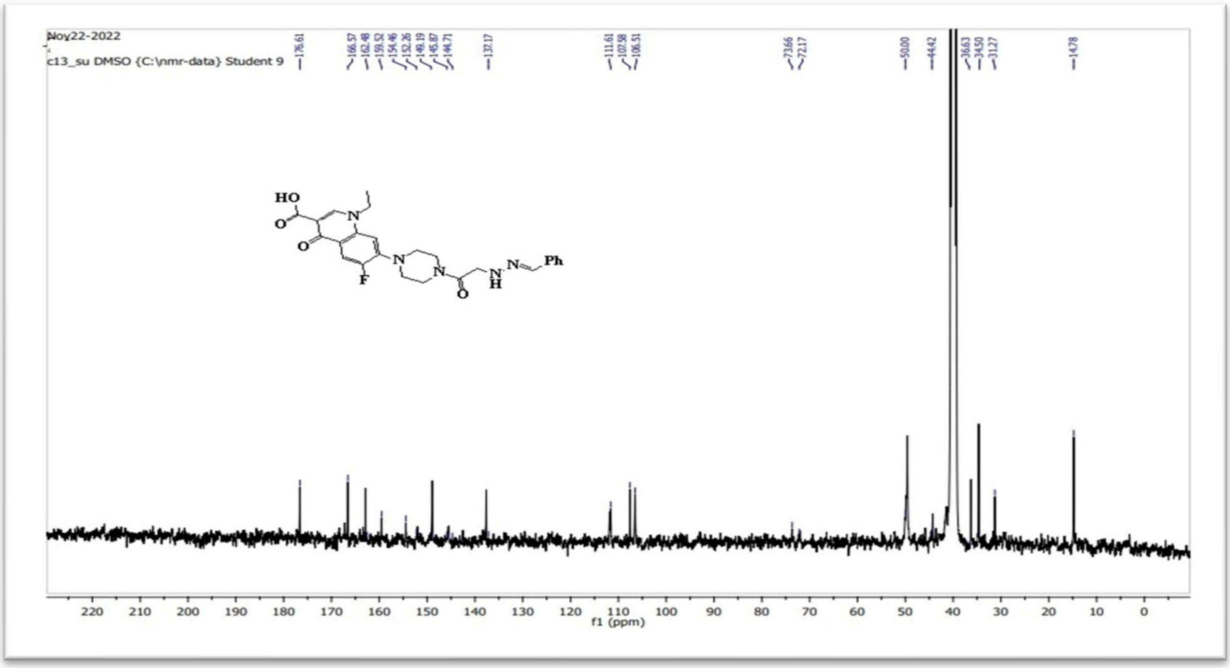

Figure S5. ^{13}CNMR spectrum of compound **4** (100 MHz, DMSO-d_6)

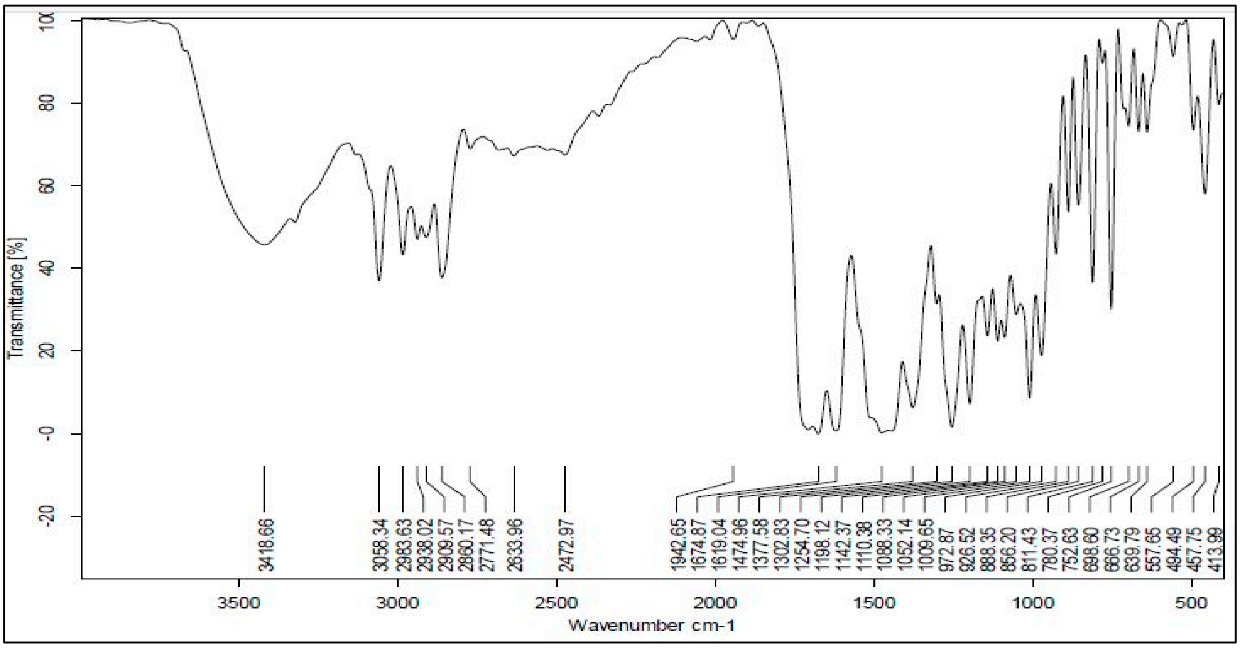

Figure S6. IR spectrum of compound **4**

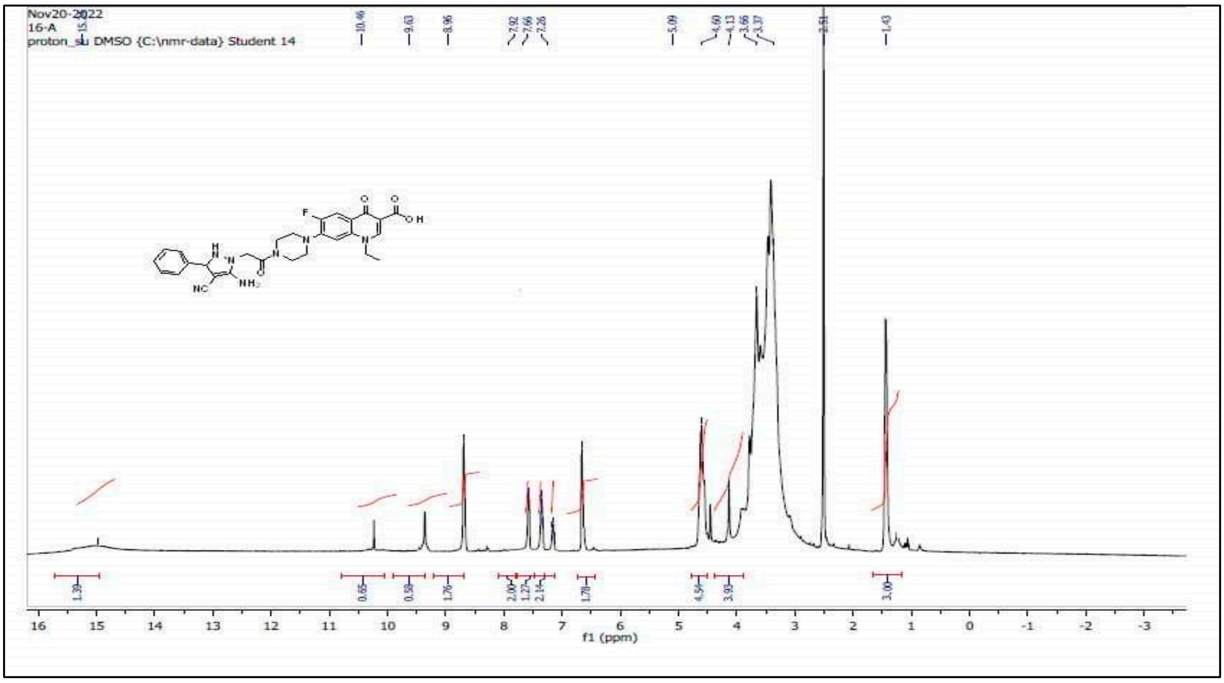

Figure S7. ^{1}HNMR spectrum of compound **5** (400 MHz, DMSO-d_6)

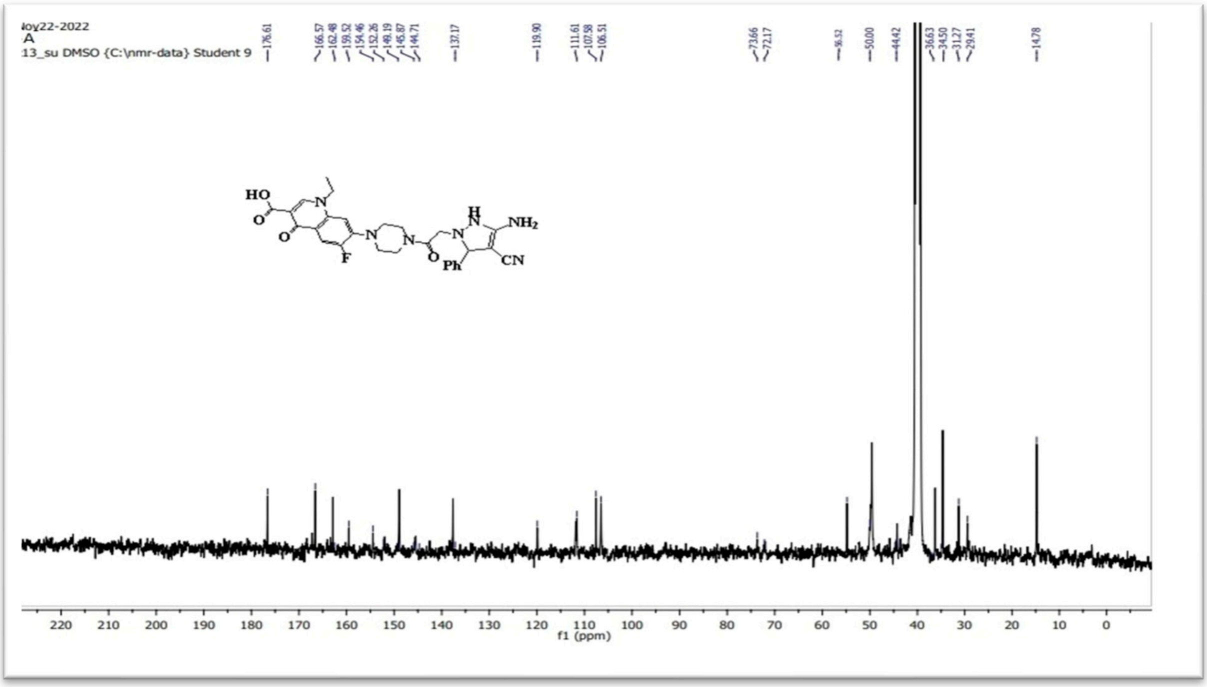

Figure S8. ^{13}CNMR spectrum of compound **5** (100 MHz, DMSO-d_6)

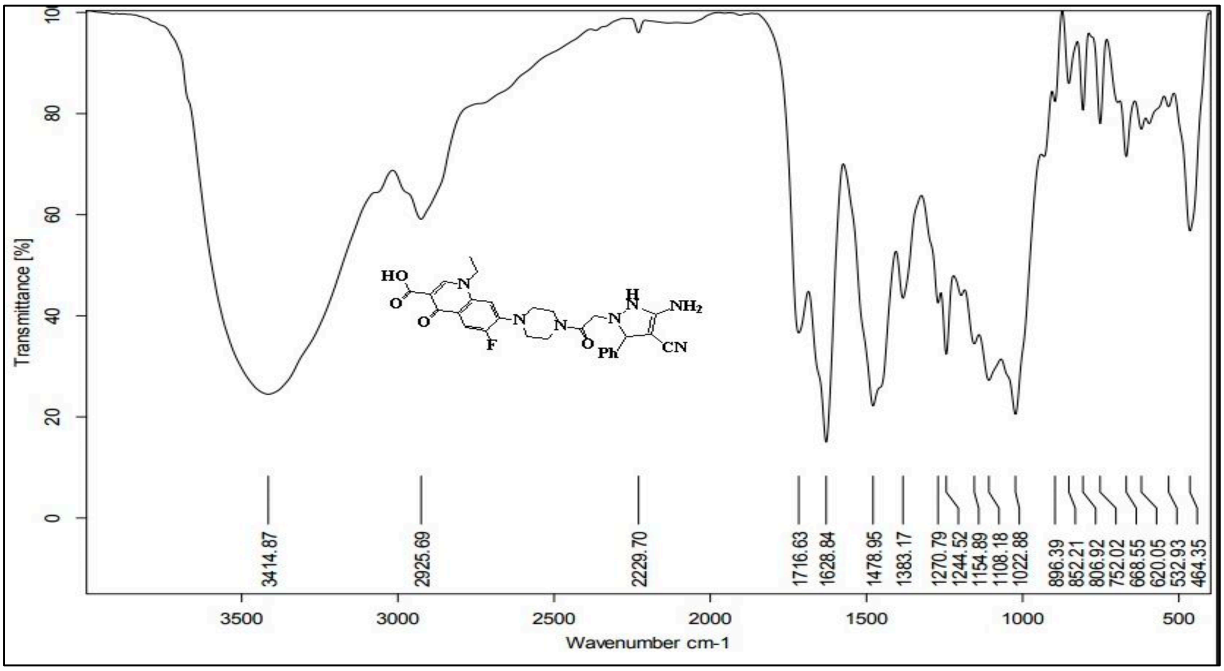

Figure S9. IR spectrum of compound **5**

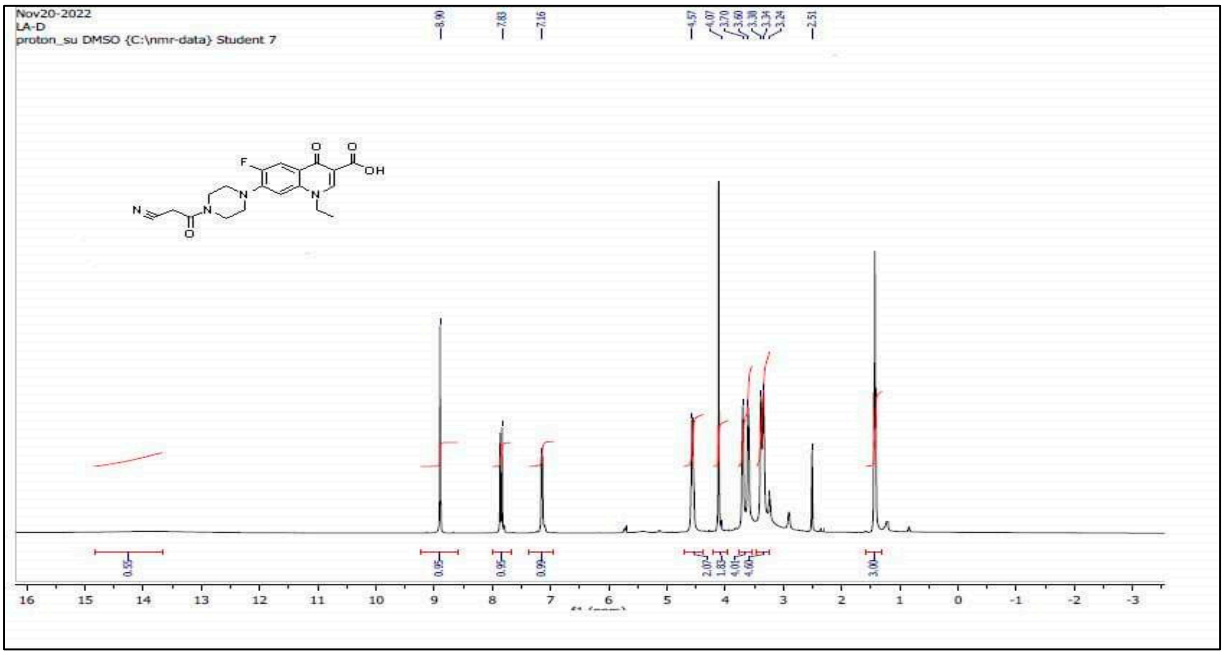

Figure S**10**. ^{1}HNMR spectrum of compound **6** (400 MHz, DMSO-d_6)

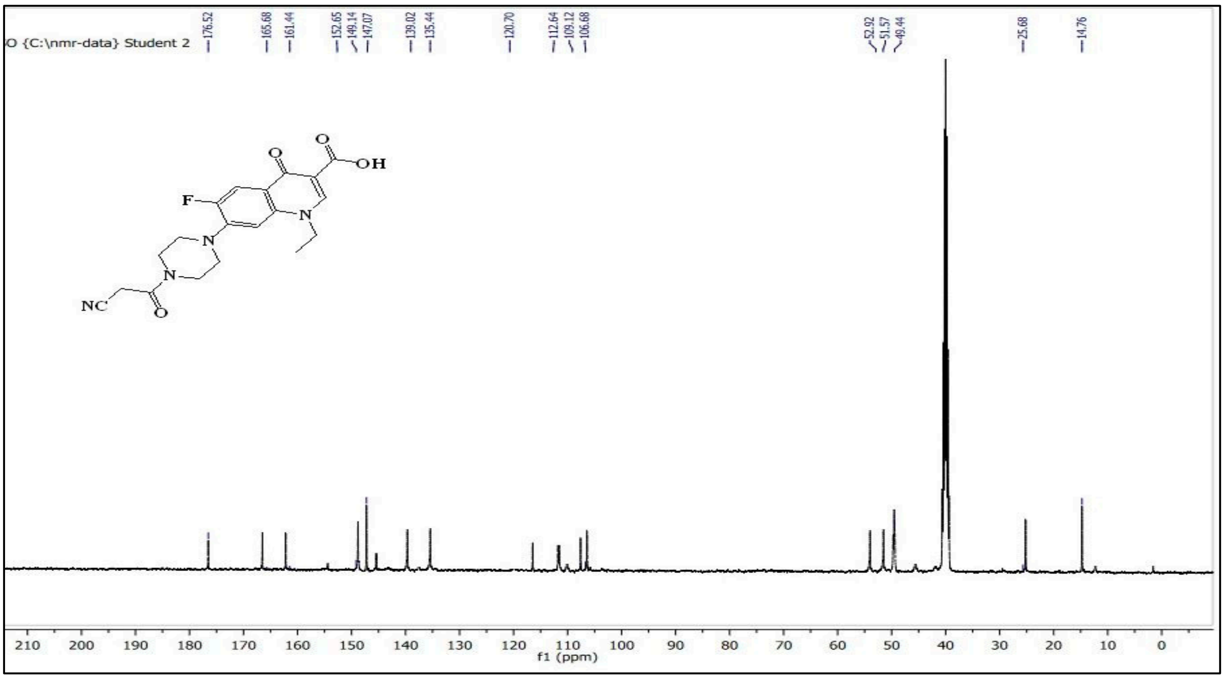

Figure S11. ^{13}CNMR spectrum of compound **6** (100 MHz, DMSO-*d*$_6$)

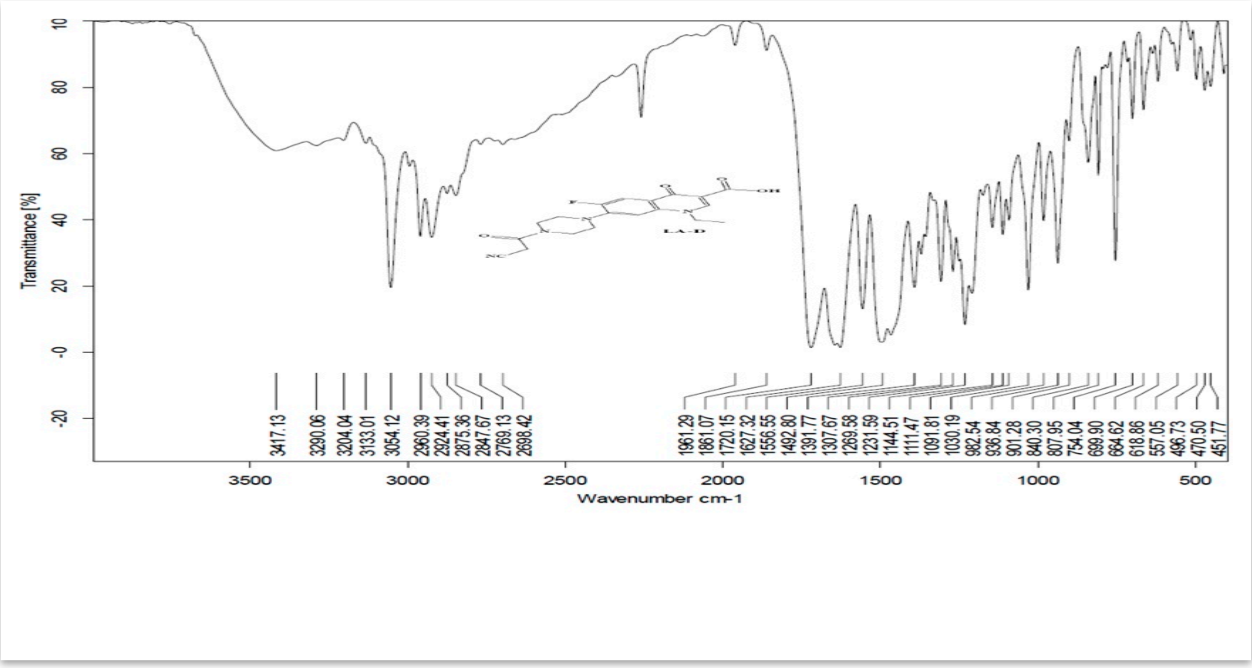

Figure S12. IR of compound **6**

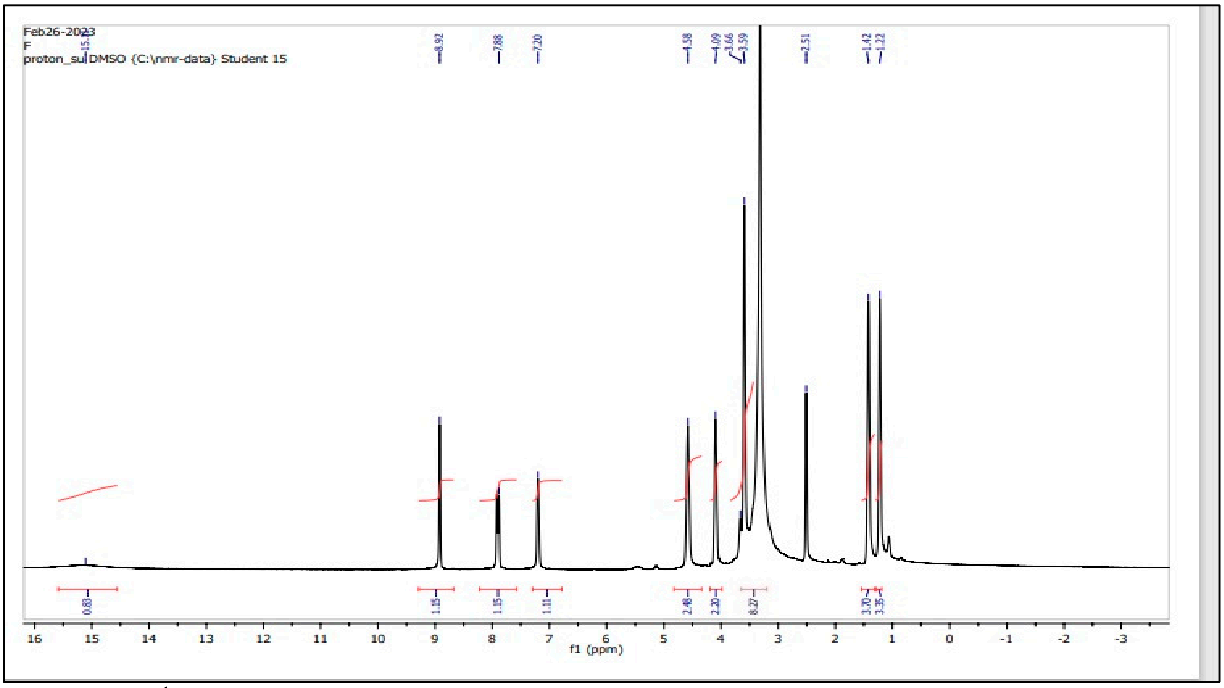

Figure S13. ^{1}HNMR spectrum of compound **7** (400 MHz, DMSO-*d6*)

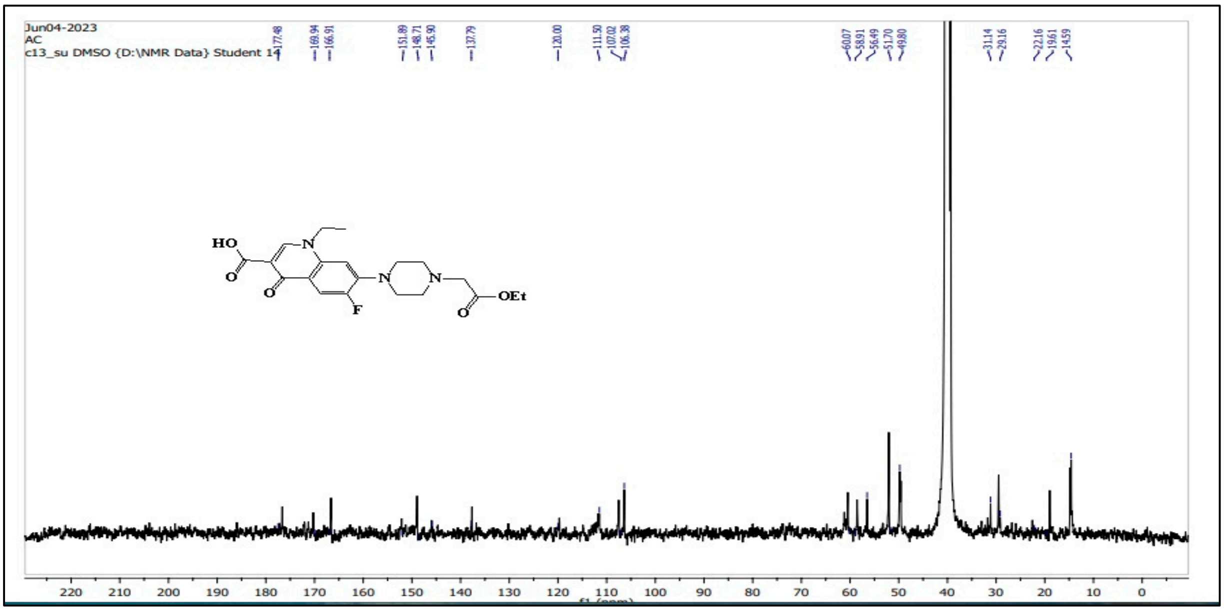

Figure S14. ^{13}CNMR spectrum of compound **7** (100 MHz, DMSO-*d6*)

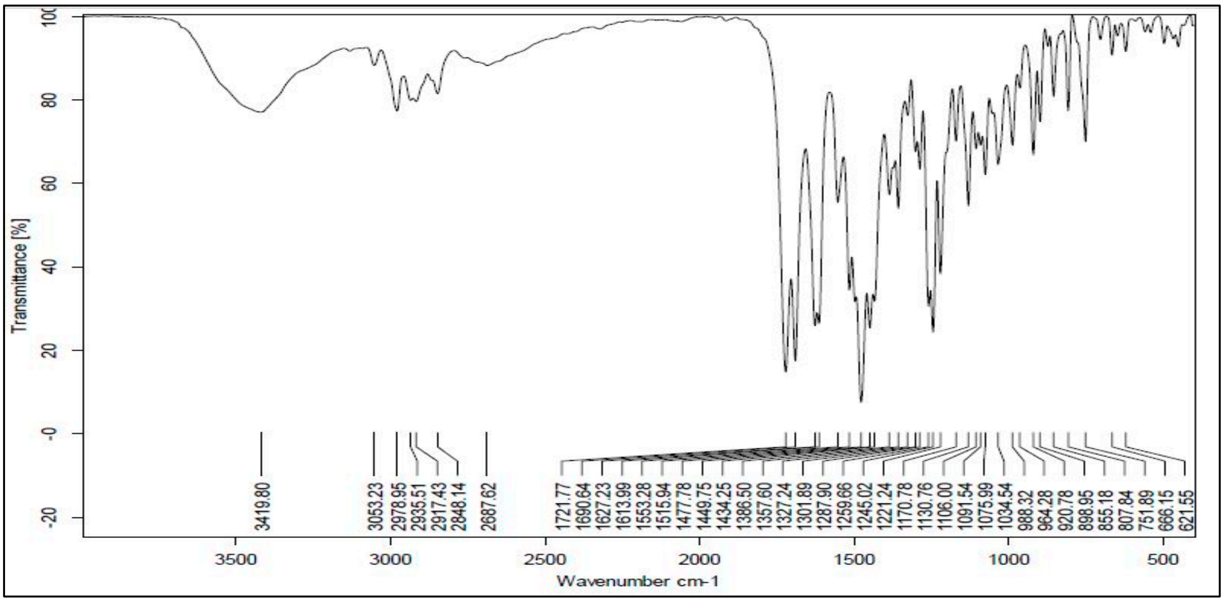

Figure S15. IR of compound **7**

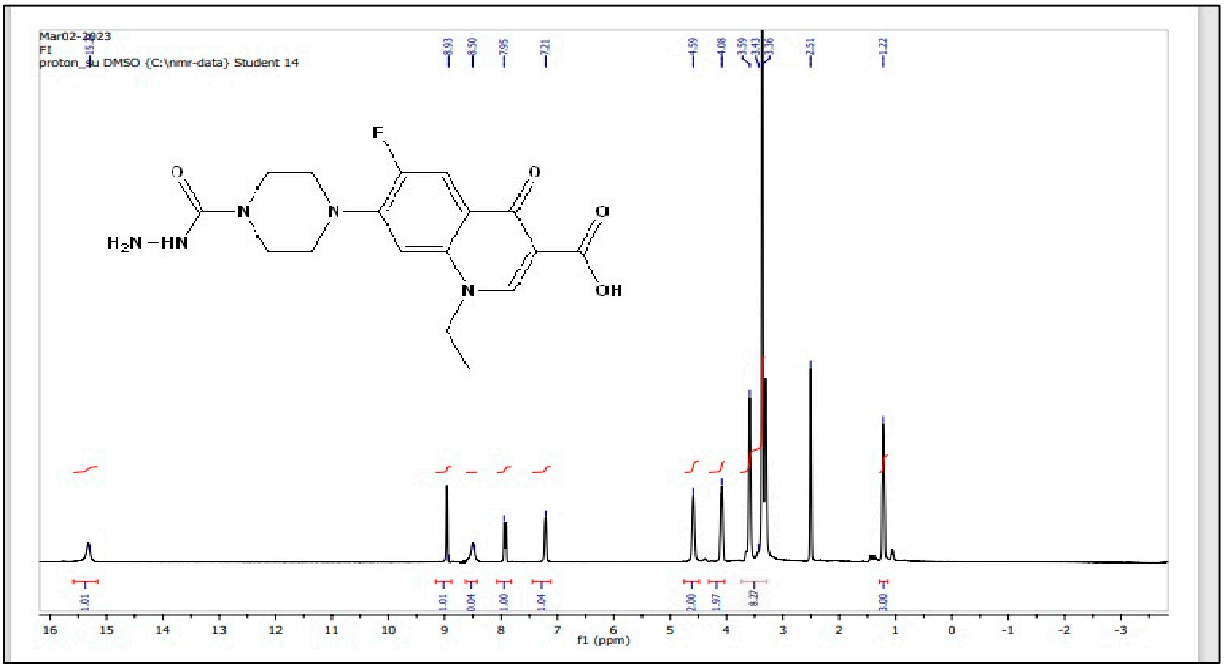

Figure S16. ^{1}HNMR spectrum of compound **8** (400 MHz, DMSO-*d6*)

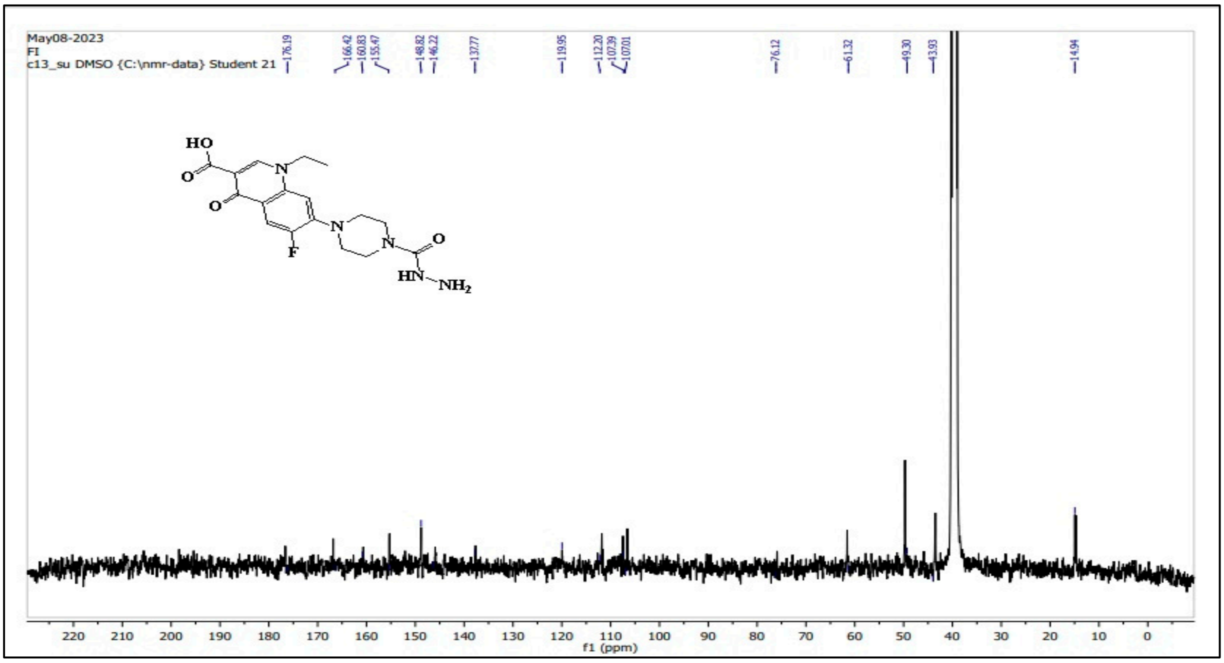

Figure S17. ^{13}CNMR spectrum of compound **8** (100 MHz, DMSO-d_6)

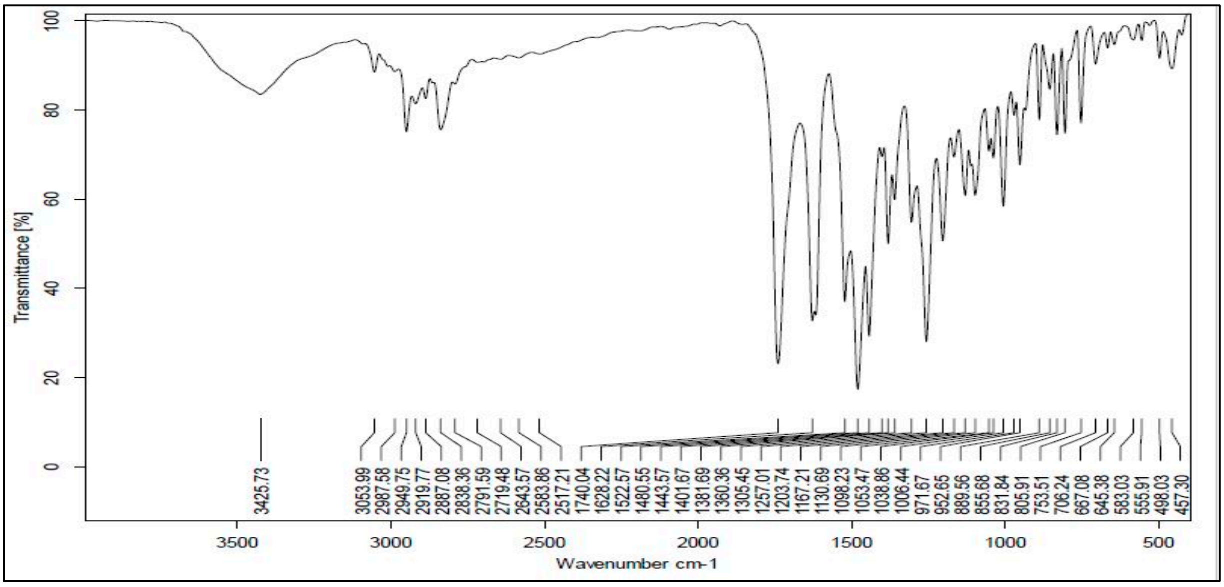

Figure S18. IR of compound **8**

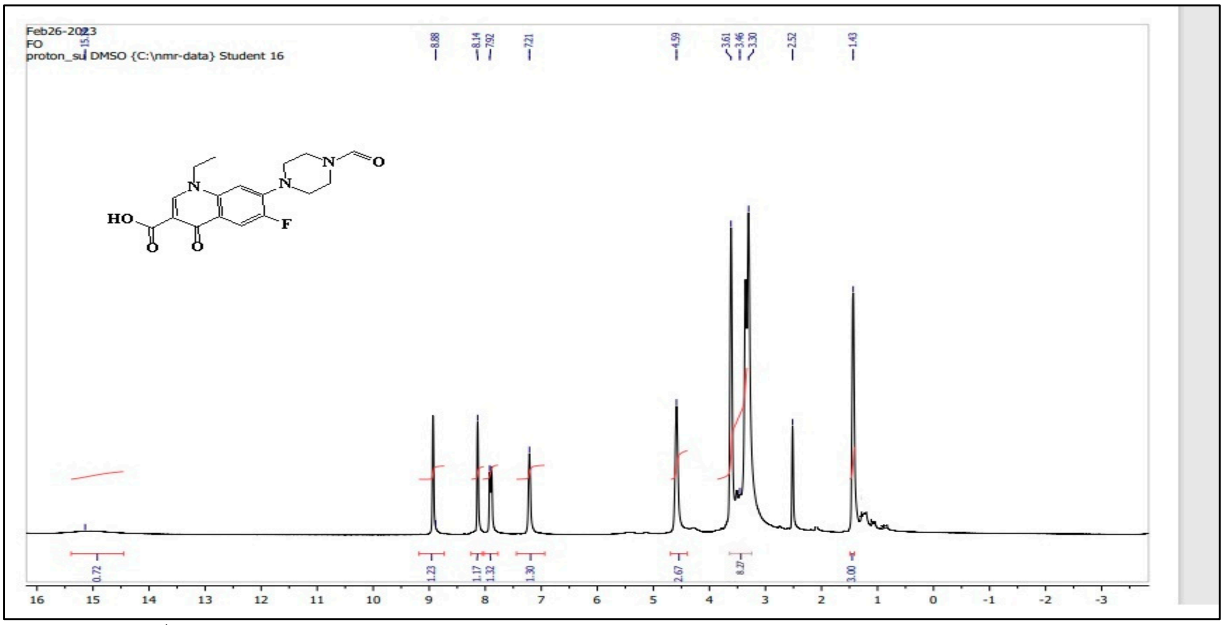

Figure S19. ^{1}HNMR spectrum of compound **9** (400 MHz, DMSO-*d6*)

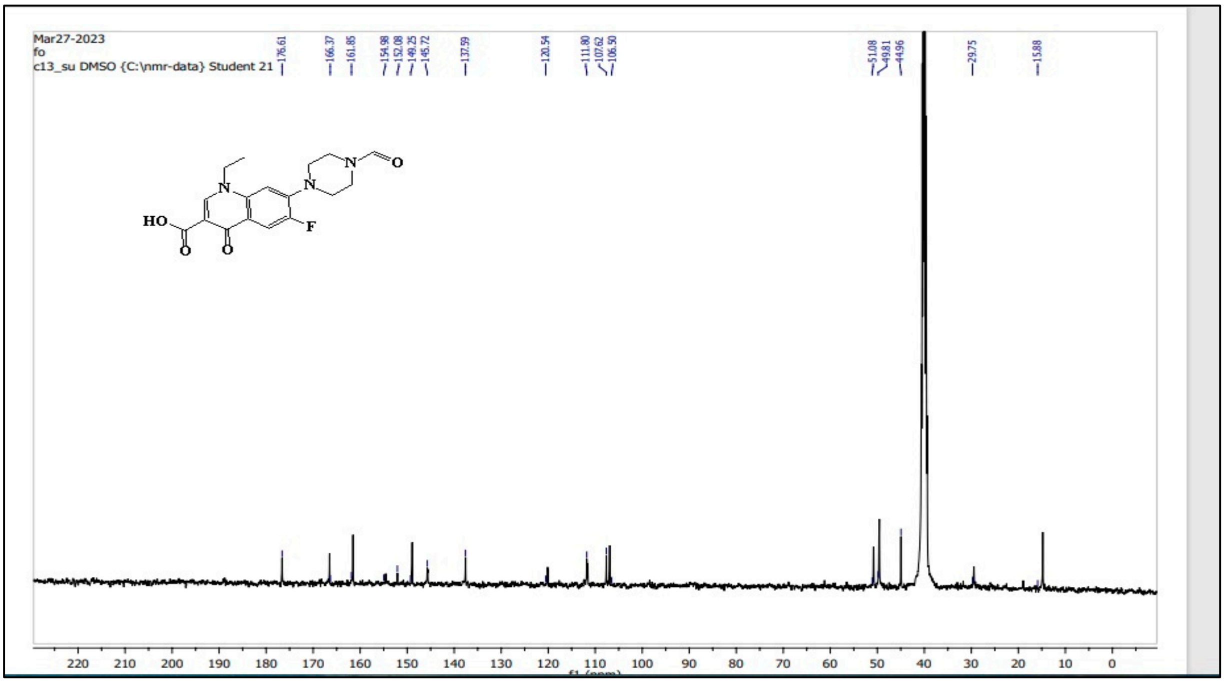

Figure S20. ^{13}CNMR spectrum of compound **9** (100 MHz, DMSO-d_6)

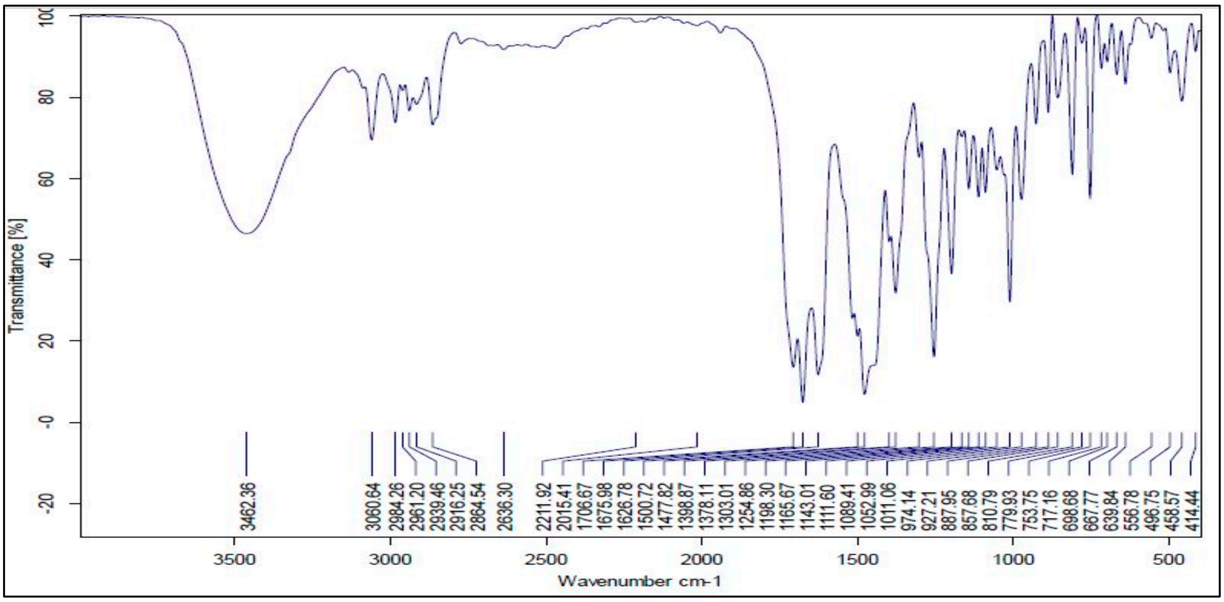

Figure S21. IR of compound **9**

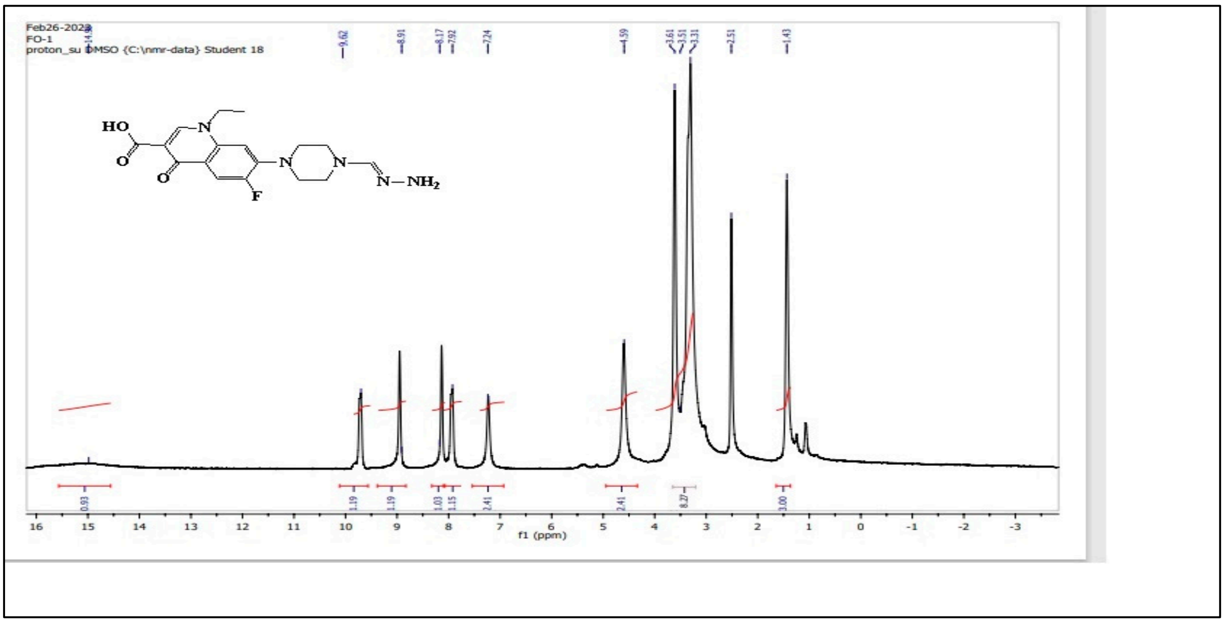

Figure S22. ^{1}HNMR spectrum of compound **10** (400 MHz, DMSO-*d6*)

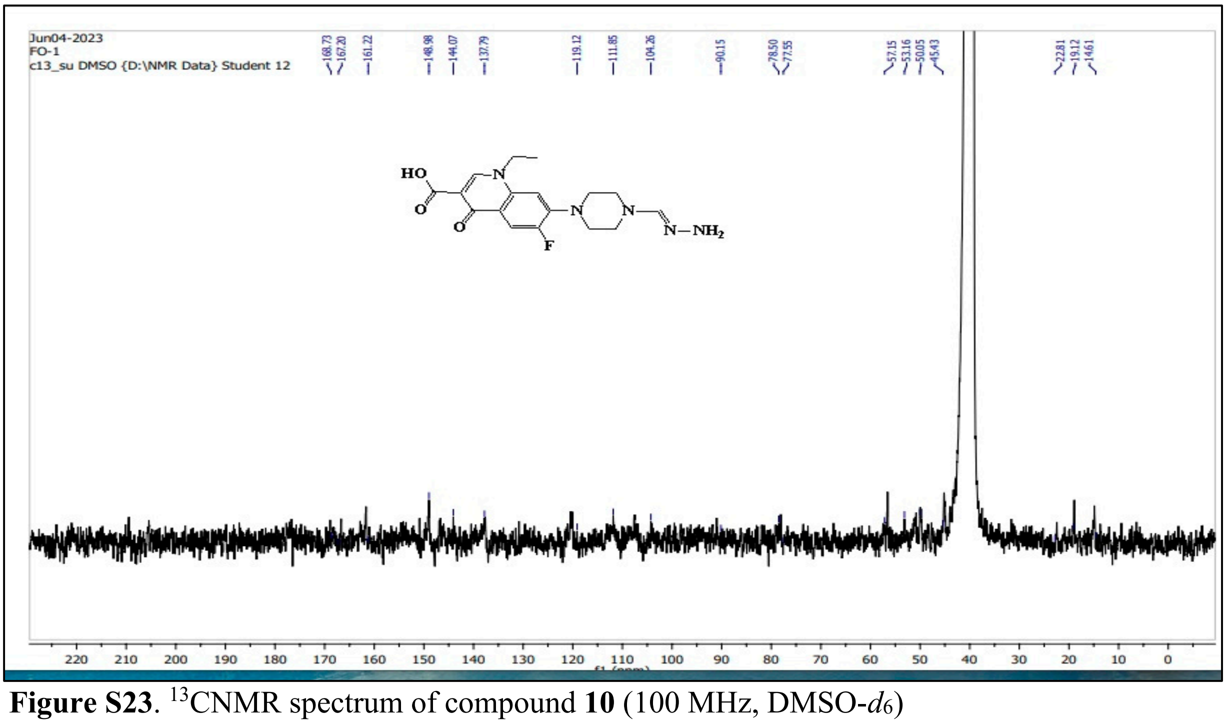

Figure S23. ^{13}CNMR spectrum of compound **10** (100 MHz, DMSO-d_6)

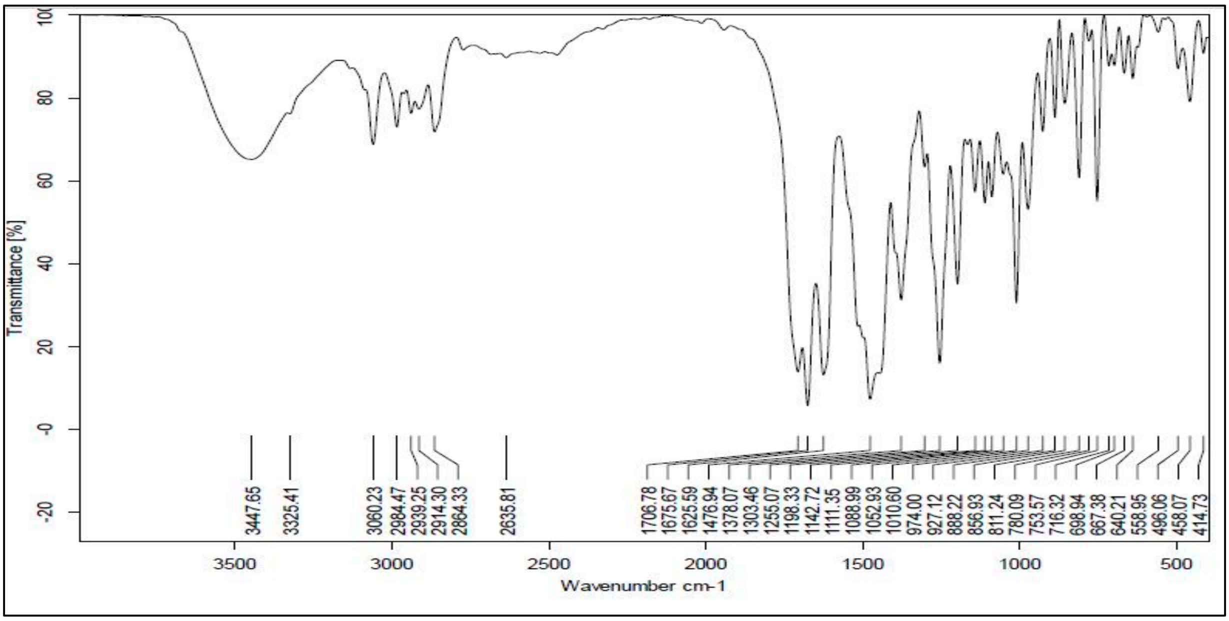

Figure S24. IR of compound **10**

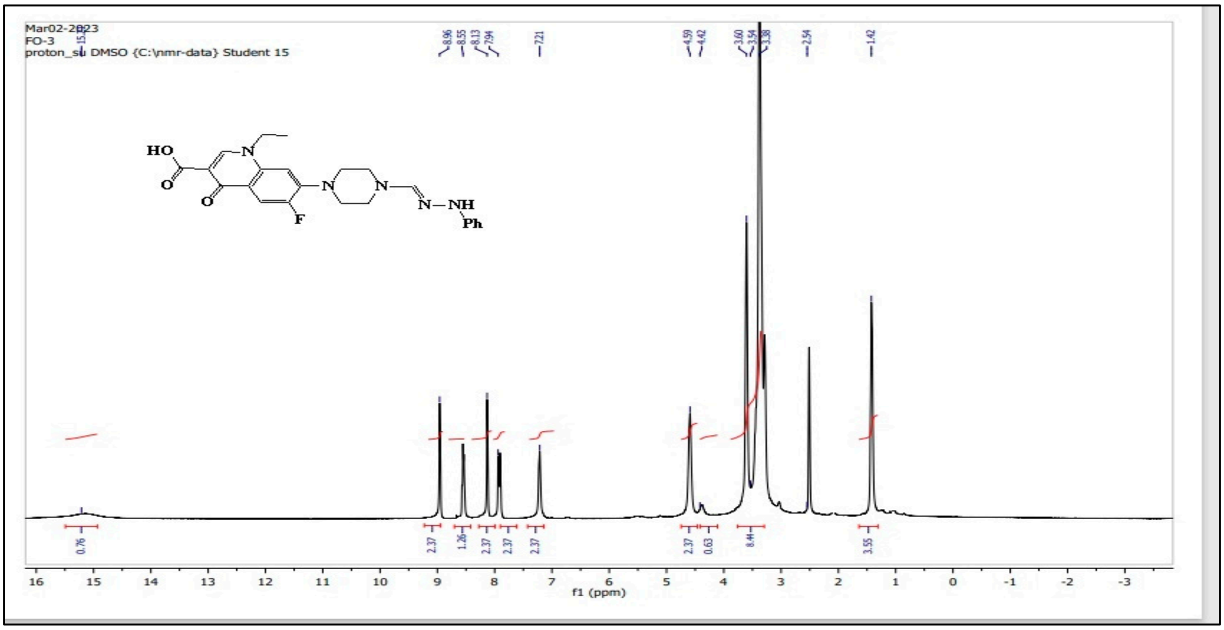

Figure S25. ^{1}HNMR spectrum of compound **11** (400 MHz, DMSO-d6)

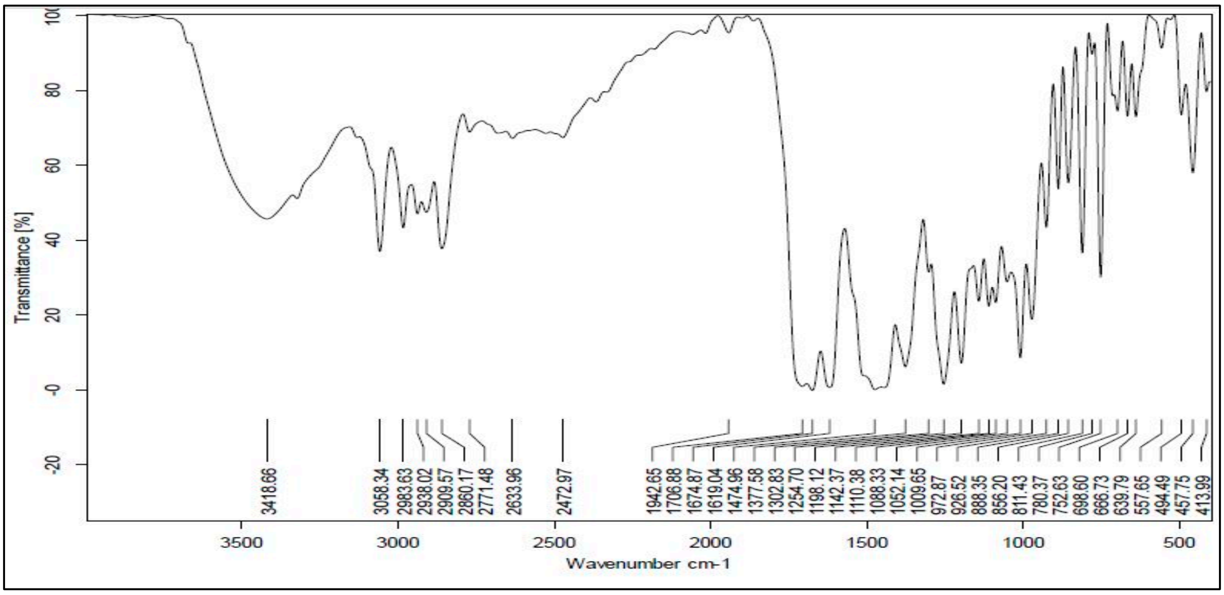

Figure S26. IR of compound **11**

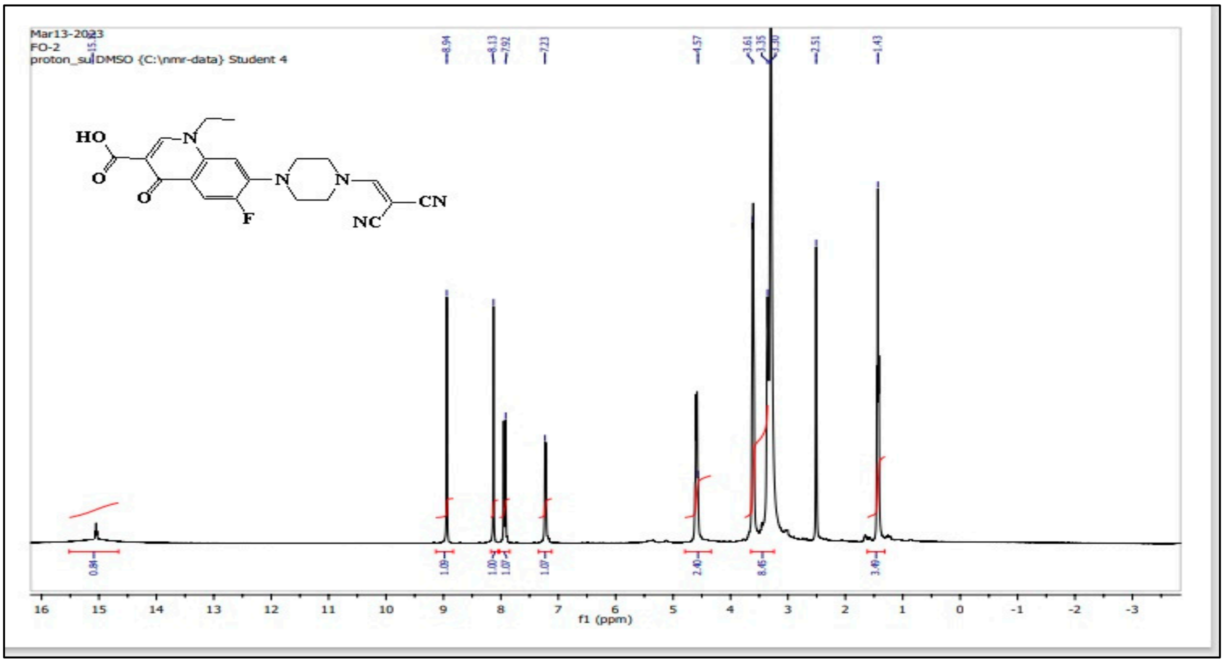

Figure S27. ^{1}HNMR spectrum of compound **12** (400 MHz, DMSO-*d6*)

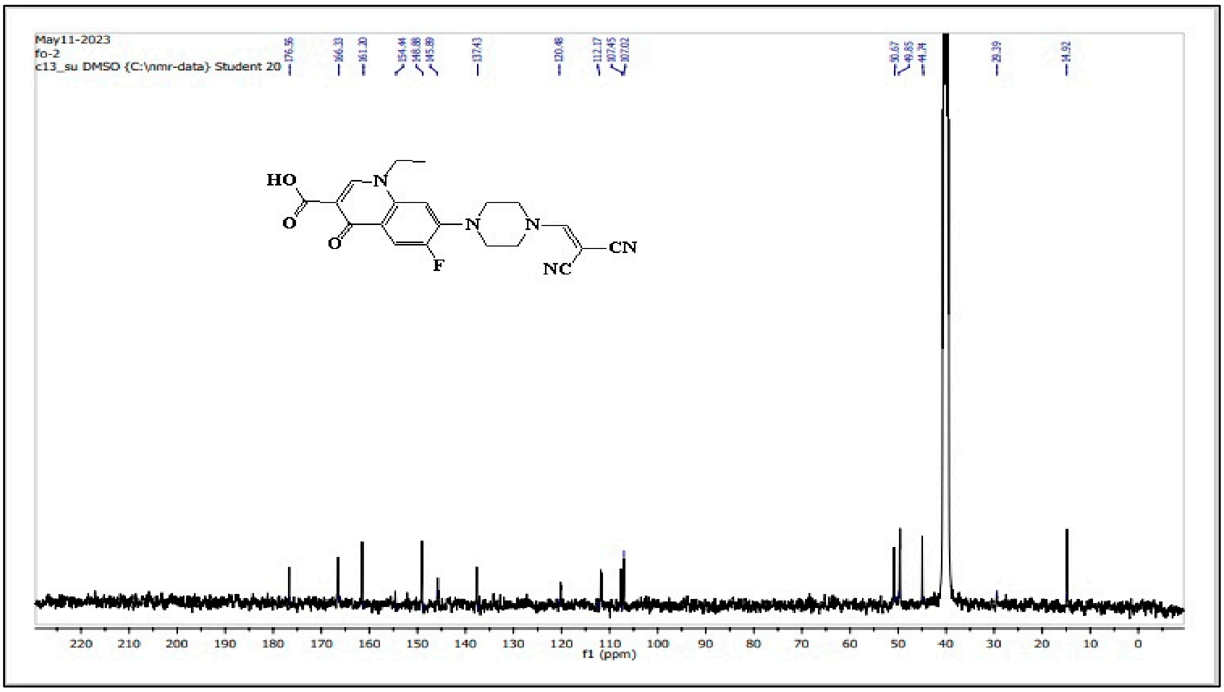

Figure S28. ^{13}CNMR spectrum of compound **12** (100 MHz, DMSO-d_6)

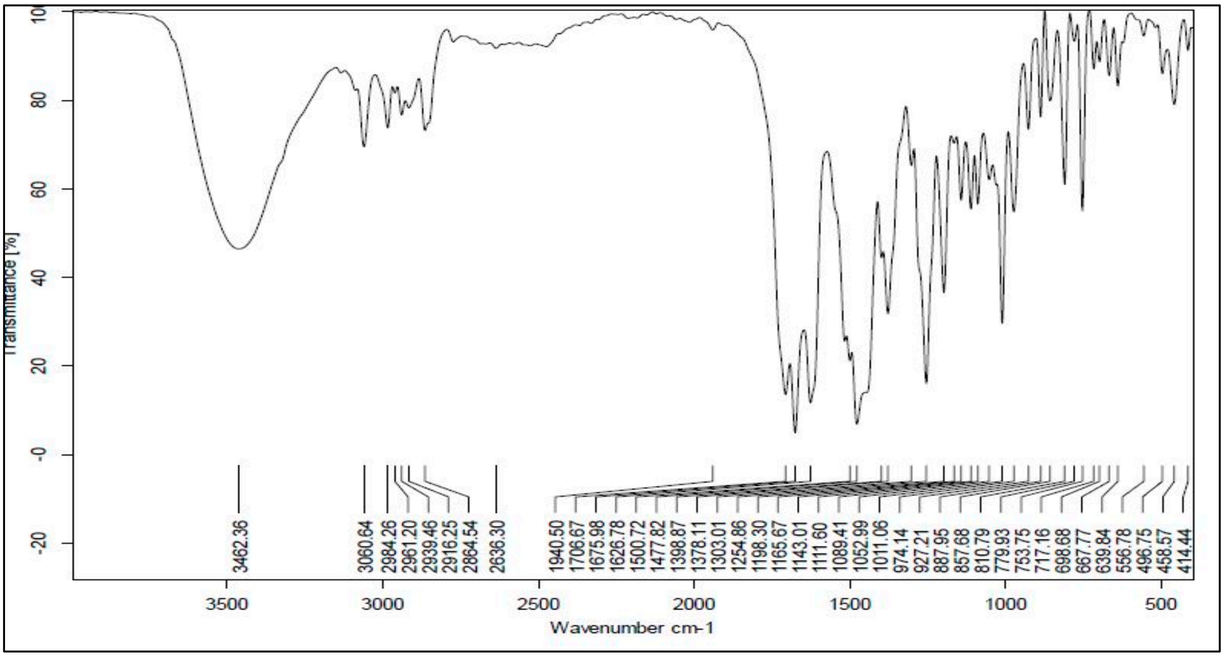

Figure S29: IR of compound **12**

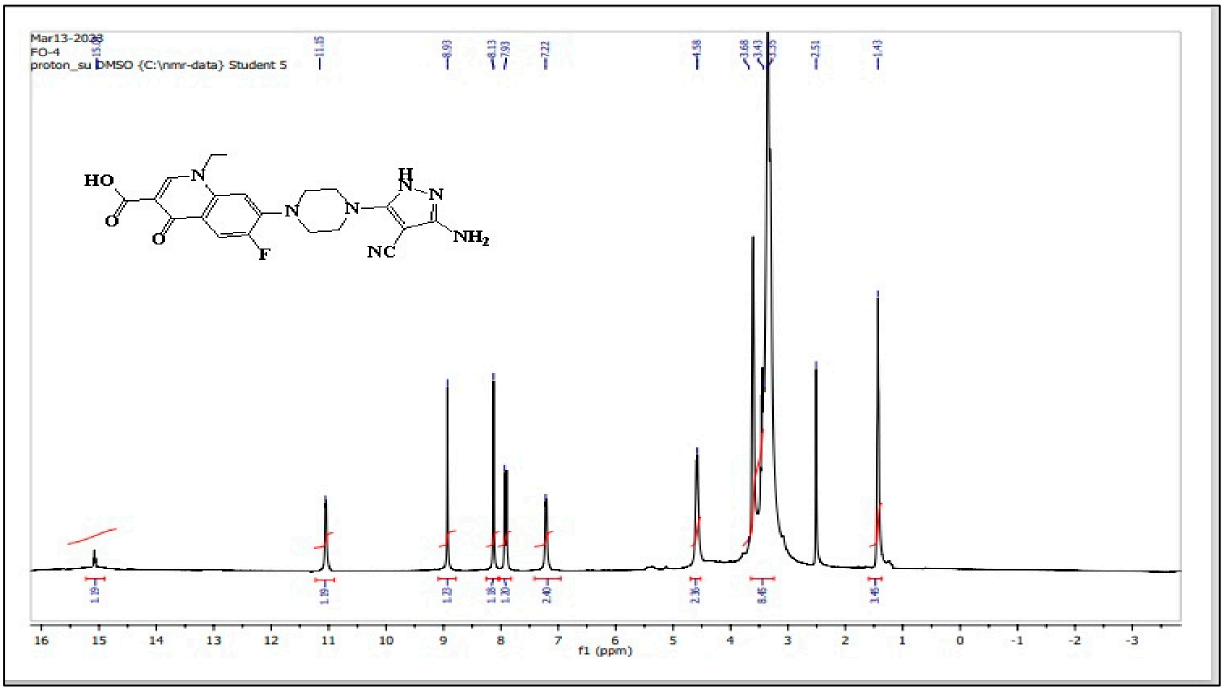

Figure S30. ^{1}HNMR spectrum of compound **13** (400 MHz, DMSO-*d6*)

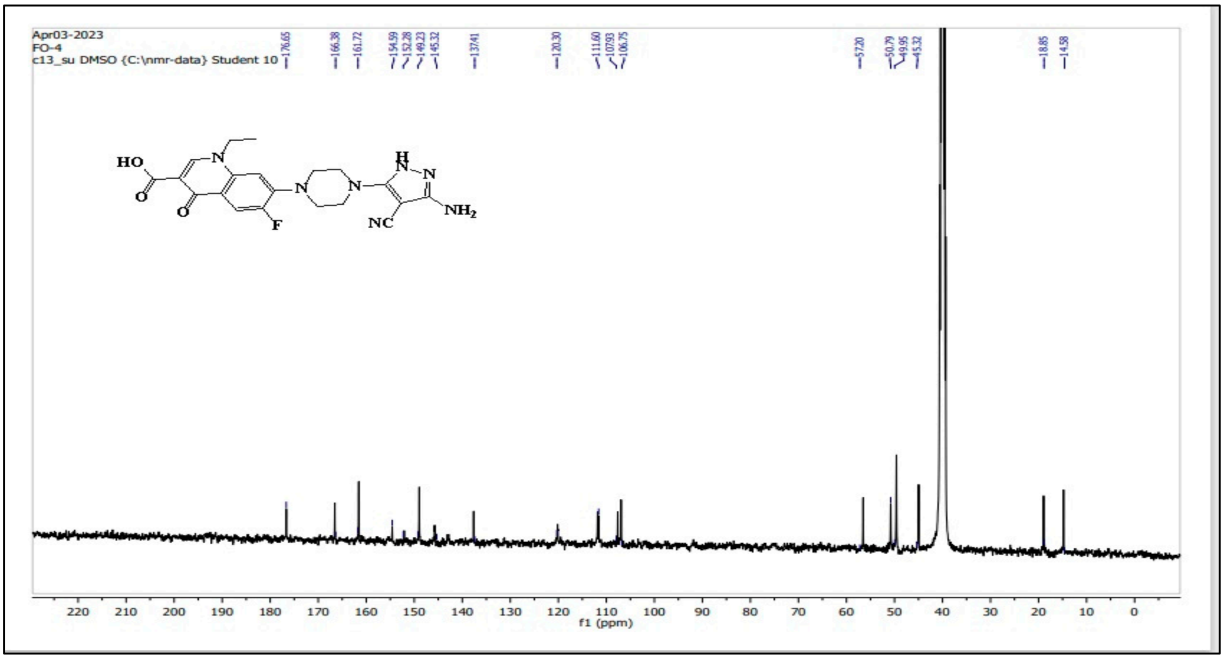

Figure S31. ^{13}CNMR spectrum of compound **13** (100 MHz, DMSO-*d6*)

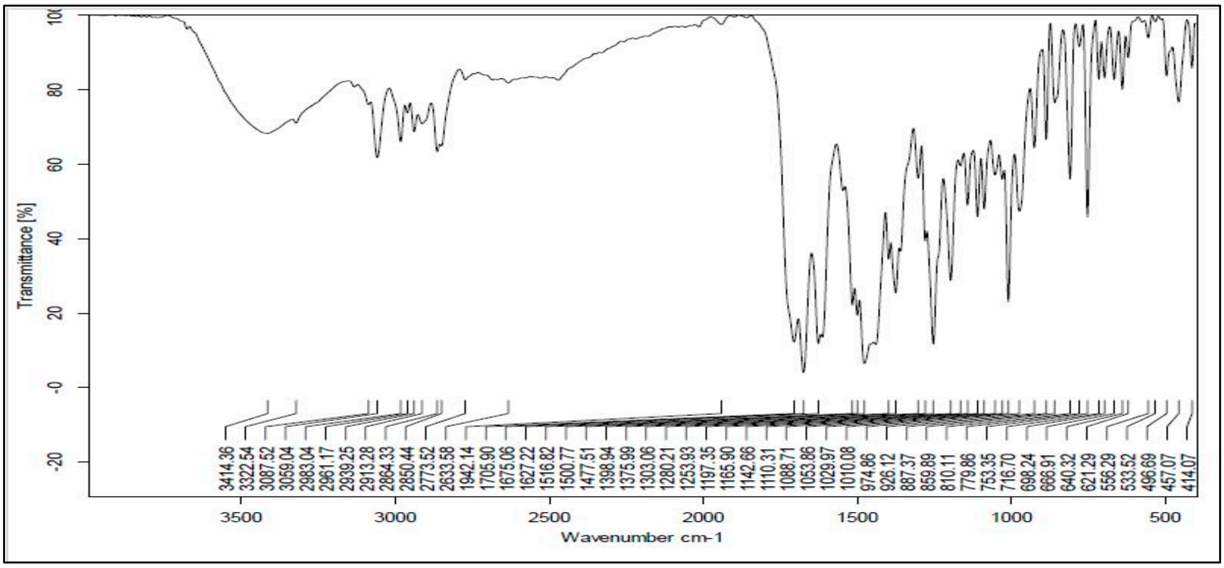

Figure S32. IR of compound **13**

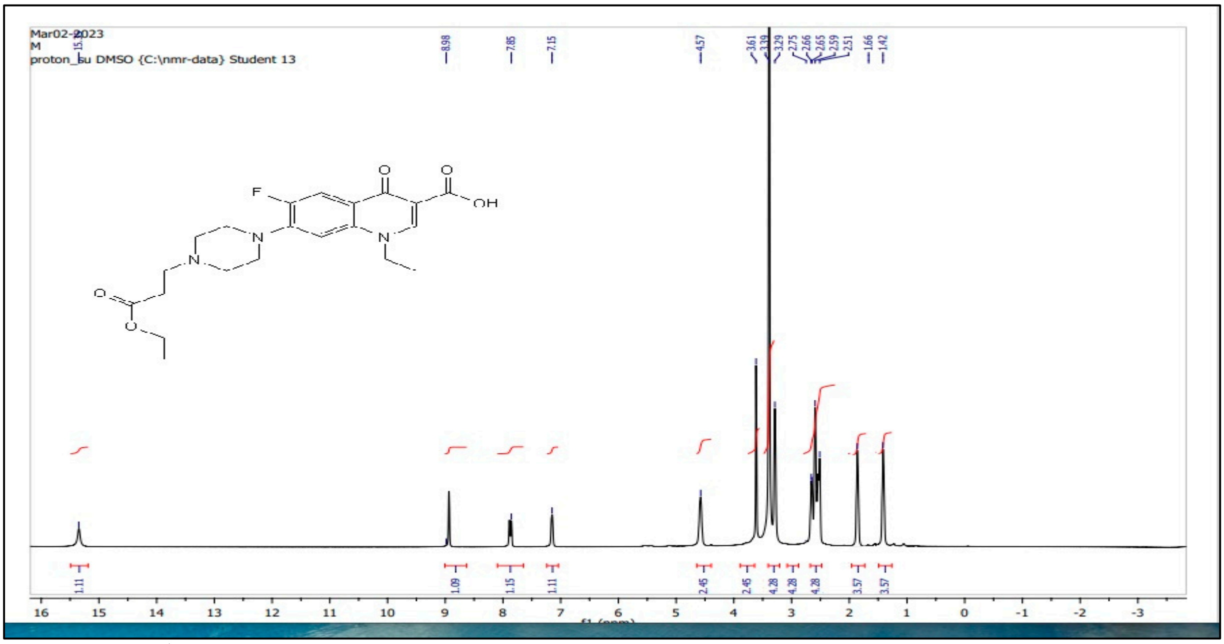

Figure S33: ^{1}HNMR spectrum of compound **14** (400 MHz, DMSO-*d6*)

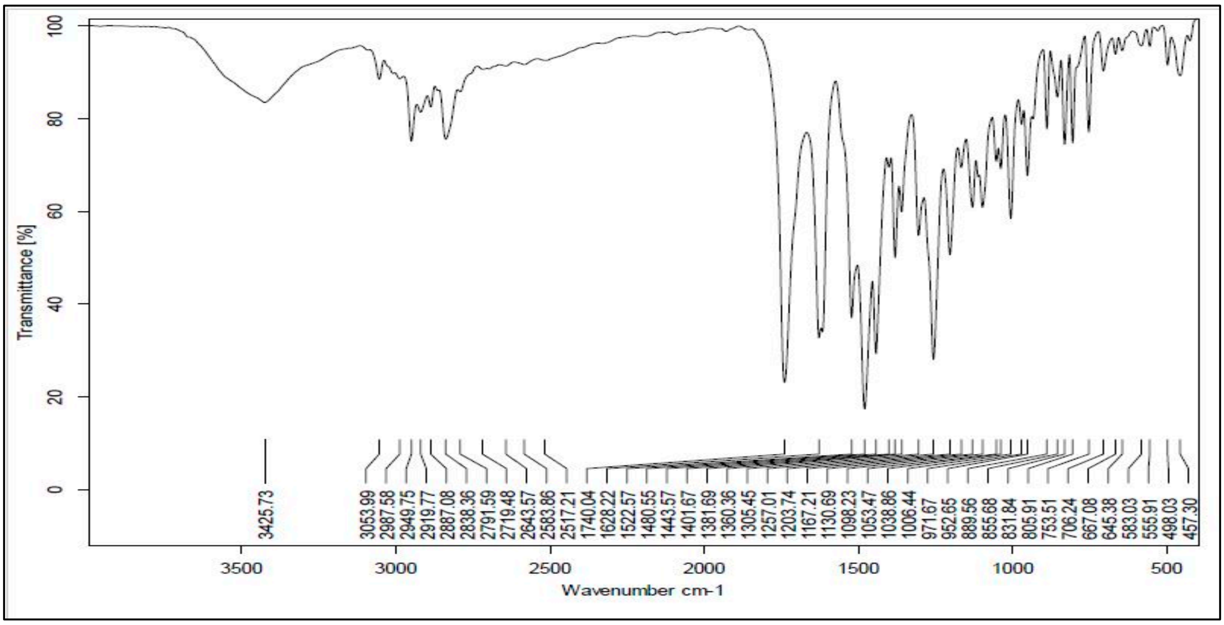

Figure S34. IR of compound **14**

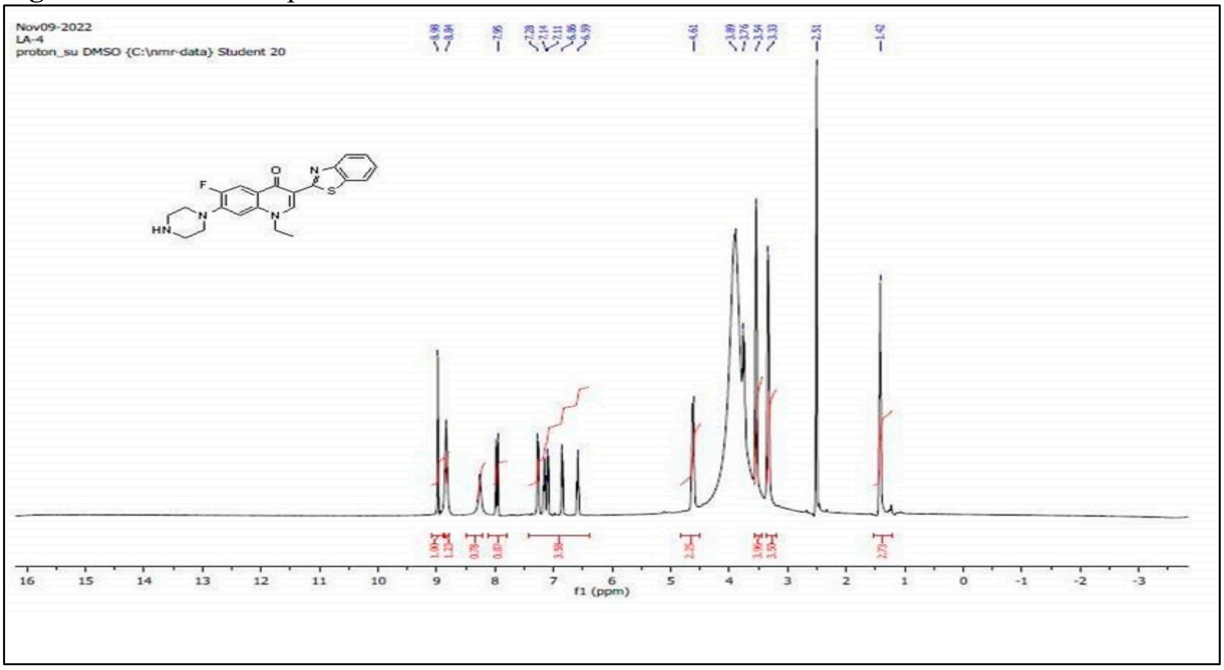

Figure S35: ^{1}HNMR spectrum of compound **15** (400 MHz, DMSO-*d6*)

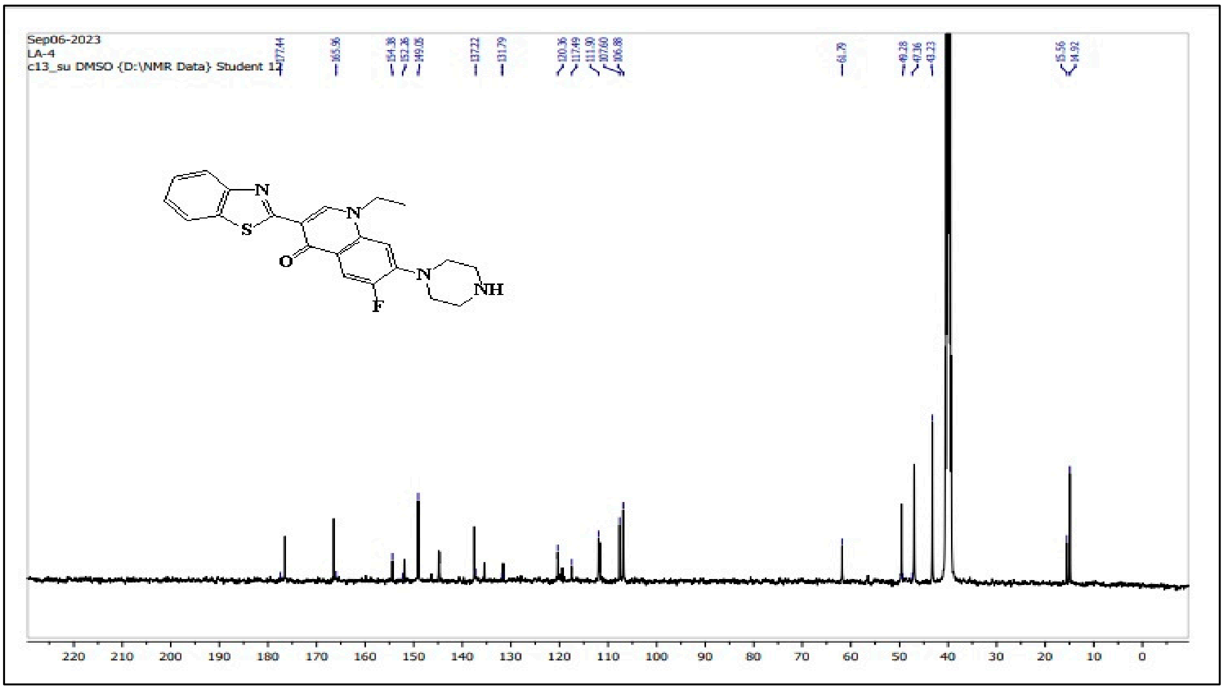

Figure S36. ^{13}CNMR spectrum of compound **15** (100 MHz, DMSO-d_6)

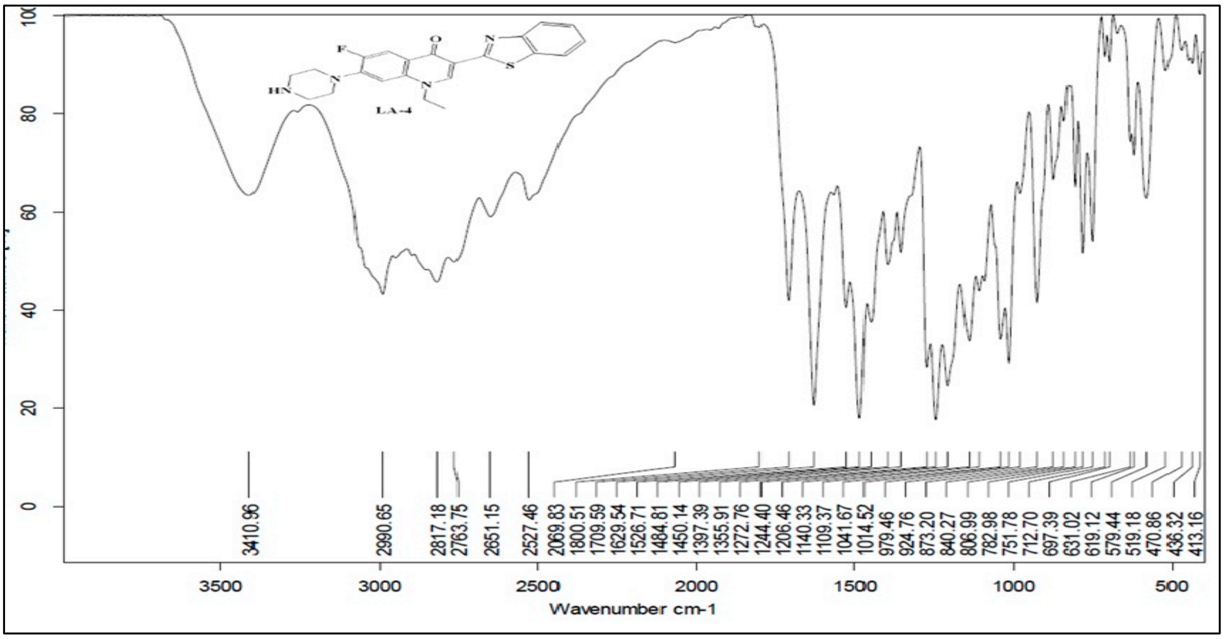

Figure S37. IR of compound **15**

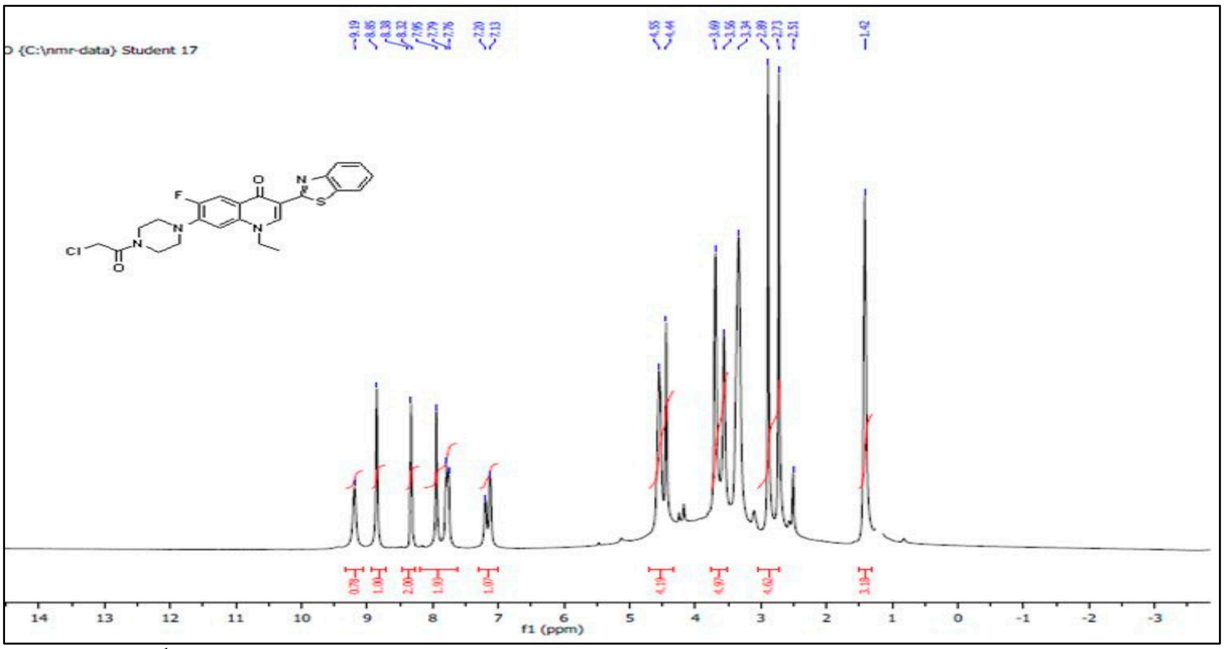

Figure S38. ^{1}HNMR spectrum of compound **16** (400 MHz, DMSO-*d6*)

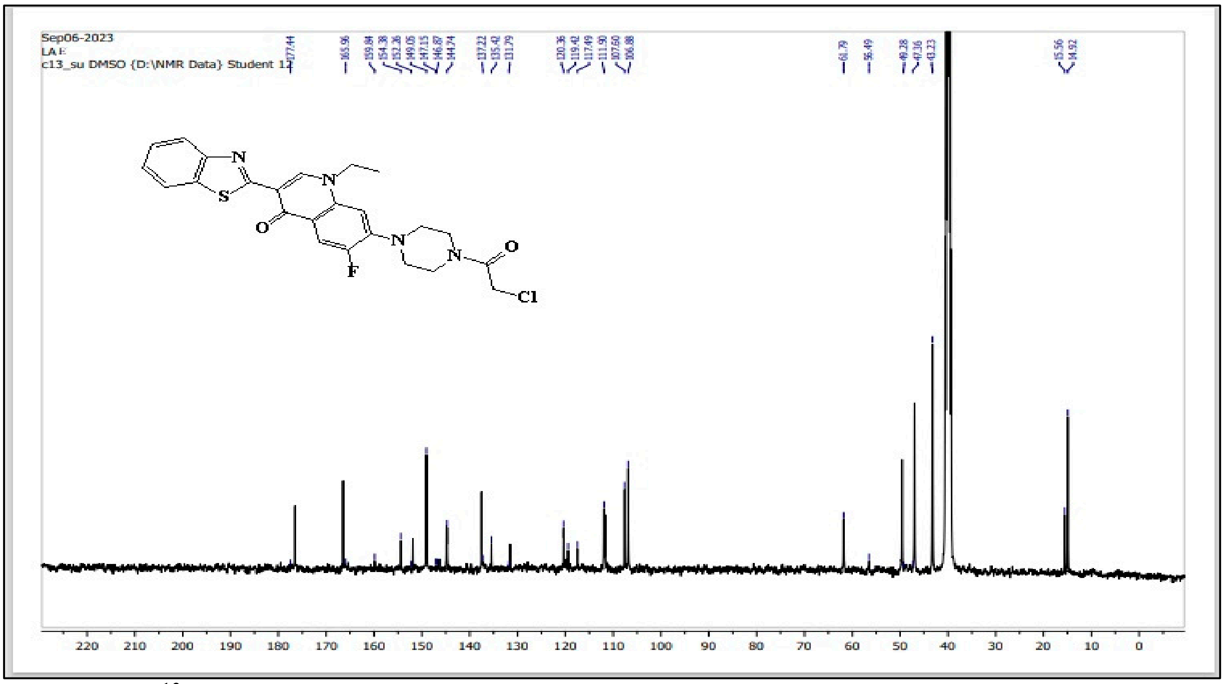

Figure S39. ^{13}CNMR spectrum of compound **16** (100 MHz, DMSO-*d*6)

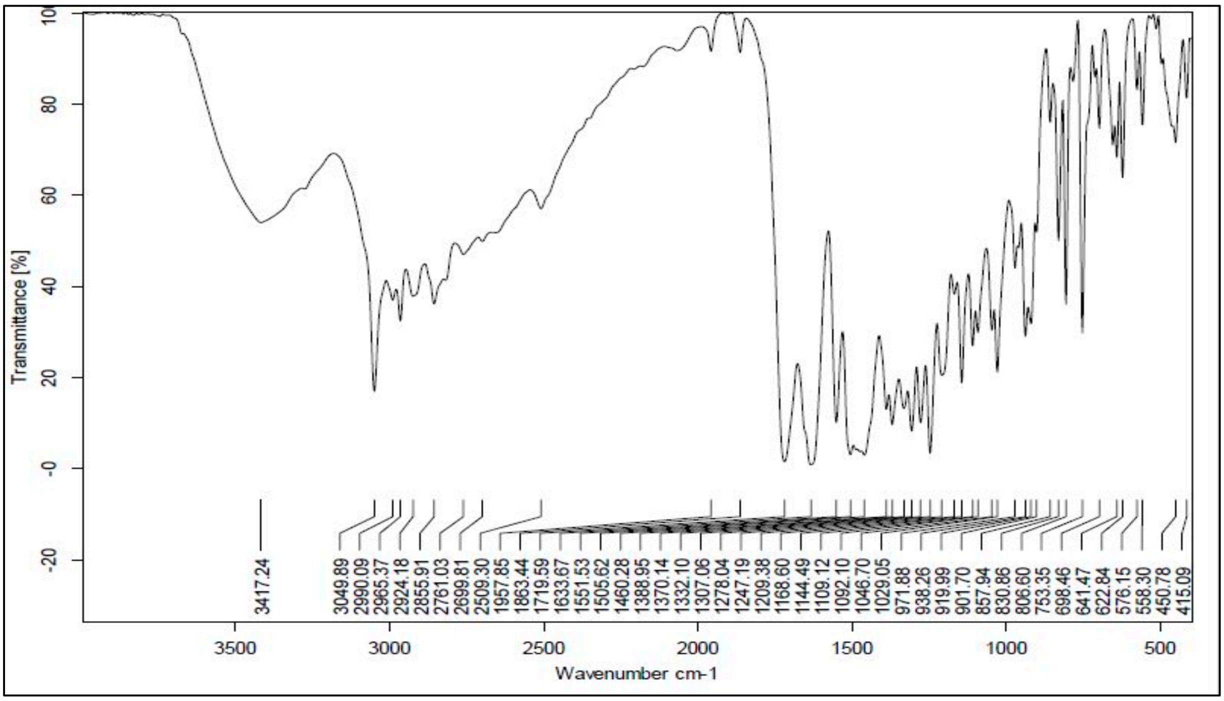

Figure S40. IR of compound **16**

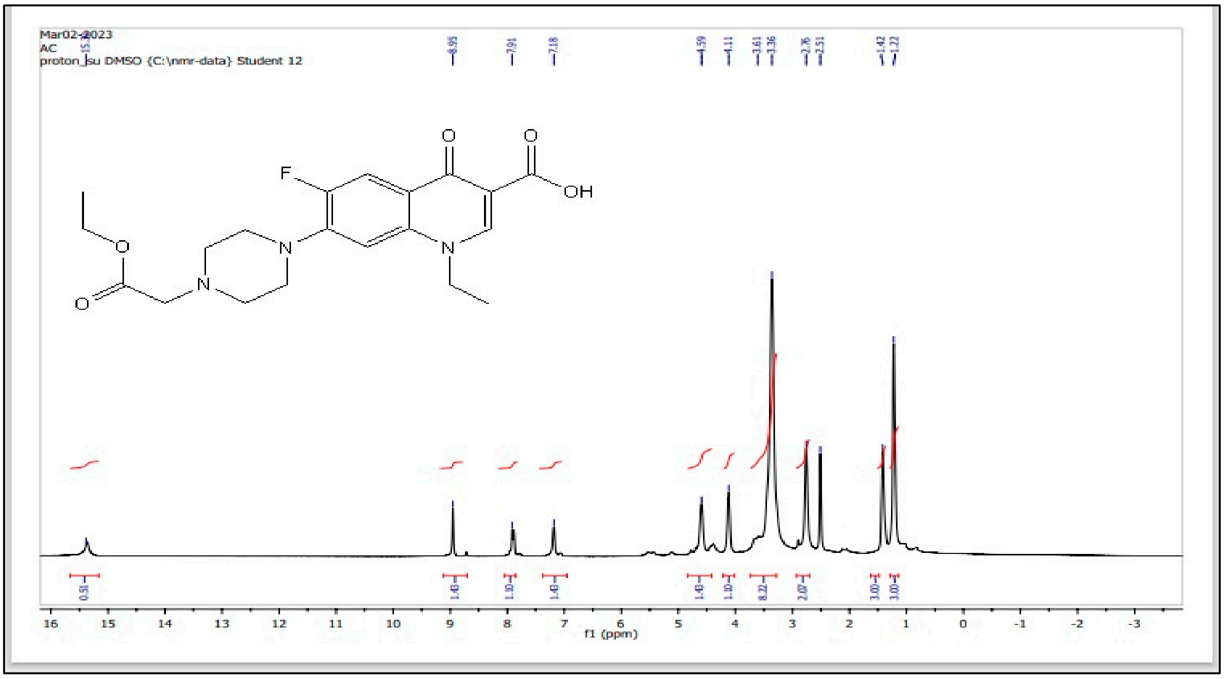

Figure S41. ^{1}HNMR spectrum of compound **17** (400 MHz, DMSO-*d6*)

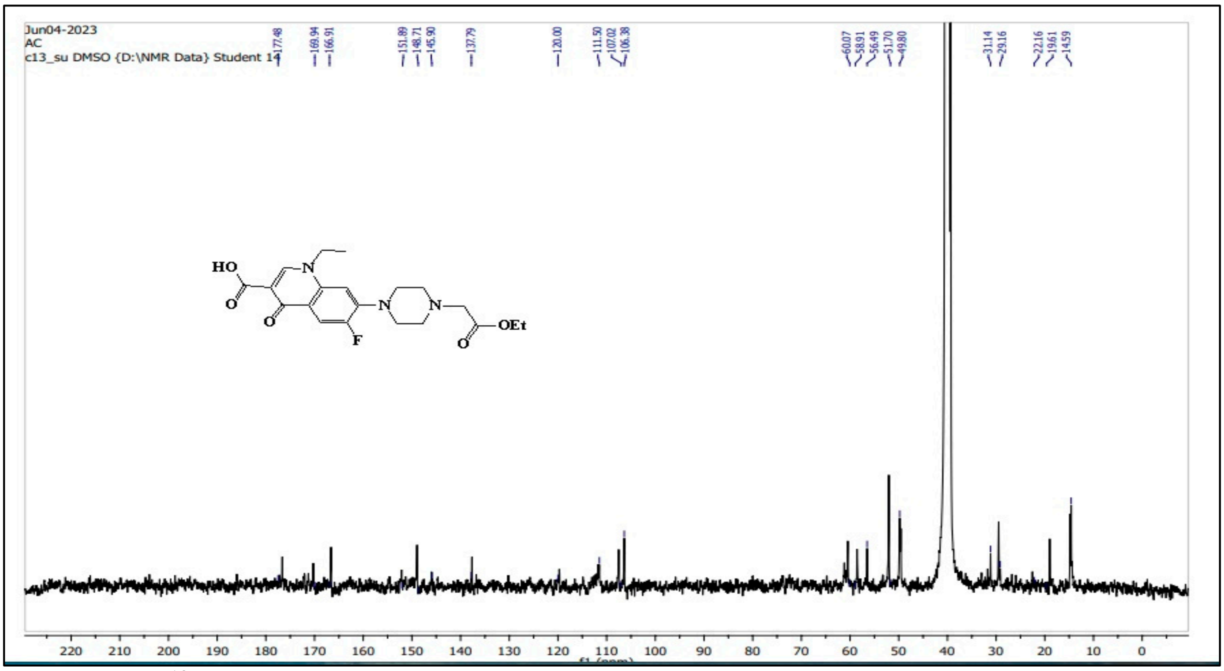

Figure S42. ^{13}CNMR spectrum of compound 17 (100 MHz, DMSO-d_6)

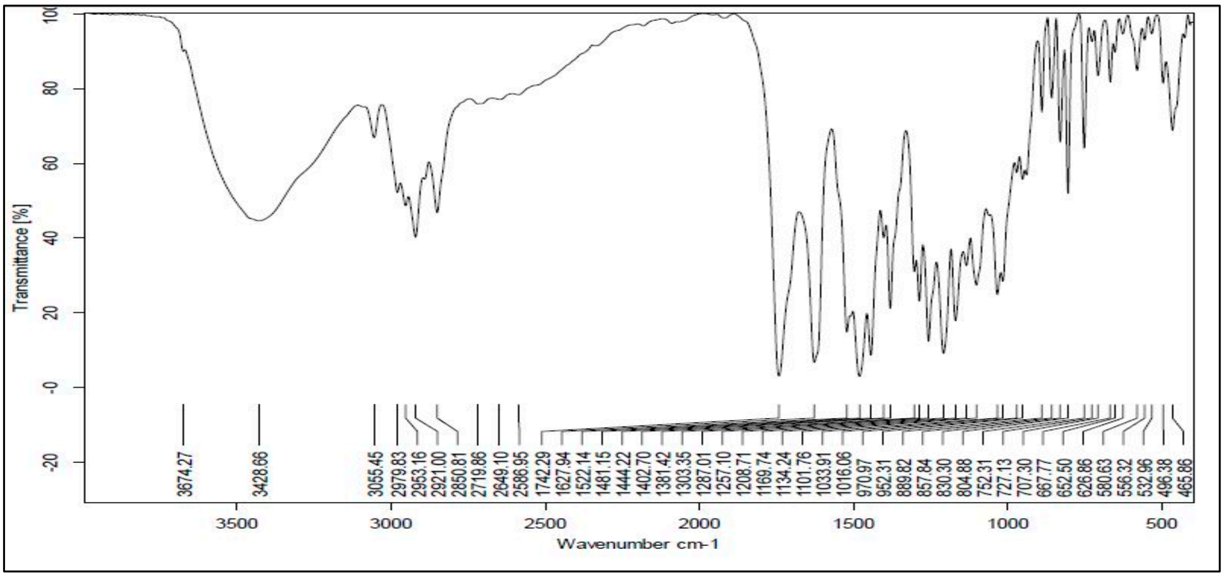

Figure S43. IR of compound **17**

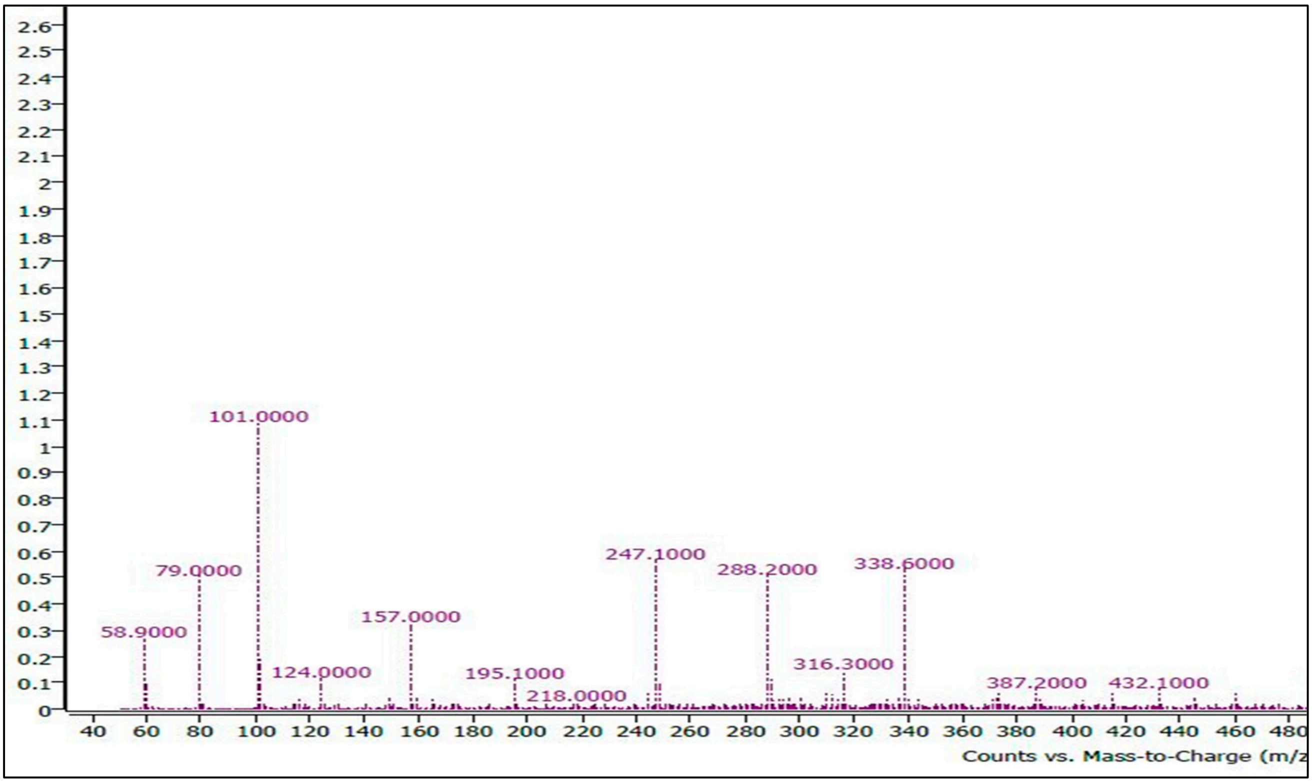

Figure S44. Mass spectrum of compound 6

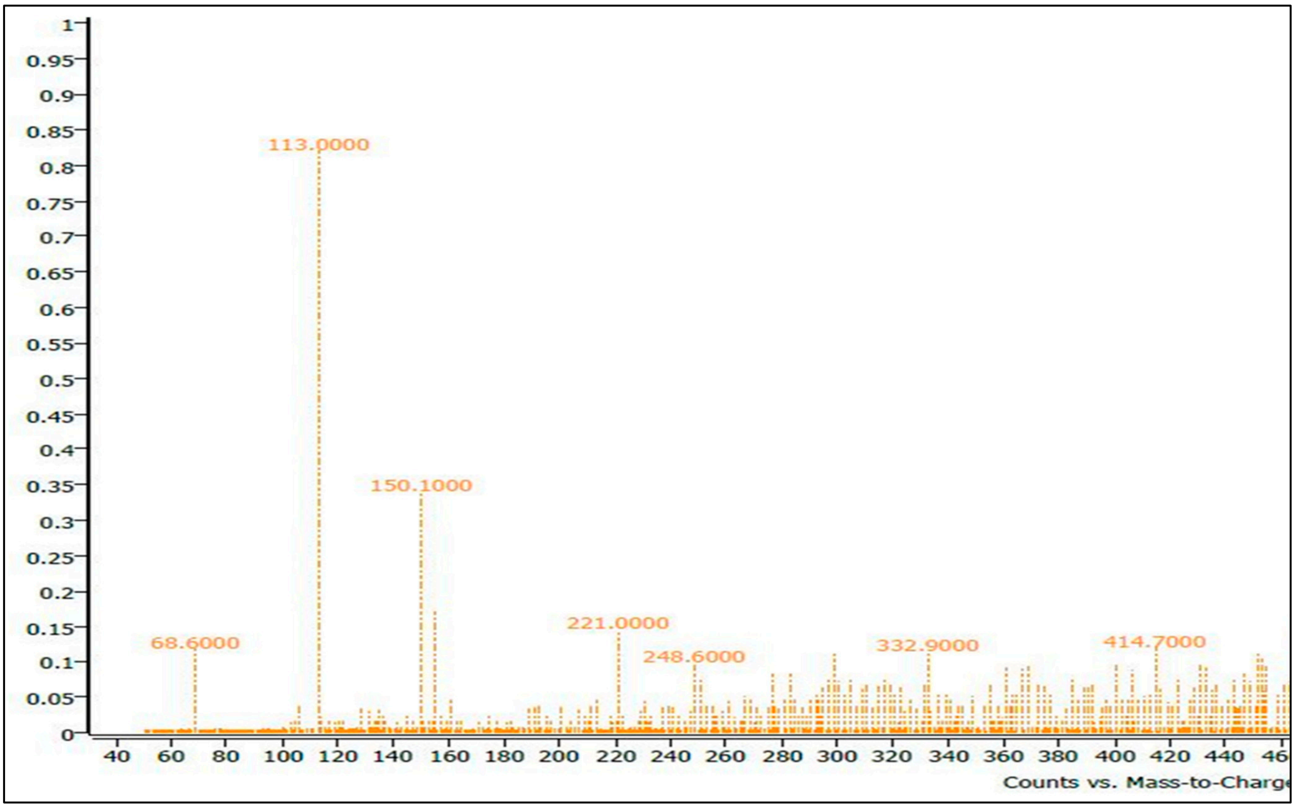

Figure S45. Mass spectrum of compound 7

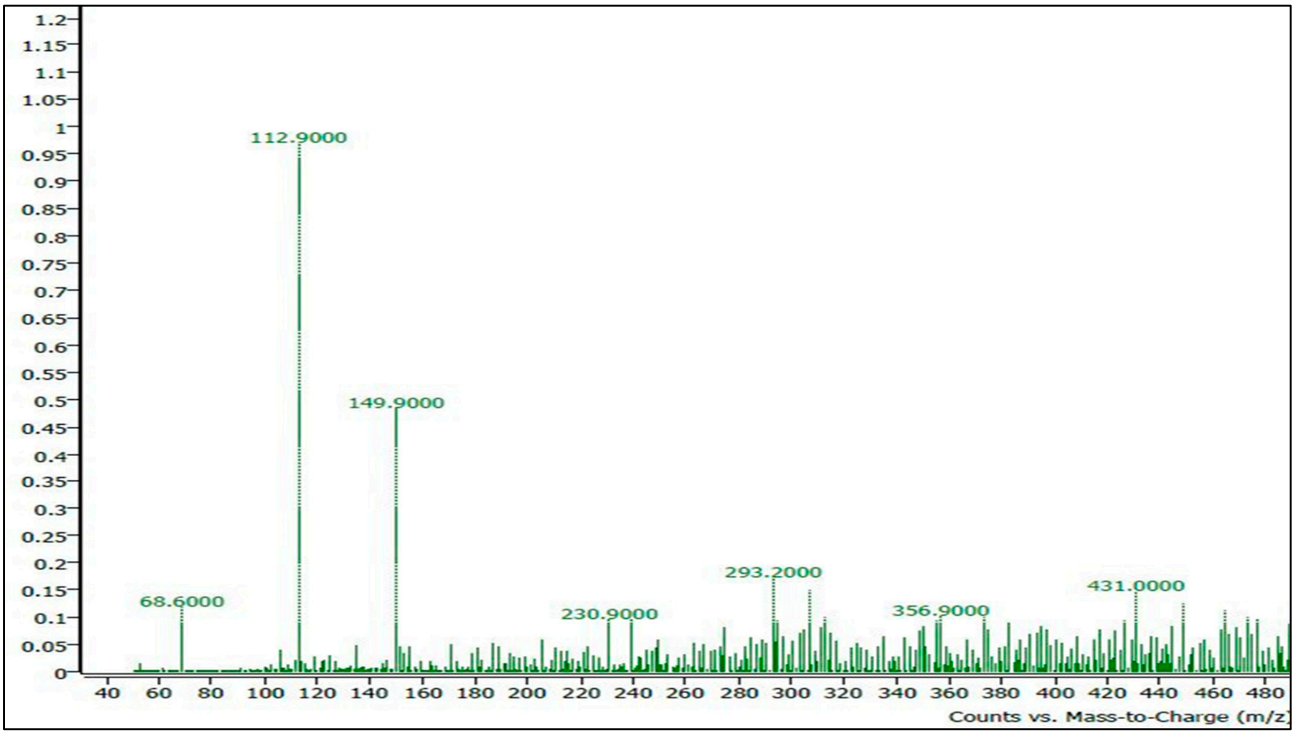

Figure S46. Mass spectrum of compound 15

2. Biology

2.1. Screening of antibacterial activity.

At the Assiut University Mycological Centre (AUMC), Faculty of Science, antibacterial activity against the Gram-negative strains *Pseudomonas aeruginosa ATCC 27853, K. pneumoniae ATCC 10031, E. coli ATCC 25922*, and Gram-positive strain *S. aureus ATCC 6538* and was assessed using the standard agar cup diffusion method.

Table S1 Results of antibacterial activity

ASSIUT UNIVERSITY MYCOLOGICAL CENTER
Report on antimicrobial activity of chemical compounds

AUMMC

Table (2): Antibacterial activity expressed as inhibition zone in mm and MICs (in µg/ml, given in brackets) of chemical compounds tested.

	Escherichia coli ATCC 25922	*Klebsiella pneumoniae* ATCC 10031	*Pseudomonas aeruginosa* ATCC 27853	*Staphylococcus aureus* ATCC 6538
1	8 (0.78)	8 (0.78)	10 (0.78)	8 (25)
2	7 (12.5)	7 (6.25)	8 (6.25)	-
3	7 (25)	7 (12.5)	8(12.5)	-
4	7 (25)	7 (6.25)	7 (6.25)	-
5	7 (25)	-	8(25)	-
6	6 (6.25)	7 (6.25)	10(3.12)	7(0.78)
7	8 (1.56)	8 (1.56)	16(3.12)	7(0.39)
8	7 (6.25)	7 (6.25)	8(3.12)	8 (6.25)
9	7 (12.5)	7 (6.25)	8(3.12)	8 (6.25)
10	10(12.5)	7 (3.12)	8(3.12)	7 (12.5)
11	9(12.5)	7 (3.12)	10(3.12)	8(12.5)
12	9(12.5)	7 (6.25)	10(12.5)	9(12.5)
13	9(12.5)	10(12.5)	12(6.25)	8 (6.25)
14	10(25)	7(12.5)	10(6.25)	8 (6.25)
15	7(25)	-	10(25)	-
16	7(12.5)	7(12.5)	8(3.12)	8 (6.25)
17	8 (1.56)	10(3.12)	8 (0.78)	10 (6.25)
18	8 (1.56)	10(3.12)	8 (1.56)	10 (6.25)
19	-	7(3.12)	7 (6.25)	8 (6.25)

Dr. Zeinab Soliman Mousa
Zeinab Soliman

5

Director of the AUMC
AH. Moharram
Prof. A. H. Moharram
14/6/2023

AUMC
ASSIUT UNIVERSITY MYCOLOGICAL CENTRE

Note 1: Compound 1 (norfloxacin), compounds 2-6 as they are, compounds 8-14 corresponding to compounds 7-13, compounds 16-19 corresponding to compounds 14-17; respectively

Note 2: the result converted to µM

Table S2 Activity of compounds **6,7, 15, 16,** and norfloxacin against *MRSA*

ASSIUT UNIVERSITY MYCOLOGICAL CENTER
Report on antimicrobial activity of chemical compounds

AUMMC

Table (2): Antibacterial activity expressed as inhibition zone in mm and MICs (in µg/ml, given in brackets) of chemical compounds tested.

	***Staphylococcus aureus* AUMC B.261(MRSA) GenbBank accession no. LC18911**
6	-
7	7(0.024)
8	7(3.12)
17	8(25)
18	-
Nor.	7(6.25)
CHL.	7(1.56)

Dr. Zeinab Soliman Mousa
Zeinab Soliman

Director of the AUMC
A.H. Moharram
Prof. A. H. Moharram
31/7/2023

ASSIUT UNIVERSITY MYCOLOGICAL CENTRE AUMC

2

Compound **Nor** is **(norfloxacin),** compounds **6, 8, 17, 18** are corresponding to compounds **6, 7, 15** and **16;** respectively.

Note that the result converted to µM

2.2. Microbial strains and culture conditions

Using strains of both Gram-positive and Gram-negative bacteria from four different bacterial species, the antibacterial activity of the synthesized compounds 2-17 was evaluated in comparison to the parent drug norfloxacin. The standard Gram-positive strain *S. aureus ATCC 6538* as well as the Gram-negative strains *E. coli ATCC 25922, K. pneumoniae ATCC 10031*, and *P. aeruginosa ATCC 27853* were given for use in this investigation by the microbiological resource center at the Faculty of Agriculture, Ain Shams University, Cairo, Egypt. All isolates were maintained at -70°C in Trypticase Soya Broth (10% glycerol, Becton and Dickinson). Prior to injection, all isolates were subcultured for 24 hours at 37°C on Trypticase Soya Agar (TSA, Becton and Dickinson) and TSB.

2.3. Determination of the minimum inhibitory concentration (MIC)

The examined bacteria were then plated using Mueller Hinton agar media (Oxoid, 20 mL) and 1 108 CFU/mL (0.5 McFarland turbidity) on sterile petri dishes. After being carefully flipped to make sure that the microorganisms were dispersed evenly, the plates were set aside to solidify on a level surface. After the material had solidified, four equally spaced circular wells with a diameter of 10 mm were painstakingly bored using a sterile cork bore. All chemicals under investigation underwent two-fold successive dilutions in order. Each dilution was added to each well three times in 100 µL increments using a micropipette. All plates underwent 24 h incubation period at 37 ºC. Following measurement, the inhibition zones' average was calculated. To find the MICs, the concentration of each diluted test chemical was plotted against the size of the corresponding zone of inhibition. The points were then connected by a regression line. The lowest concentration that prevents each strain's growth is known as the minimum inhibitory concentration, or MIC.[38-40] Three copies of each test were run on each sample.

2.4. Topoisomerase II Inhibition Assays

2.4.1. *S. aureus* Gyrase Supercoiling Assay

The *S. aureus* DNA gyrase assay was conducted in accordance with the suggested techniques collected from the literature. The tested compounds were dissolved in DMSO, serially diluted at dosages of 100, 10, 1, and 0.1M, and then tested in reaction mixtures in three different replicate runs. 40 mM HEPES, 10 mM magnesium acetate, 500 mM potassium glutamate, 2 mM ATP, 0.05 mg/mL albumin, and Relaxed pBR322 were used in a total solution volume of 30 µL. The *S. aureus* DNA gyrase was incubated at 37 ºC for 30 to 60 min. gyrase of Staph. aureus After adding 30 µL of STEB and 30 µL of chloroform/isoamyl alcohol (v:v, 24:1) to block DNA gyrase supercoiling processes, the mixture was centrifuged for one minute, and 20 µL of it was loaded on a 1% agarose gel and ran at 75 V for nearly 2 hours. 0.5 mg/L of ethidium bromide in water was used to stain the gel. Using a UV transilluminator imaging system, the fluorescent pictures were captured at a wavelength of 300 nm. Using the Imag Quant programmed (Molecular Dynamics), the fluorescence intensity of the supercoiled plasmid reaction result was quantitated. The results as IC_{50} values (concentration of the tested drug that results in 50% inhibition of enzyme activity) were computed by using nonlinear regression analysis in Graph Pad Prism.

Table S3 Screening of compounds 6, 7, 15, 16 and norfloxacin activity against Gyrase enzyme

r	Compound			DNA gyrase supercoiling	SD ±
	code	MW	conc ug	IC50 ug/ml	
1	Nor	319	---	2.282	0.092
2	Nor-Drv	417	---	0.991	0.04
3	s6	368	---	7.099	0.286
4	s7	391	---	4.065	0.164
5	s15	408	---	17.17	0.691
6	s16	484	---	3.574	0.144

Compound **s6, s7, s15, s16** and **nor** are corresponding to compounds **6, 7, 15, 16** and **norfloxacin**; respectively.

DNA Gyrase

code	IC50	conc	log	%inh
Nor		100	2	89
		50	1.7	70
		1	0	44
		0.1	-1	18
		0.01	-2	8.5
EC				0

code	IC50	conc	log	%inh
Nor-Drv		100	2	91
		50	1.7	77
		1	0	52
		0.1	-1	26
		0.01	-2	17
EC				0

code	IC50	conc	log	%inh
s6		100	2	88
		50	1.7	59
		1	0	20
		0.1	-1	8
		0.01	-2	3.6
EC				0

code	IC50	conc	log	%inh
s7		100	2	90
		50	1.7	67
		1	0	28
		0.1	-1	10
		0.01	-2	4.6
EC				0

code	IC50	conc	log	%inh
s15		100	2	82
		50	1.7	51
		1	0	7.5
		0.1	-1	3.8
		0.01	-2	1
EC				0

code	IC50	conc	log	%inh
s16		100	2	91
		50	1.7	71
		1	0	30
		0.1	-1	8.9
		0.01	-2	3.4
EC				0

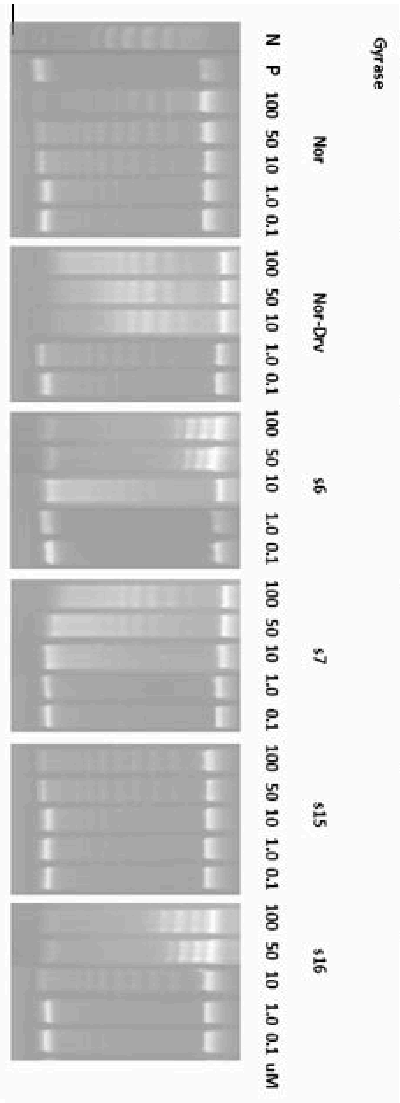

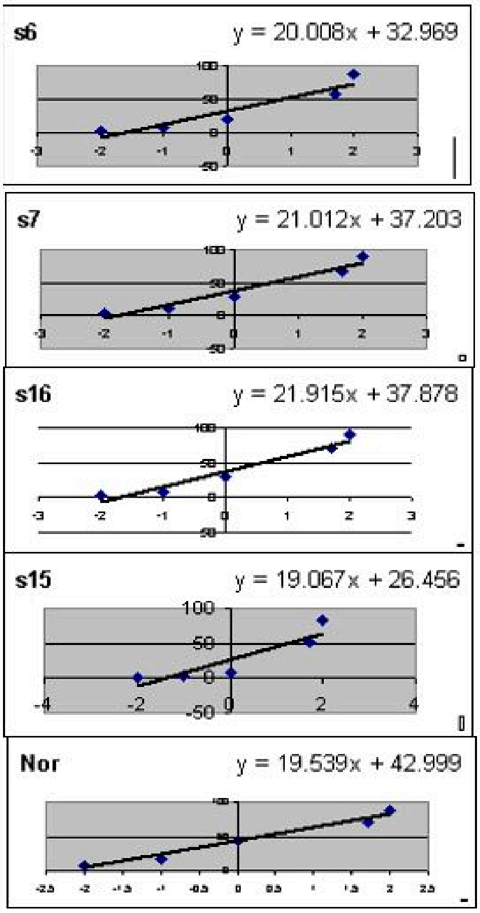

Figure S47. Screening of compounds **6, 7, 15, 16** and **norfloxacin** activity against Gyrase enzyme

Note: Compound **s6, s7, s15, s16** and nor are corresponding to compounds **6, 7, 15, 16** and **norfloxacin**; respectively.

2.4.2. *S. aureus* Topoisomerase IV Decatenation Assay

The Topo IV decatenation of the most potent newly synthesized derivatives as antibacterial agents (compounds 6, 7, 15, 16) in addition to the parent norfloxacin was evaluated, IC_{50} values were established. In this test, the following chemicals were used: Topo IV Assay Buffer (supplied as 5X), 50 mM Tris-HCl (pH 7.5), 5 mM magnesium chloride, 350 mM potassium glutamate, 5 mM DTT, and 1.5 m MATP, all of which were kept at or below 200 C, were the other ingredients. 50 mM Tris-HCl (pH 7.5), 1 mM EDTA, 1 mM DTT, and 40% (v/v) glycerol (provided as 1X) made up the dilution buffer. The substrate was the *S. aureus* Topo IV enzyme kDNA (100 ng/L). *S. aureus* Topo IV, 40% sucrose (w/v), kDNA, and 10 mM EDTA make up the STEB. The examined compounds were mixed with 100 mM Tris-HCl Bromophenol Blue, 0.5 mg/mL, pH 8, and different quantities. The mixes were incubated at 37 ºC for 30 min. The responses were stopped using STEB. The effects of the procedure were examined using agarose gel electrophoresis. After that, the gel was coloured with ethidium bromide before being photographed with UV light.

Table S4 Screening of compounds **6, 7, 15, 16**, and **norfloxacin** activity against topo IV

ser	Compound			TOP IV	SD
	code	MW	conc ug	IC50 uM	±
1	Nor	319	---	11.92	0.48
2	Nor-Drv	417	---	3.424	0.138
3	s6	368	---	3.006	0.121
4	s7	391	---	2.406	0.097
5	s15	408	---	9.987	0.402
6	s16	484	---	1.914	0.077

Compound **s6, s7, s15, s16** and **nor** are corresponding to compounds **6, 7, 15, 16** and **norfloxacin**; respectively

TOPO IV				
code	IC50	conc	log	%inh
Nor		100	2	83
		50	1.7	55
		1	0	13
		0.1	-1	7.4
		0.01	-2	1.8
EC				0
code	IC50	conc	log	%inh
Nor-Drv		100	2	85
		50	1.7	61
		1	0	21
		0.1	-1	7.5
		0.01	-2	3
EC				0
code	IC50	conc	log	%inh
s6		100	2	91
		50	1.7	71
		1	0	35
		0.1	-1	12
		0.01	-2	5.7
EC				0
code	IC50	conc	log	%inh
s7		100	2	90
		50	1.7	72
		1	0	37
		0.1	-1	18
		0.01	-2	8.2
EC				0
code	IC50	conc	log	%inh
s15		100	2	84
		50	1.7	56
		1	0	16
		0.1	-1	7.8
		0.01	-2	3.4
EC				0
code	IC50	conc	log	%inh
s16		100	2	89
		50	1.7	80
		1	0	38
		0.1	-1	20
		0.01	-2	8.7
EC				0

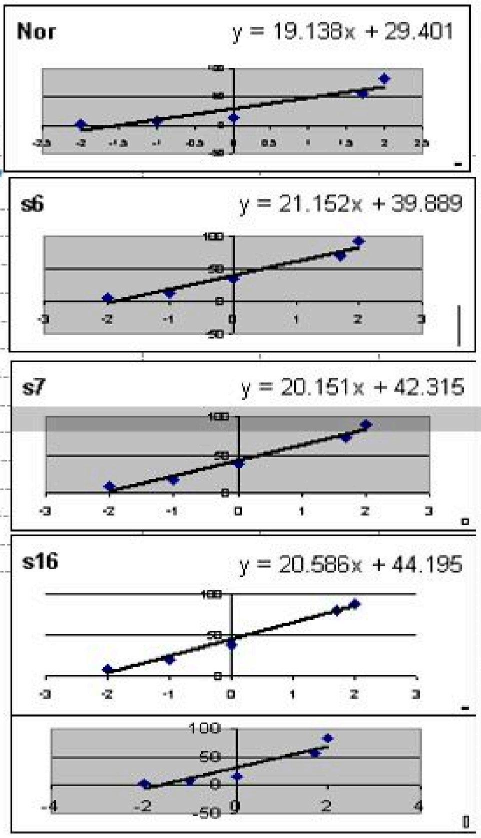

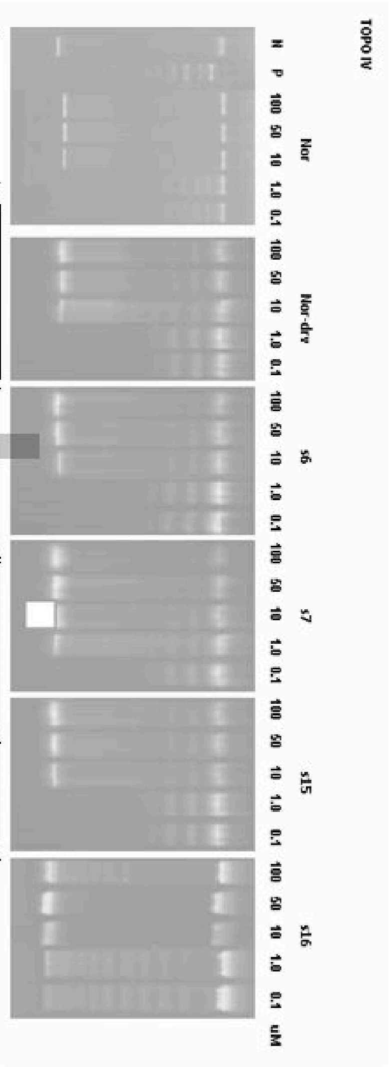

Figure S48. Screening of compounds **6, 7, 15, 16** and **norfloxacin** activity against topo IV
Note: Compound **s6, s7, s15, s16** and **nor** are corresponding to compounds **6, 7, 15, 16** and **norfloxacin**; respectively

2.5. Cytotoxicity assay

The American Type Culture Collection provided the cell line cells, which were grown in DMEM (Invitrogen/Life Technologies) with 10% FBS (Hyclone), 10 ug/ml of insulin (Sigma), and 1% penicillin-streptomycin. The remaining substances and tools were from either Sigma or Invitrogen. Before performing the MTT experiment, seed cells on a 96-well plate at a density of 1.2–1.8 10,000 cells/well in a volume of 100 µL complete growth media + 100 µL of the test substance each well. Multiwell plates are an excellent choice for using the MTT method, which is used to track in vitro cytotoxicity. Using cells in the log phase of growth and keeping the ultimate cell density around 106 cells/cm2 will produce the best results. Every test should include a blank with a full medium but no cells. Cultures should be moved from the incubator into a sterile work space such as a laminar flow hood. Each MTT [M-5655] vial should be reconstituted with 3 ml of medium or balanced salt solution devoid of phenol red and serum. 10% of the volume of the culture medium should be added to the reconstituted MTT solution. Incubate cultures again for 2-4 h, depending on the type of cell and the maximal cell density. (A 2-hours incubation period is usually sufficient, although it can be extended for cells with low densities or low metabolic activity.) When comparing incubation periods, they should be comparable. After the incubation period, remove the cultures from the incubator and apply MTT Solubilization Solution [M-8910] in a quantity equal to the volume of the original culture media to dissolve the formazan crystals that formed. In a gyratory shaker, gentle blending will improve solubility. Trituration, or pipetting up and down, may occasionally be necessary to completely dissolve the MTT formazan crystals, especially in thick cultures. at a wavelength of 450 nm, measure absorbance spectrophotometrically. Subtract from the 450 nm data the background absorbance of multiwell plates at 690 nm. A suitable plate reader can be used to read tests conducted on multiwell plates, or the contents of individual wells can be transferred to cuvettes of the right size for spectrophotometric analysis.

Table S5 Cytotoxicity screening of compounds 6, 7, 15, 16 and norfloxacin against normal cell line W138

Ser	Sample			Cytotoxicity IC50 uM	SD ±
	code	MW	cells	WI38	
1	Nor	319	WI38	54.57	1.94
2	Nor-Drv	417	---	89.51	3.19
3	s6	368	---	34.06	1.21
4	s7	391	---	47.92	1.71
5	s15	408	---	39.16	1.39
6	s16	484	---	51.24	1.82
***	Doxorubicin	543.52	---	14.11	0.5

Compound **s6, s7, s15, s16** and **nor** are corresponding to compounds **6, 7, 15, 16** and **norfloxacin**; respectively

researcher	assay	Date	cells
Dr.Hossam Aziz	MTT	17-Sep	WI38

	Blank	CC	Sample No.		Nor/WI38			Sample No.		Nor-Drv/WI38		
	1	2	3	4	5	6	7	8	9	10	11	12
A	B	C	100uM	25uM	6.3uM	1.6uM	0.4uM	100uM	25uM	6.3uM	1.6uM	0.4uM
B	B	C	100uM	25uM	6.3uM	1.6uM	0.4uM	100uM	25uM	6.3uM	1.6uM	0.4uM
C	B	C	100uM	25uM	6.3uM	1.6uM	0.4uM	100uM	25uM	6.3uM	1.6uM	0.4uM

ROBONIK P2000 eia reader

Wave length: 450 nm

Reference: 630 nm

	1	2	3	4	5	6	7	8	9	10	11	12
A	0.001	0.538	0.228	0.303	0.371	0.424	0.466	0.266	0.322	0.376	0.449	0.539
B	0.001	0.529	0.241	0.325	0.366	0.419	0.457	0.274	0.337	0.392	0.462	0.541
C	0.001	0.544	0.233	0.292	0.354	0.427	0.461	0.261	0.341	0.385	0.458	0.528
mean	4E-04	0.537	0.234	0.307	0.3637	0.4233	0.4613	0.267	0.3333	0.3843	0.4563	0.536
%		`	43.575	57.11	67.722	78.833	85.909	49.721	62.073	71.57	84.978	99.814

Nor/WI38

log conc.	% viability
2	43.58
1.398	57.11
0.796	67.72
0.193	78.83
-0.409	85.91

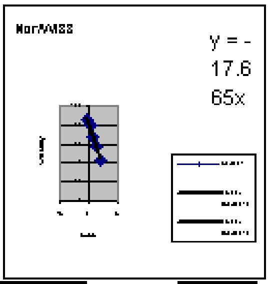

IC50= [redacted] [redacted] [redacted]

Nor-Drv/WI38

log conc.	% viability
2	49.721
1.3979	62.073
0.7959	71.57
0.1931	84.978
-0.409	99.814

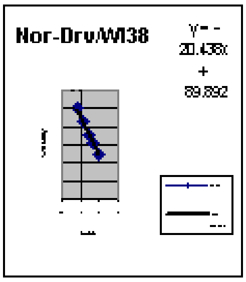

IC50= [redacted] [redacted]

	Blank	CC	Sample No.		s6/WI38			Sample No.		s7/WI38		
	1	2	3	4	5	6	7	8	9	10	11	12
A	B	C	100uM	25uM	6.3uM	1.6uM	0.4uM	100uM	25uM	6.3uM	1.6uM	0.4uM
B	B	C	100uM	25uM	6.3uM	1.6uM	0.4uM	100uM	25uM	6.3uM	1.6uM	0.4uM
C	B	C	100uM	25uM	6.3uM	1.6uM	0.4uM	100uM	25uM	6.3uM	1.6uM	0.4uM

ROBONIK P2000 eia reader

Wave length: 450 nm

Reference: 630 nm

	1	2	3	4	5	6	7	8	9	10	11	12
A	0.001	0.533	0.209	0.281	0.353	0.425	0.465	0.223	0.295	0.346	0.414	0.449
B	0.001	0.525	0.214	0.277	0.346	0.441	0.462	0.218	0.311	0.361	0.395	0.452
C	0.001	0.519	0.196	0.273	0.338	0.427	0.471	0.228	0.285	0.359	0.418	0.477
mean	0.001	0.526	0.2063	0.277	0.3457	0.431	0.466	0.223	0.297	0.3553	0.409	0.4593

% viability			39.252	52.69	65.758	81.991	88.649	42.422	56.5	67.597	77.806	87.381

s6/WI38

log conc.	% viability
2	39.25
1.398	52.69
0.796	65.76
0.193	81.99
-0.409	88.65

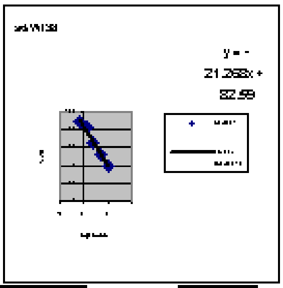

IC50= [redacted]

s7/WI38

log conc.	% viability
2	42.422
1.3979	56.5
0.7959	67.597
0.1931	77.806
-0.409	87.381

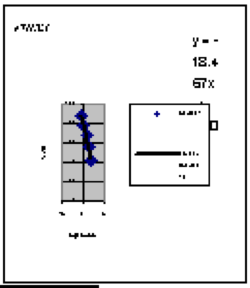

IC50= [redacted]

	Blank	CC	Sample No.		s15/WI38			Sample No.		s16/WI38		
	1	2	3	4	5	6	7	8	9	10	11	12
A	B	C	100uM	25uM	6.3uM	1.6uM	0.4uM	100uM	25uM	6.3uM	1.6uM	0.4uM
B	B	C	100uM	25uM	6.3uM	1.6uM	0.4uM	100uM	25uM	6.3uM	1.6uM	0.4uM
C	B	C	100uM	25uM	6.3uM	1.6uM	0.4uM	100uM	25uM	6.3uM	1.6uM	0.4uM

ROBONIK P2000 eia reader

Wave length: 450 nm

Reference: 630 nm

	1	2	3	4	5	6	7	8	9	10	11	12
A	0.001	0.492	0.193	0.261	0.311	0.369	0.422	0.221	0.276	0.355	0.424	0.464
B	0.003	0.515	0.225	0.273	0.327	0.372	0.416	0.208	0.291	0.346	0.418	0.475
C	0.001	0.477	0.221	0.259	0.304	0.381	0.441	0.202	0.288	0.338	0.411	0.459
mean	0.002	0.495	0.213	0.264	0.314	0.374	0.4263	0.2103	0.285	0.3463	0.4177	0.466
% viability			43.059	53.44	63.477	75.606	86.186	42.52	57.615	70.013	84.434	94.205

s15/WI38

2	43.06
1.398	53.44
0.796	63.48
0.193	75.61
-0.409	86.19

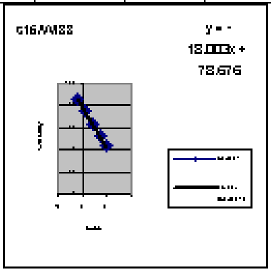

IC50= [redacted]

s16/WI38

2	42.52
1.3979	57.615
0.7959	70.013
0.1931	84.434
-0.409	94.205

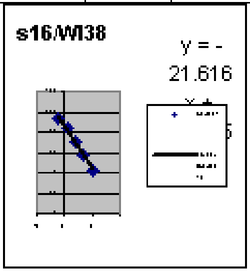

IC50= [redacted]

	Blank	CC	Sample No.			Dox/WI38		Sample No.				
	1	2	3	4	5	6	7	8	9	10	11	12
A	B	C	100uM	25uM	6.3uM	1.6uM	0.4uM					
B	B	C	100uM	25uM	6.3uM	1.6uM	0.4uM					
C	B	C	100uM	25uM	6.3uM	1.6uM	0.4uM					

ROBONIK P2000 eia reader

Wave length: 450 nm

Reference: 630 nm

	1	2	3	4	5	6	7	8	9	10	11	12
A	0.001	0.537	0.191	0.262	0.298	0.339	0.384					
B	0.001	0.544	0.176	0.255	0.312	0.347	0.389					
C	0.001	0.529	0.193	0.259	0.319	0.341	0.385					
mean	0.001	0.537	0.1867	0.259	0.3097	0.3423	0.386	0	0	0	0	0
% viability			34.783	48.2	57.702	63.789	71.925	0	0	0	0	0

Dox/WI38

log conc.	% viability
2	34.78
1.398	48.2
0.796	57.7
0.193	63.79
-0.409	71.93

IC50=

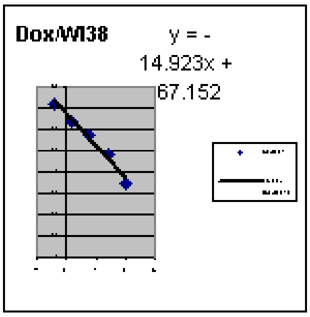

log conc.	% viability
2	0
1.3979	0
0.7959	0
0.1931	0
-0.409	0

IC50=

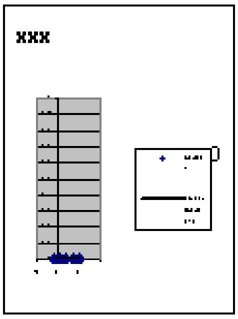

SH-BZ-2/HCT116	y = -17.665x + 80.684
Nor-Drv/WI38	y = -20.438x + 89.892
s6/WI38	y = -21.268x + 82.59
s7/WI38	y = -18.467x + 81.034
s15/WI38	y = -18.003x + 78.676
s16/WI38	y = -21.616x + 86.955
Dox/WI38	y = -14.923x + 67.152

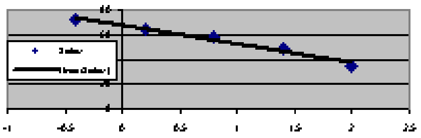

Figure S49. Cytotoxicity screening of compounds **6, 7, 15, 16,** and **norfloxacin** against normal cell line W138

Note: Compound **s6, s7, s15, s16,** and **nor** are corresponding to compounds **6, 7, 15, 16**, and **norfloxacin**; respectively

Assay Protocol

Cell Line cells were obtained from American Type Culture Collection, cells were cultured using DMEM (Invitrogen/Life Technologies) supplemented with 10% FBS (Hyclone,), 10 ug/ml of insulin (Sigma), and 1% penicillin-streptomycin. All of the other chemicals and reagents were from Sigma, or Invitrogen.
Plate cells (cells density 1.2 – 1.8 × 10,000 cells/well) in a volume of 100µl complete growth medium + 100 ul of the tested compound per well in a 96-well plate for 24 hours before the MTT assay.

Cell cuture protocol
1. Remove culture medium to a centrifuge tube.
2. Briefly rinse the cell layer with 0.25% (w/v) Trypsin 0.53 mM EDTA solution to remove all traces of serum which contains Trypsin inhibitor.
3. Add 2.0 to 3.0 ml of Trypsin EDTA solution to flask and observe cells under an inverted microscope until cell layer is dispersed (usually within 5 to 15 minutes).
Note: To avoid clumping do not agitate the cells by hitting or shaking the flask while waiting for the cells to detach. Cells that are difficult to detach may be placed at 37°C to facilitate dispersal.
4. Add 6.0 to 8.0 mL of complete growth medium and aspirate cells by gently pipetting.
5. Transfer the cell suspension to the centrifuge tube with the medium and cells from step 1, and centrifuge at approximately 125 xg for 5 to 10 minutes. Discard the supernatant.
6. Resuspend the cell pellet in fresh growth medium. Add appropriate aliquots of the cell suspension to new culture vessels.
7. Incubate cultures at 37°C for 24 hrs.

8-After treatment of cells with the serial concentrations of the compound to be tested incubation is carried out for 48 h at 37ºC ,then the plates are to be examined under the inverted microscope and proceed for the MTT assay

MTT – Cytotoxicity assay protocol

The MTT method of monitoring in vitro cytotoxicity is
well suited for use with multiwell plates. For best results, cells in the log phase of growth should be employed and final cell number should not exceed 106 cells/cm2. Each test should include a blank containing complete medium without cells.

1. Remove cultures from incubator into laminar
flow hood or other sterile work area.

2. Reconstitute each vial of MTT [M-5655] to be used with 3 ml of medium or balanced salt solution without phenol red and serum. Add reconstituted MTT in an amount equal to 10% of the culture medium volume.

3. Return cultures to incubator for 2-4 hours depending on cell type and maximum cell density. (An incubation period of 2 hours is generally adequate but may be lengthened for low cell densities or cells with lower metabolic activity.) Incubation times should beconsistent when making comparisons.

4. After the incubation period, remove cultures from incubator and dissolve the resulting formazan crystals by adding an amount of MTT Solubilization Solution [M-8910] equal to the original culture medium volume.

5. Gentle mixing in a gyratory shaker will enhance dissolution. Occasionally, especially in dense cultures, pipetting up and down [trituration] may be required to completely dissolve the MTT formazan crystals.
6. Spectrophotometrically measure absorbance
at a wavelength of 450 nm. Measure the background absorbance of multiwell plates at 690 nm and subtract from the 450 nm measurement. Tests performed in multiwell plates can be read using the appropriate type of plate
reader or the contents of individual wells may be transferred to appropriate size cuvets for spectrophotometric measurements.

2.6. In silico studies

2.6.1. Docking Studies

From the Protein Data Bank, the structure of bacterial DNA gyrase (PDB code: 2XCT) was retrieved.

Physicochemical and pharmacokinetic prediction

Table S6. Physicochemical properties of target compounds **2-17** and norfloxacin.

Molecule	#Heavy atoms	#Aromatic heavy atoms	Fraction Csp3	#Rotatable bonds	#HBAs	#HBDs	MR	TPSA
**2**	27	10	0.39	5	5	1	107.26	82.85
**3**	28	10	0.39	6	7	3	107.97	120.9
**4**	35	16	0.28	8	6	2	138.24	107.24
**5**	40	16	0.29	7	7	3	159.57	147.93
**6**	28	10	0.37	5	6	1	107.02	106.64
**7**	28	10	0.42	6	6	1	108.74	92.08
**8**	27	10	0.35	5	6	3	103.55	120.9
**9**	25	10	0.35	4	5	1	98.04	82.85
**10**	26	10	0.35	4	5	2	103.85	104.16
**11**	32	16	0.26	6	5	2	129.97	90.17
**12**	29	10	0.3	4	6	1	110.9	113.36
**13**	31	15	0.3	4	6	3	118.96	144.27
**14**	30	10	0.48	8	7	1	117.97	92.08
**15**	29	19	0.27	3	4	1	124.21	78.4
**16**	33	19	0.29	5	4	0	138.91	86.68
**17**	29	10	0.45	7	7	1	113.16	92.08
Norfloxacin	23	10	0.38	3	5	2	92.55	74.57

Table S7. Lipophilicity parameters of target compounds **2-17** and norfloxacin.

Molecule	iLOGP	XLOGP3	WLOGP	MLOGP	Silicos-IT Log P	Consensus Log P
**2**	2.11	1.8	1.4	1.28	2.21	1.76
**3**	1.85	-2.25	-0.37	0.29	0.08	-0.08
**4**	2.54	2.96	1.79	1.52	2.79	2.32
**5**	3.04	0.5	0.34	0.93	0.98	1.16
**6**	2.16	1.06	1.08	0.41	1.61	1.26
**7**	3.02	1.95	1.8	1.3	1.63	1.94
**8**	1.3	-2.02	0.22	0.87	-0.31	0.01
**9**	2.24	1.19	0.8	1.22	1.46	1.38
**10**	1.57	1.12	0.54	1.24	0.93	1.08
**11**	2.53	3.34	2.5	2.36	2.44	2.63
**12**	1.85	1.73	1.57	0.16	1.65	1.39
**13**	1.99	2.05	1.03	0.47	1.1	1.33
**14**	2.5	0.46	1.59	1.34	2.43	1.66
**15**	3.65	3.09	3.51	2.65	5.02	3.58

**16**	3.57	4.31	3.98	2.8	5.4	4.01
**17**	2.93	0.56	1.2	1.12	2.03	1.57
Norfloxacin	2.16	-1.03	0.93	1.04	1.82	0.98

Table S8. Water solubility parameters of target compounds **2-17** and norfloxacin.

Molecule	ESOL Solubility (mg/ml)	ESOL Class	Ali Solubility (mg/ml)	Ali Class	Silicos-IT Solubility (mg/ml)	Silicos-IT class
**2**	1.68E-01	Soluble	2.75E-01	Soluble	4.93E-02	Soluble
**3**	7.50E+01	Very soluble	6.88E+02	Highly soluble	4.05E-01	Soluble
**4**	1.56E-02	Moderately soluble	6.40E-03	Moderately soluble	6.48E-04	Moderately soluble
**5**	2.32E-01	Soluble	3.63E-01	Soluble	2.53E-03	Moderately soluble
**6**	5.62E-01	Soluble	4.97E-01	Soluble	1.60E-01	Soluble
**7**	1.70E-01	Soluble	1.21E-01	Soluble	1.49E-01	Soluble
**8**	5.32E+01	Very soluble	3.83E+02	Highly soluble	9.66E-01	Soluble
**9**	5.83E-01	Soluble	1.03E+00	Soluble	4.17E-01	Soluble
**10**	5.64E-01	Soluble	4.54E-01	Soluble	7.26E-01	Soluble
**11**	1.02E-02	Moderately soluble	5.37E-03	Moderately soluble	1.21E-03	Moderately soluble
**12**	1.68E-01	Soluble	7.41E-02	Soluble	2.51E-01	Soluble
**13**	5.83E-02	Soluble	8.33E-03	Moderately soluble	4.34E-02	Soluble
**14**	1.49E+00	Soluble	4.58E+00	Very soluble	2.62E-02	Moderately soluble
**15**	1.01E-02	Moderately soluble	1.61E-02	Moderately soluble	1.80E-05	Poorly soluble
**16**	1.07E-03	Moderately soluble	6.95E-04	Moderately soluble	1.42E-05	Poorly soluble
**17**	1.28E+00	Soluble	3.48E+00	Soluble	6.24E-02	Soluble
Norfloxacin	1.62E+01	Very soluble	2.86E+02	Very soluble	6.31E-02	Soluble

Table S9. Pharmacokinetics of target compounds **2-17** and norfloxacin.

Molecule	GI absorption	BBB permeant	Pgp substrate	CYP1A2 inhibitor	CYP2C19 inhibitor	CYP2C9 inhibitor	CYP2D6 inhibitor	CYP3A4 inhibitor	log Kp (cm/s)
**2**	High	No	No	No	No	No	No	No	-7.44
**3**	High	No	Yes	No	No	No	No	No	-10.29
**4**	High	No	Yes	No	Yes	Yes	No	Yes	-7.12
**5**	Low	No	Yes	No	No	Yes	No	No	-9.27
**6**	High	No	No	No	No	No	No	No	-7.9
**7**	High	No	No	No	Yes	Yes	No	No	-7.3
**8**	High	No	Yes	No	No	No	No	No	-10.04
**9**	High	No	No	No	No	No	No	No	-7.57

**10**	High	No	Yes	No	No	No	No	No	-7.71
**11**	High	No	No	No	No	Yes	No	No	-6.6
**12**	High	No	No	No	No	Yes	No	No	-7.48
**13**	Low	No	Yes	No	No	Yes	No	No	-7.44
**14**	High	No	Yes	No	No	No	No	No	-8.53
**15**	High	Yes	Yes	Yes	Yes	Yes	Yes	Yes	-6.6
**16**	High	No	Yes	No	Yes	Yes	No	Yes	-6.2
**17**	High	No	Yes	No	No	No	No	No	-8.38
Norfloxacin	High	No	Yes	No	No	No	No	No	-8.98

Table S10. Drug likeness parameters of target compounds **2-17** and norfloxacin.

**Molecule**	**Lipinski #violations**	**Ghose #violations**	**Veber #violations**	**Egan #violations**	**Muegge #violations**	**Bioavailability Score**
**2**	0	0	0	0	0	0.56
**3**	0	0	0	0	1	0.56
**4**	0	1	0	0	0	0.56
**5**	2	2	1	1	0	0.56
**6**	0	0	0	0	0	0.56
**7**	0	0	0	0	0	0.56
**8**	0	0	0	0	1	0.56
**9**	0	0	0	0	0	0.56
**10**	0	0	0	0	0	0.56
**11**	0	0	0	0	0	0.56
**12**	0	0	0	0	0	0.56
**13**	0	0	1	1	0	0.56
**14**	0	0	0	0	0	0.55
**15**	0	0	0	0	0	0.55
**16**	0	2	0	0	0	0.55
**17**	0	0	0	0	0	0.55
**Norfloxacin**	0	0	0	0	0	0.55
